# Global burden of vision impairment due to age-related macular degeneration, 1990–2021, with forecasts to 2050: a systematic analysis for the Global Burden of Disease Study 2021

**DOI:** 10.1016/S2214-109X(25)00143-3

**Published:** 2025-06-25

**Authors:** GBD 2021 Global AMD Collaborators, Yi Deun Jeong, Seoyoung Park, Min Seo Kim, Sung Hwi Hong, Hasan Aalruz, Yohannes Habtegiorgis Abate, Rouzbeh Abbasgholizadeh, Samar Abd ElHafeez, Auwal Abdullahi, Richard Gyan Aboagye, Lucas Guimarães Abreu, Ahmed Abu-Zaid, Isaac Yeboah Addo, Habeeb Omoponle Adewuyi, Saira Afzal, Williams Agyemang-Duah, Aqeel Ahmad, Danish Ahmad, Sajjad Ahmad, Ali Ahmadi, Hooman Ahmadzadeh, Ali Ahmed, Ayman Ahmed, Haroon Ahmed, Syed Anees Ahmed, Amjad S Al Mosa, Rasmieh Mustafa Al-amer, Mohammed Albashtawy, Ahmad Samir Alfaar, Abdelazeem M Algammal, Fadwa Naji Alhalaiqa, Abid Ali, Syed Shujait Ali, Waad Ali, Ahmad Alrawashdeh, Awais Altaf, Vera L Alves Carneiro, Yaser Mohammed Al-Worafi, Hany Aly, Sofia Androudi, Boluwatife Stephen Anuoluwa, Saeid Anvari, Anayochukwu Edward Anyasodor, Jalal Arabloo, Mosab Arafat, Aleksandr Y Aravkin, Demelash Areda, Anton A Artamonov, Akram M Asbeutah, Seyyed Shamsadin Athari, Maha Moh'd Wahbi Atout, Alok Atreya, Lemessa Assefa A Ayana, Shahkaar Aziz, Ahmed Y. Azzam, Saeed Bahramian, Ruhai Bai, Atif Amin Baig, Soham Bandyopadhyay, Zarrin Basharat, Mohammad-Mahdi Bastan, Maryam Bemanalizadeh, Akshaya Srikanth Bhagavathula, Nikha Bhardwaj, Pankaj Bhardwaj, Sonu Bhaskar, Ajay Nagesh Bhat, Jasvinder Singh Bhatti, Fassikaw Kebede Bizuneh, Paul Svitil Briant, Gabrielle Britton, Yasser Bustanji, Zahid A Butt, Florentino Luciano Caetano dos Santos, Mehtap Çakmak Barsbay, Fan Cao, Vijay Kumar Chattu, Anis Ahmad Chaudhary, Patrick R Ching, Hitesh Chopra, Sonali Gajanan Choudhari, Dinh-Toi Chu, João M Coelho, Natalia Cruz-Martins, Omid Dadras, Xiaochen Dai, Emanuele D'Amico, Samuel Demissie Darcho, Ana Maria Dascalu, Nihar Ranjan Dash, Mohsen Dashti, Anna Dastiridou, Nikolaos Dervenis, Aragaw Tesfaw Desale, Vinoth Gnana Chellaiyan Devanbu, Amol S Dhane, Daniel Diaz, Michael J Diaz, Thanh Chi Do, Ojas Prakashbhai Doshi, Ashel Chelsea Dsouza, Hisham Atan Edinur, Ferry Efendi, Michael Ekholuenetale, Rabie Adel El Arab, Ibrahim Farahat El Bayoumy, Muhammed Elhadi, Chadi Eltaha, Mohammad Hassan Emamian, Adeniyi Francis Fagbamigbe, Ayesha Fahim, Hossein Farrokhpour, Ali Fatehizadeh, Timur Fazylov, Alireza Feizkhah, Ginenus Fekadu, Nuno Ferreira, Florian Fischer, Ida Fitriana, Ali Forouhari, Matteo Foschi, João M M Furtado, Blima Fux, Sridevi G, Muktar A Gadanya, Abhay Motiramji Gaidhane, Aravind P Gandhi, Balasankar Ganesan, Ravindra Kumar Garg, Rupesh K Gautam, Miglas Welay Gebregergis, Mesfin Gebrehiwot, Delaram J Ghadimi, Sadegh Ghafarian, Mahaveer Golechha, Pouya Goleij, Ayman Grada, Shi-Yang Guan, Snigdha Gulati, Sapna Gupta, Veer Bala Gupta, Vivek Kumar Gupta, Roberth Steven Gutiérrez-Murillo, Awoke Derbie Habteyohannes, Arvin Haj-Mirzaian, Sobia Ahsan Halim, Ahmed I Hasaballah, Md. Kamrul Hasan, Hamidreza Hasani, Jiawei He, Golnaz Heidari, Mojtaba Heydari, Nguyen Quoc Hoan, Ramesh Holla, Mehdi Hosseinzadeh, Chengxi Hu, Junjie Huang, Salman Hussain, Segun Emmanuel Ibitoye, Nayu Ikeda, Irena M Ilic, Milena D Ilic, Mustapha Immurana, Arit Inok, Lalu Muhammad Irham, Md. Rabiul Islam, Sheikh Mohammed Shariful Islam, Chidozie Declan Iwu, Louis Jacob, Ammar Abdulrahman Jairoun, Mihajlo Jakovljevic, Talha Jawaid, Shubha Jayaram, Zixiang Ji, Jost B Jonas, Nitin Joseph, Charity Ehimwenma Joshua, Vidya Kadashetti, Ankita Kankaria, Kehinde Kazeem Kanmodi, Neeti Kapoor, Ibraheem M Karaye, Soujanya Kaup, Gbenga A Kayode, Yousef Saleh Khader, Himanshu Khajuria, Ajmal Khan, Atulya Aman Khosla, Yun Jin Kim, Adnan Kisa, Shivakumar KM, Kewal Krishan, Mohammed Kuddus, Mukhtar Kulimbet, Nithin Kumar, Satyajit Kundu, Chandrakant Lahariya, Dharmesh Kumar Lal, Iván Landires, Van Charles Lansingh, Ariane Laplante-Lévesque, Caterina Ledda, Munjae Lee, Seung Won Lee, Wei-Chen Lee, Stephen S Lim, Xuefeng Liu, José Francisco López-Gil, Zheng Feei Ma, Kashish Malhotra, Vahid Mansouri, Roy Rillera Marzo, Alireza Mashaghi, Yasith Mathangasinghe, Andrea Maugeri, Asim Mehmood, Tesfahun Mekene Meto, Hadush Negash Meles, Endalkachew Belayneh Melese, Tomislav Mestrovic, Sachith Mettananda, Irmina Maria Michalek, Andreea Mirica, Abdalla Z Mohamed, Nouh Saad Mohamed, Abdollah Mohammadian-Hafshejani, Ali H Mokdad, Fateme Montazeri, Maryam Moradi, Rohith Motappa, Sumaira Mubarik, Kavita Munjal, Yanjinlkham Munkhsaikhan, Amin Nabavi, Ganesh R Naik, Vinay Nangia, Shumaila Nargus, Zuhair S Natto, Muhammad Naveed, Biswa Prakash Nayak, Athare Nazri-Panjaki, Van Thanh Nguyen, Robina Khan Niazi, Syed Toukir Ahmed Noor, Mamoona Noreen, Fred Nugen, Bogdan Oancea, Osamudiamen Cyril Obasuyi, Andrew T Olagunju, Sok King Ong, Michal Ordak, Verner N Orish, Mayowa O Owolabi, Jagadish Rao Padubidri, Georgios D Panos, Leonidas D Panos, Shahina Pardhan, Romil R Parikh, Sungchul Park, Tae Hwan Park, Maja Pasovic, Roberto Passera, Jay Patel, Shrikant Pawar, Prince Peprah, Arokiasamy Perianayagam, Mohsen Pourazizi, Jalandhar Pradhan, Jagadeesh Puvvula, Nameer Hashim Qasim, Venkatraman Radhakrishnan, Pankaja Raghav, Fakher Rahim, Vafa Rahimi-Movaghar, Mohammad Hifz Ur Rahman, Mosiur Rahman, Muhammad Aziz Rahman, Shayan Rahmani, Mohammad Rahmanian, Pushp Lata Rajpoot, Sowmya J Rao, Mohammad-Mahdi Rashidi, Salman Rawaf, Elrashdy M. Moustafa Mohamed Redwan, Mohsen Rezaeian, Sara Riaz, Moustaq Karim Khan Rony, Himanshu Sekhar Rout, Priyanka Roy, Aly M A Saad, Zahra Saadatian, Cameron John Sabet, Basema Ahmad Saddik, Umar Saeed, Sare Safi, Sher Zaman Safi, Fatemeh Saheb Sharif-Askari, Narjes Saheb Sharif-Askari, Amirhossein Sahebkar, Pragyan Monalisa Sahoo, S. Mohammad Sajadi, Mohamed A Saleh, Yoseph Leonardo Samodra, Abdallah M Samy, Tanmay Sarkar, Brijesh Sathian, Maheswar Satpathy, Jennifer Saulam, Monika Sawhney, Ganesh Kumar Saya, Siddharthan Selvaraj, Yashendra Sethi, Allen Seylani, Jaffer Shah, Amira A Shaheen, Samiah Shahid, Moyad Jamal Shahwan, Masood Ali Shaikh, Muhammad Aaqib Shamim, Javad Sharifi Rad, Anupam Sharma, Vishal Sharma, Maryam Shayan, Mahabalesh Shetty, Pavanchand H Shetty, Premalatha K Shetty, Mika Shigematsu, Aminu Shittu, Negussie Boti Sidamo, Emmanuel Edwar Siddig, Mithun Sikdar, Jasvinder A Singh, Paramdeep Singh, Puneetpal Singh, Surjit Singh, Raul A R C Sousa, Chandrashekhar T Sreeramareddy, Chandan Kumar Swain, Lukasz Szarpak, Seyyed Mohammad Tabatabaei, Ker-Kan Tan, Hugh R Taylor, Mohamad-Hani Temsah, Ramna Thakur, Jansje Henny Vera Ticoalu, Krishna Tiwari, Marcos Roberto Tovani-Palone, Ngoc Ha Tran, Thang Huu Tran, Munkhtuya Tumurkhuu, Saeed Ullah, Muhammad Umair, Sanaz Vahdati, Shaopan Wang, Muhammad Waqas, Nuwan Darshana Wickramasinghe, Kazumasa Yamagishi, Amir Yarahmadi, Pengpeng Ye, Arzu Yiğit, Yazachew Engida Engida Yismaw, Naohiro Yonemoto, Aurora Zanghì, Mohammed G M Zeariya, Zhi-Jiang Zhang, Claire Chenwen Zhong, Abzal Zhumagaliuly, Makan Ziafati, Magdalena Zielińska, Sa'ed H Zyoud, Jae Il Shin, Dong Keon Yon

## Abstract

**Background:**

Age-related macular degeneration (AMD) is a growing public health concern worldwide, as one of the leading causes of vision impairment. We aimed to estimate global, national, and region-specific prevalence and disability-adjusted life-years (DALYs) along with tobacco as a modifiable risk factor to aid public policy addressing AMD.

**Methods:**

Data on AMD were extracted from the Global Burden of Disease, Injuries, and Risk Factor Study 2021 database in 204 countries and territories, 1990–2021. Vision impairment was defined and categorised by severity as follows: moderate to severe vision loss (visual acuity from <6/18 to 3/60) and blindness (visual acuity <3/60 or a visual field <10 degrees around central fixation). The burden of vision impairment attributable to AMD was subsequently estimated. These estimates were further stratified by geographical region, age, year, sex, Healthcare Access and Quality (HAQ) Index, and Socio-demographic Index (SDI) levels. Additionally, the effect of tobacco use, a modifiable risk factor, on the burden of AMD was analysed, and projections of AMD burden were estimated through to 2050. These projections also included scenario modelling to assess the potential effects of tobacco elimination.

**Findings:**

Globally, the number of individuals with vision impairment due to AMD more than doubled, rising from 3·64 million (95% uncertainty inverval [UI] 3·04–4·35) in 1990 to 8·06 million (6·71–9·82) in 2021. Similarly, DALYs increased by 91% over the same period, from 0·30 million (95% UI 0·21–0·42) to 0·58 million (0·40–0·80). By contrast, age-standardised prevalence and DALY rates declined, with prevalence rates decreasing by 5·53% (99·50 per 100 000 of the population [95% UI 83·16–118·04] in 1990 to 94·00 [78·32–114·42] in 2021) and DALY rates dropping by 19·09% (8·38 [5·70–11·53] to 6·78 [4·70–9·32]). These rates showed a consistent decrease in higher SDI quintiles, reflecting the negative correlation between HAQ Index and AMD burden. A general downward trend was observed from 1990 to 2021, with the largest age-standardised reduction occurring in the low-middle SDI quintile. The global contribution of tobacco to age-standardised DALYs decreased by 20%, declining from 12·45% (95% UI 7·73–17·37) in 1990 to 9·96% (6·12–14·06) in 2021. By 2050, the number of individuals affected by AMD is projected to increase from 3·40 million males (95% UI 2·81–4·17) in 2021 to 9·02 million (5·72–14·20) and from 4·66 million females (3·88–5·65) to 12·32 million (8·88–17·08). Eliminating tobacco use could reduce these numbers to 8·17 million males (5·59–11·92) and 11·15 million females (8·58–14·48) in 2050.

**Interpretation:**

While the total prevalence and DALYs due to AMD have steadily increased from 1990 to 2021, age-standardised prevalence and DALY rates have declined, probably reflecting the effect of population ageing and growth. The consistent decrease in age-standardised rates with higher SDI levels highlights the crucial role of health-care resources and public policies in mitigating AMD-related vision impairment. The downward trend observed from 1990 to 2021 might also be partially attributed to the reduced effect of tobacco as a modifiable risk factor, with declines in tobacco use seen globally and across all SDI quintiles. The burden of vision impairment due to AMD is projected to increase to about 21·34 million in 2050. However, effective tobacco regulation has the potential to substantially reduce AMD-related vision impairment, particularly in lower SDI quintiles where health-care resources are limited.

**Funding:**

Gates Foundation.

## Introduction

Age-related macular degeneration (AMD) is a degenerative disease that occurs with ageing, with symptoms ranging from asymptomatic to vision impairment, and further progressing to blindness.^1^ AMD, the third leading cause of vision impairment,^2^ is known to increase the risk of depression by 15%.^3^ Moreover, vision impairment due to AMD contributes to increased all-cause mortality^4^ and diminishes quality of life,^5^ while also exerting a national impact on productivity decline.^6^ Given its potential effect on individual health and broader societal outcomes, it is imperative to accurately estimate the extent of AMD's effect within societies undergoing population growth and ageing worldwide.

**Research in context**

**Evidence before this study**

The growing and ageing population worldwide has led to the increased attention to the burden of age-related macular degeneration (AMD). We conducted a comprehensive search of PubMed and MEDLINE on May 1, 2024, without language restrictions, covering studies published from database inception up to Nov 30, 2024, using the terms (“age-related macular degeneration” AND “prevalence” OR “DALYs”). The 2014 meta-analysis by Wong and colleagues estimated the overall prevalence of AMD without addressing the specific prevalence of vision impairment caused by AMD, nor did it provide data at the country or regional level. Other studies have focused on the burden of AMD in specific regions, often restricted to high-income countries. Additionally, while the Global Burden of Disease, Injuries, and Risk Factors Study (GBD) 2019 Blindness and Vision Impairment Collaborators presented the prevalence of vision impairment due to AMD, they did not include data related to disability-adjusted life-years (DALYs). Previous investigations categorised tobacco as an established risk factor, whereas our study undertook a comprehensive analysis of tobacco's effect as a modifiable risk factor on DALYs. Consequently, to our knowledge no other study has fully explored the burden of AMD from both demographic and geographical perspectives.

**Added value of this study**

This study assessed the prevalence and DALYs associated with vision impairment caused by AMD across 204 countries and territories from 1990 to 2021, using robust data from the GBD 2021. Globally, vision impairment due to AMD affected 8·06 million (95% uncertainty interval 6·71–9·82) individuals in 2021. This study further analysed the burden by incorporating demographic factors, the Healthcare Access and Quality Index, and the Socio-demographic Index (SDI) to provide the burden in depth. Tobacco use, an established risk–outcome pair for AMD, was included as a contributing risk factor to the burden of AMD-related vision impairment. The findings suggest that tobacco regulation may have contributed to reducing DALYs associated with AMD. Projections indicate that the number of affected individuals will rise to 21·34 million by 2050; however, eliminating tobacco use could reduce this burden by approximately 19·32 million, emphasising the importance of targeted tobacco control measures.

**Implications of all the available evidence**

Despite the overall declining trend in the prevalence and DALYs of vision impairment due to AMD, it remains a significant burden, particularly for females, older age groups, and lower SDI quintiles. Given the unequal distribution of medical resources across SDI groups, there is a requirement for alternative strategies beyond novel treatments such as anti-vascular endothelial growth factors, including a focus on controlling risk factors associated with AMD-related vision impairment. The findings of this study suggest that tobacco regulation could be an effective strategy for reducing the burden of AMD, with projections indicating that such interventions would be particularly beneficial in the future, especially in lower SDI quintiles. Furthermore, tailored interventions for different SDI quintiles, including cost-effective medical treatments and dietary improvements, would help address the burden of AMD. Future studies should focus on identifying additional risk factors for AMD and developing effective policies and interventions to reduce disparities in the burden of AMD across SDI groups.

Previous research on the global burden of AMD, primarily systematic reviews, has highlighted the heterogeneity of data.^7^ Many previous studies estimating AMD prevalence have focused on specific regions or high-income countries,^8^ making it challenging to assess the burden of AMD in low-income to middle-income countries (LMICs). Furthermore, given the restricted access to treatment in LMICs,^9^ the burden of AMD is notably disproportionate on a global scale, underscoring the need for further research including such regions for a comprehensive global analysis.

The influence of specific risk factors on AMD remains contentious. While the detrimental effects of tobacco and the potential benefits of antioxidant intake are relatively established,^10^ the association with other factors vary across studies and is subject to ongoing debate. Nevertheless, despite the well documented global impact of tobacco on AMD, there is a notable gap in the literature concerning the analysis of tobacco's contribution to disability-adjusted life-years (DALYs) based on geographical location and Socio-demographic Index (SDI; a measure that captures income per capita, education, and fertility).

AMD should be addressed as a paramount global public health concern owing to its prominence as a leading cause of irreversible vision impairment on a global scale.^11^ This study aimed to estimate the prevalence and DALYs of AMD from 1990 to 2021, stratified by age, sex, location, and SDI levels, while also quantifying the burden attributable to tobacco use. Additionally, the study sought to provide deeper insights into the global burden of AMD by projecting trends through to 2050 and analysing the potential effect of eliminating tobacco as a risk factor, using data from the Global Burden of Disease, Injuries, and Risk Factors Study (GBD) 2021.

This manuscript was produced as part of the GBD Collaborator Network and in accordance with the GBD Protocol.^12^, ^13^

## Methods

### Overview

The GBD 2021 integrates an extensive and expanding array of data sources, encompassing surveys, censuses, vital statistics, and other health-related databases. These diverse datasets were harnessed to estimate morbidity, illness, injury, and attributable risk across 204 countries and territories from 1990 to 2021.^12^, ^13^ Incidence, prevalence, years lived with disability (YLDs), and DALYs were calculated for the period spanning 1990 to 2021.^12^, ^13^ The 2021 GBD methodologies have been meticulously developed and improved upon over several iterations, benefiting from the rigorous research conducted in preceding iterations of the GBD studies.^14^ This study adheres to the GATHER reporting guidelines. Further details on methods are outlined in appendix 1 and appendix 2.

### Input data and case definition

Data preparation was conducted through a systematic review by the Vision Loss Expert Group (VLEG) of population-based studies on vision impairment and blindness, supplemented by grey literature sources. Eligible studies were combined with data from the Rapid Assessment of Avoidable Blindness (RAAB) studies, the US National Health and Nutrition Examination Survey, and the WHO Study on Global Ageing and Adult Health, with support from the GBD collaborators.

The VLEG commissioned the York Health Economics Consortium (York, UK) to perform a comprehensive literature review using databases such as Embase, SciELO, PubMed, MEDLINE, WHOLIS, and Open Grey. Titles and abstracts were screened, and quality assessments were conducted by regional VLEG committees to make final inclusion decisions. The detailed search keywords and methodology are described in a previous GBD study.^15^ Additional data included 5-year age-disaggregated RAAB surveys obtained from the RAAB repository. Population-representative data, primarily from national and subnational cross-sectional surveys, were used for modelling cause-specific vision impairment. RAAB surveys, which focus on individuals 50 years and older, were crucial for data collection in LMIC regions, ensuring a comprehensive and reliable analysis (appendix 1 pp 11–13). The methodology for RAAB surveys is detailed in a previous study.^16^

Studies were included based on the following two criteria: first, visual acuity had to be measured using a test chart compatible with the Snellen scale, and second, the sample needed to be representative of the general population. Studies relying on self-reported vision loss were excluded. Vision impairment was categorised according to the ICD 11th edition, which defines three severity levels: moderate vision loss (visual acuity ≥6/60 and <6/18), severe vision loss (visual acuity ≥3/60 and <6/60), and blindness (visual acuity <3/60 or a visual field of less than 10° around central fixation). The definition of AMD pertains to the age-related deterioration of the macula, the central portion of the retina responsible for central vision, resulting in central vision loss (appendix 1 pp 10–11).

### Data preparation and processing

First, raw data were separated into datasets encompassing the GBD-defined vision loss envelopes for all-cause moderate and severe vision impairment, as well as blindness. A mixed-effects meta-regression tool, developed by the Institute for Health Metrics and Evaluation and known as MR-BRT (Meta Regression–Bayesian, Regularised, Trimmed), was used to adjust non-reference data to align with the reference definition of presenting vision data within the WHO severity categories. MR-BRT is specifically designed to account for between-study heterogeneity as part of uncertainty adjustments and to trim outlier input data, thereby improving the reliability of the estimates. This adjustment process was applied to data from studies that did not use RAAB methods. Data collected using the RAAB methodology were further adjusted to align with comprehensive surveys to act as the reference definitions using the same adjustment factors applied to all-cause moderate and severe vision impairment and blindness (appendix 1 pp 13–16). This approach was adopted because a substantially larger volume of data was available for all-cause moderate vision impairment, severe vision impairment, and blindness compared with data for individual specific causes. This allowed for the development of more data-rich models, ensuring robust estimates across these categories. The methodological details are presented in previous studies.^15^, ^17^

### Disease Modelling Meta-Regression 2.1 estimation

In the GBD 2021 the estimation of non-fatal impairment due to macular degeneration was conducted using the Disease Modelling Meta-Regression 2.1 (DisMod-MR 2.1) model, a Bayesian mixed-effects meta-regression tool designed for non-fatal disease modelling. This method, described in detail elsewhere,^13^ employs a compartmental model with age integration to ensure consistency across all disease parameters using differential equations with appropriate boundary conditions (appendix 1 pp 17–19). For vision-related diseases such as macular degeneration, which do not have a fatal component, DisMod-MR 2.1 focuses exclusively on estimating prevalence. The model incorporates an offset log-normal approach with fixed effects for location-specific covariates and random effects to account for variations across locations. Input data from all locations are integrated into a mixed-effects non-linear model to produce a global estimate of disease burden. The outputs of this global model (including global fit, fixed effects, and random effects) are cascaded as priors to generate estimates for GBD super-regions, regions, countries, and subnational areas within 21 countries. This hierarchical cascade approach ensures consistency across all geographical levels.

### Modelling and post processing

The estimation of YLDs for macular degeneration followed a structured approach using DisMod-MR 2.1 and accounted for location-specific covariates with fixed effects and variations across locations through random effects. After using DisMod-MR 2.1 to generate prevalence estimates for vision loss severity categories, these estimates were used to calculate YLDs by multiplying prevalence with corresponding disability weights, which measure the degree of health impairment on a 0 to 1 scale (0 for perfect health to 1 for death-equivalent health loss). Uncertainty was quantified using 500 comorbidity-corrected YLDs and disability weight samples, assuming no correlation between the two, with 95% uncertainty intervals (UIs) determined from the resulting distribution. This hierarchical approach ensured robust, location-specific estimates of YLDs, incorporating the latest prevalence data and disability weights while adjusting for comorbidity. Further detail is provided in appendix 1 (pp 20–24).

### Forecasting model for 2050

The final estimates for GBD 2021 cover the years 1990 to 2021. Prevalence for non-fatal conditions, where mortality was not observed, was primarily modelled using linear mixed-effects models to directly estimate prevalence. For forecasting, a generalised ensemble modelling approach was applied, incorporating 12 distinct submodels based on two primary strategies: the annualised rate of change and a two-stage spline model using the MR-BRT. Each submodel employed six recency-weighting parameters (Ω), ranging from 0 to 2·5, with higher values assigning greater weight to more recent data. The ensemble model's performance was evaluated using root-mean-square error. Additionally, population attributable fractions were incorporated to account for the influence of risk factors, enabling forecasts for global, sex-specific scenarios under two different versions. Further methodological details are provided in appendix 1 (pp 26–35).

### Risk factors

The GBD 2021 introduced several advancements in estimating risk exposure levels, relative risks, and attributable burdens compared with previous GBDs. For 211 risk–outcome pairs, the burden-of-proof risk function analysis was used to address unexplained heterogeneity across studies in the input data, providing a more conservative interpretation of risk–outcome associations. To facilitate the interpretation and comparison of burden-of-proof risk function metrics across risk factors, summary risk–outcome scores were calculated and mapped to a star rating system (ranging from one star to five stars) to summarise the strength of evidence for these associations. These updates incorporated findings from new or revised systematic reviews conducted since 2019. Overall, the GBD 2021 risk factor analysis used data from 54 561 distinct sources to generate epidemiological estimates for 88 risk factors and 631 associated risk–outcome pairs. Risk factors were classified within a hierarchical structure comprising four levels, with level one categorised into environmental and occupational, behavioural, and metabolic risks. Each risk factor was further subdivided into more specific categories at subsequent levels.

For macular degeneration, tobacco use—classified as a level one behavioural risk and level two specific risk—was the only risk factor that met the inclusion criteria for analysis. Tobacco remained a validated risk factor for AMD in both GBD 2019 and GBD 2021, despite the introduction of more rigorous and updated evaluation methodologies. Other potential risk factors for AMD did not meet the GBD inclusion thresholds, highlighting the need for further research to better establish their relevance. The assessment of all risk–outcome pairs followed stringent criteria established by the GBD collaborators, with continual updates to ensure consistency with emerging scientific evidence. Comprehensive methodological details regarding these evaluations are provided in appendix 1 (pp 36–42).

### Healthcare Access and Quality Index

This study examines health-care access and quality for AMD within defined age groups, based on the Organisation for Economic Co-operation and Development definition of the working-age population (age 15–64 years) and Nolte and McKee's avoidable mortality age limit of 74 years.^18^ The Healthcare Access and Quality (HAQ) Index quantifies health-care performance on a scale from 0 to 100, where 0 represents the lowest observed performance and 100 the highest performance. The Index standardises the effects of cause-specific factors and risk exposures through an average weighting system to allow meaningful comparisons across age groups, years, and countries.^19^ Separate HAQ Index estimates were calculated for each country, year, and age group. Additionally, the study assessed convergence trends between high-SDI countries and those in lower SDI quintiles to evaluate progress in improving health-care access and quality over time. Further methodological details are provided in appendix 1 (pp 42–45).

### Role of the funding source

The funder of the study had no role in study design, data collection, data analysis, data interpretation, or writing of the report.

## Results

### Global burden of vision impairment due to AMD

**Figure 1:**
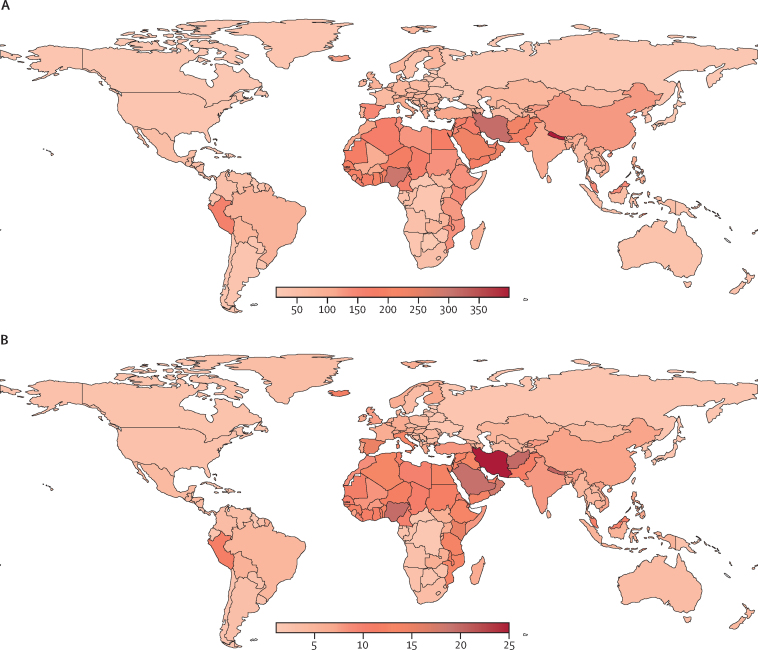
Global distribution of age-related macular degeneration in both sexes, 2021 Age-standardised prevalence (A) and DALY rate (B) per 100 000 of the population, 2021. DALY=disability-adjusted life-year.

163 RAAB surveys and 107 non-RAAB studies were used to estimate vision impairment attributable to AMD. The distribution of these studies by country, categorised as RAAB and non-RAAB studies, is presented in appendix 1 (pp 11–13). The age-standardised prevalence and DALY rates of AMD at the regional level are illustrated in figure 1; the table presents the AMD rates for 1990, 2021, and the annual percentage change.

In 2021, the western sub-Saharan Africa region showed the highest age-standardised prevalence rate (229·48 [95% UI 185·45 to 281·21] per 100 000 of the population), followed by the North Africa and Middle East region (177·95 [144·80 to 218·13]), and the east Asia region (121·81 [100·23 to 148·40]). The western sub-Saharan Africa region also showed the highest age-standardised DALY rate (14·73 [95% UI 10·43 to 20·56] per 100 000 of the population), followed by the North Africa and Middle East region (14·04 [9·60 to 9·61]) and eastern sub-Saharan Africa (11·11 [7·39 to 15·78]; figure 1 and table). Globally, age-standardised prevalence rates decreased by 5·53% (–8·84 to –2·23) from 1990 (99·50 [83·16 to 118·04]) to 2021 (94·00 [78·32 to 114·42]), while age-standardised DALY rates decreased by 19·09% (–22·56 to –15·42) over the same period (8·38 [5·70 to 11·53] in 1990 to 6·78 [4·70 to 9·32] in 2021; table). Regionally, an increase in age-standardised prevalence rates compared with 1990 was observed only in central sub-Saharan Africa and southern sub-Saharan Africa, with increases of 5·42% and 9·13%, respectively. However, no rise in age-standardised DALY rates compared to 1990 was noted in any region (table).

**Table:** Global age-standardised rates per 100 000 of the population and annual percentage change of prevalence and DALYs for age-related macular degeneration, 1990–2021

		**Prevalence**			**DALYs**		
		1990 (95% UI)	2021 (95% UI)	Annual percentage change between 1990 and 2021, % (95% UI)	1990 (95% UI)	2021 (95% UI)	Annual percentage change between 1990 and 2021, % (95% UI)
Global		99·50 (83·16 to 118·04)	94·00 (78·32 to 114·42)	−5·53% (−8·84 to −2·23)	8·38 (5·70 to 11·53)	6·78 (4·70 to 9·32)	−19·09% (−22·56 to −15·42)
Sex							
	Male	92·23 (76·87 to 110·83)	87·10 (71·99 to 106·29)	−5·56% (−8·82 to −2·45)	7·37 (5·01 to 10·24)	6·01 (4·15 to 8·29)	−18·48% (−22·13 to −14·71)
	Female	104·34 (87·50 to 123·10)	99·53 (82·98 to 120·72)	−4·62% (−8·05 to −1·13)	9·04 (6·16 to 12·45)	7·38 (5·11 to 10·12)	−18·40% (−21·97 to −14·49)
SDI regions							
	Low SDI	144·70 (120·56 to 171·58)	139·92 (114·54 to 171·01)	−3·31% (−7·20 to 1·30)	11·90 (8·19 to 16·58)	10·08 (6·91 to 13·86)	−15·31% (−19·41 to −10·62)
	Low-middle SDI	138·32 (114·13 to 165·93)	104·67 (85·46 to 127·94)	−24·32% (−27·12 to −21·46)	11·43 (7·79 to 16·04)	7·64 (5·28 to 10·75)	−33·21% (−36·41 to −30·04)
	Middle SDI	114·58 (94·89 to 138·84)	107·56 (88·68 to 131·24)	−6·13% (−9·27 to −3·19)	8·65 (5·85 to 12·13)	7·26 (5·00 to 9·96)	−16·09% (−19·70 to −12·03)
	High-middle SDI	100·42 (84·93 to 118·77)	104·55 (87·88 to 126·60)	4·11% (−0·84 to 9·36)	8·65 (5·88 to 11·82)	7·40 (5·15 to 10·14)	−14·47% (−19·11 to −9·26)
	High SDI	56·98 (47·61 to 67·51)	48·43 (40·55 to 57·77)	−15·01% (−17·78 to −12·16)	5·48 (3·68 to 7·35)	4·08 (2·76 to 5·49)	−25·57% (−28·63 to −22·40)
Central Europe, eastern Europe, and central Asia							
	Central Asia	77·20 (61·54 to 96·52)	74·59 (59·75 to 93·95)	−3·38% (−6·78 to −0·32)	5·16 (3·56 to 7·16)	4·76 (3·25 to 6·70)	−7·68% (−13·47 to −2·20)
	Central Europe	64·32 (52·90 to 77·73)	60·30 (49·02 to 74·97)	−6·25% (−9·45 to −3·41)	4·72 (3·22 to 6·53)	4·03 (2·77 to 5·61)	−14·63% (−18·78 to −9·96)
	Eastern Europe	27·07 (22·15 to 32·65)	24·51 (20·03 to 29·61)	−9·46% (−12·21 to −6·73)	2·35 (1·54 to 3·27)	1·93 (1·28 to 2·69)	−17·84% (−22·39 to −12·56)
High-income regions							
	Australasia	44·46 (36·72 to 52·45)	37·76 (30·65 to 44·98)	−15·05% (−20·13 to −9·71)	4·83 (3·16 to 6·86)	3·73 (2·43 to 5·22)	−22·76% (−30·65 to −13·96)
	High-income Asia Pacific	23·69 (19·74 to 28·57)	20·57 (17·16 to 24·97)	−13·19% (−16·41 to −10·20)	2·51 (1·60 to 3·50)	1·99 (1·31 to 2·76)	−20·67% (−24·92 to −16·21)
	High-income North America	29·77 (24·90 to 35·14)	27·30 (22·77 to 32·60)	−8·30% (−10·33 to −6·31)	3·01 (2·03 to 4·16)	2·58 (1·77 to 3·52)	−14·25% (−17·55 to −10·55)
	Southern Latin America	38·04 (30·42 to 46·34)	32·32 (25·89 to 39·45)	−15·02% (−19·86 to −10·40)	3·74 (2·39 to 5·25)	2·79 (1·82 to 3·85)	−25·33% (−32·51 to −16·55)
	Western Europe	107·40 (89·61 to 126·02)	85·55 (71·62 to 101·13)	−20·34% (−23·14 to −17·36)	11·76 (7·83 to 15·93)	8·49 (5·68 to 11·35)	−27·79% (−30·72 to −24·34)
Latin America and Caribbean							
	Andean Latin America	118·47 (95·50 to 146·51)	113·60 (92·60 to 140·13)	−4·11% (−10·89 to 2·98)	9·28 (6·33 to 12·97)	7·92 (5·46 to 11·00)	−14·64% (−23·03 to −6·01)
	Caribbean	26·10 (20·88 to 32·83)	23·58 (18·71 to 29·36)	−9·64% (−12·98 to −5·47)	2·22 (1·44 to 3·10)	1·78 (1·17 to 2·50)	−19·89% (−27·19 to −12·30)
	Central Latin America	57·92 (47·41 to 70·66)	50·91 (41·44 to 63·41)	−12·11% (−15·43 to −8·78)	4·97 (3·41 to 6·89)	3·84 (2·61 to 5·29)	−22·67% (−26·98 to −18·19)
	Tropical Latin America	81·43 (67·39 to 98·71)	80·30 (65·95 to 98·34)	−1·40% (−4·85 to 2·39)	4·85 (3·41 to 6·64)	4·44 (3·13 to 6·09)	−14·25% (−17·55 to −10·55)
North Africa and Middle East		186·36 (151·99 to 221·21)	177·95 (144·80 to 218·13)	−4·51% (−8·91 to −0·44)	16·73 (11·36 to 23·55)	14·04 (9·60 to 19·61)	−16·07% (−19·90 to −11·63)
South Asia		157·88 (129·30 to 191·03)	113·88 (93·23 to 139·40)	−27·87% (−31·04 to −24·81)	13·12 (8·83 to 18·69)	8·23 (5·67 to 11·51)	−37·31% (−40·84 to −33·44)
Southeast Asia, east Asia, and Oceania							
	East Asia	118·72 (97·65 to 144·26)	121·81 (100·23 to 148·40)	2·60% (−0·72 to 6·14)	7·23 (5·10 to 10·02)	7·04 (4·89 to 9·69)	−2·61% (−6·41 to 2·09)
	Oceania	37·83 (30·17 to 46·91)	32·84 (26·10 to 41·15)	−13·20% (−17·48 to −8·77)	3·70 (2·41 to 5·36)	2·87 (1·88 to 4·10)	−22·53% (−29·92 to −14·25)
	Southeast Asia	96·28 (79·45 to 114·03)	77·01 (63·35 to 92·62)	−20·02% (−24·94 to −14·78)	8·51 (5·57 to 12·16)	5·82 (3·92 to 8·26)	−31·58% (−35·77 to −26·81)
Sub-Saharan Africa							
	Central sub-Saharan Africa	31·73 (25·32 to 39·93)	33·45 (26·83 to 42·30)	5·42% (0·89 to 10·36)	2·00 (1·32 to 2·81)	2·02 (1·36 to 2·89)	1·30% (−7·94 to 11·68)
	Eastern sub-Saharan Africa	129·97 (106·45 to 152·97)	116·04 (94·08 to 138·20)	−10·72% (−15·06 to −5·54)	14·52 (9·78 to 20·28)	11·11 (7·39 to 15·78)	−23·52% (−27·30 to −19·29)
	Southern sub-Saharan Africa	40·87 (33·37 to 50·23)	44·60 (36·73 to 54·69)	9·13% (6·25 to 12·20)	3·19 (2·12 to 4·50)	3·28 (2·21 to 4·59)	2·67% (−2·42 to 7·83)
	Western sub-Saharan Africa	226·01 (185·74 to 273·72)	229·48 (185·45 to 281·21)	1·54% (−2·31 to 5·45)	15·46 (11·10 to 21·22)	14·73 (10·43 to 20·56)	−16·07% (−19·90 to −11·63)

DALYs=disability-adjusted life-years. SDI=Socio-demographic Index. UI=uncertainty interval.

In 2021, the number of cases was highest in the east Asia region (2·68 million [95% UI 2·21–3·30]), followed by the south Asia region (1·58 million [1·29–1·96]), and the western Europe region (0·94 million [0·79–1·11]; appendix 1 pp 52–53). The numbers of DALYs were also highest in the east Asia region (0·15 million [0·11–0·21]), followed by the south Asia region (0·11 million [0·08–0·16]), and the western Europe region (0·09 million [0·06–0·13]; appendix 1 pp 54–55). Globally, the number of individuals with vision impairment due to AMD more than doubled, rising from 3·64 million (95% UI 3·04–4·35) in 1990 to 8·06 million (6·71–9·82) in 2021 (appendix 1 pp 56–58). Similarly, the number of DALYs increased by 91% during the same period, from 0·30 million (95% UI 0·21–0·42) in 1990 to 0·58 million (0·40–0·80) in 2021 (appendix 1 pp 59–60).

### Moderate to severe vision loss and blindness due to AMD

The total prevalence of vision impairment due to AMD, categorised by moderate vision loss, severe vision loss, and blindness, showed notable increases between 1990 and 2021. Cases of moderate vision loss rose from 2·13 million (95% UI 1·64–2·76) in 1990 to 5·39 million (4·18–7·00) in 2021, severe vision loss increased from 0·39 million (0·28–0·51) to 0·79 million (0·58–1·03), and blindness rose from 1·12 million (0·84–1·47) to 1·88 million (1·41–2·46). By contrast, age-standardised rates revealed varying trends. Per 100 000 of the population, age-standardised rates increased for moderate vision loss (56·98 [95% UI 44·40–72·90] in 1990 to 62·46 [48·59–80·76] in 2021), but declined for severe vision loss and blindness (10·49 [7·68–13·55] to 9·22 [6·81–11·84] and 32·02 [24·57–41·51] to 22·32 [16·81–29·05], respectively) over the same period (appendix 1 pp 61–63).

The number of YLDs also increased across all categories. YLDs from moderate vision loss rose from 0·06 million (95% UI 0·04–0·10) in 1990 to 0·16 million (0·09–0·26) in 2021; YLDs from severe vision loss rose from 0·07 million (0·04–0·10) to 0·14 million (0·09–0·21); and YLDs from blindness rose from 0·20 million (0·12–0·30) to 0·34 million (0·20–0·50). Age-standardised rates per 100 000 of the population showed a different pattern, with moderate vision loss rates increasing slightly (1·71 [0·97–2·75] in 1990 to 1·88 [1·07–3·01] in 2021), while rates for severe vision loss and blindness declined (1·84 [1·18–2·79] to 1·62 [1·03–2·44] and 5·74 [3·48–8·52] to 4·00 [2·43–5·93], respectively; appendix 1 pp 64–65).

### Burden of vision impairment due to AMD according to age and sex

**Figure 2:**
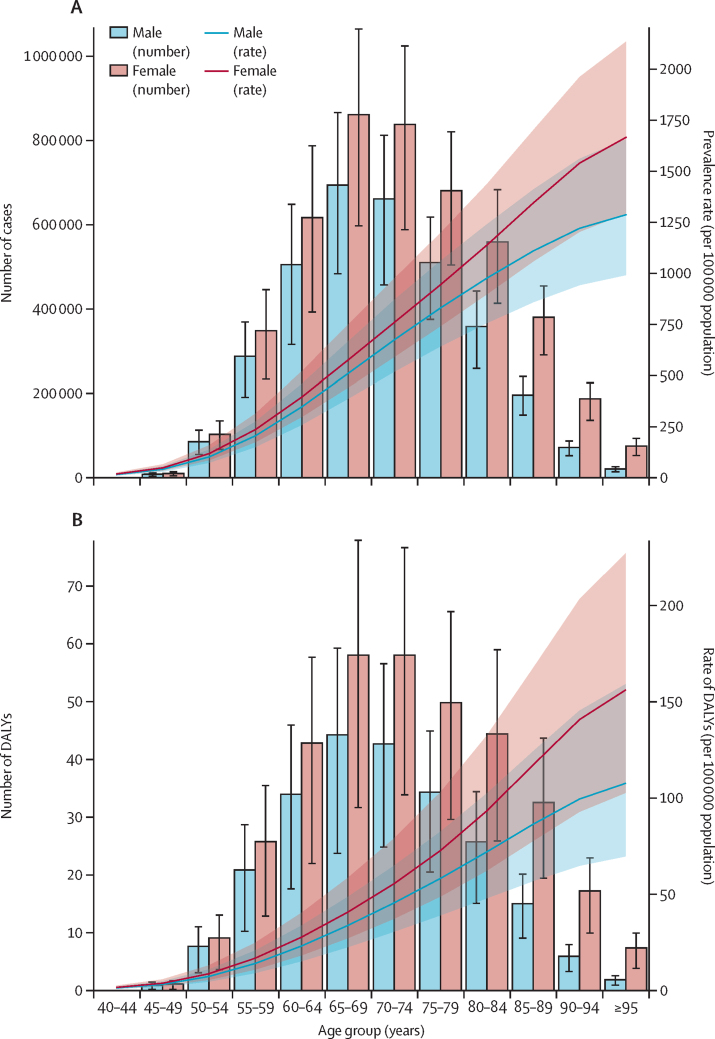
Rate per 100 000 of the population of prevalence (A) and DALYs (B) from age-related macular degeneration at the global level by age group and sex, 2021 Shaded regions indicate 95% uncertainty intervals. DALYs=disability-adjusted life-years.

The results of the subgroup analysis are shown in figure 2 and appendix 1 (pp 66–67). Across all age groups and measures, females exhibited a higher burden of AMD than males.

In 2021, the prevalence in females was higher in both number and rate, with 4·66 million cases (95% UI 3·88–5·65) with a rate of 118·46 per 100 000 of the population (95% UI 98·73–143·71). In contrast, males had 3·40 million cases (2·81–4·17) and a rate of 85·86 per 100 000 of the population (70·93–105·25). Similarly, females showed higher DALY counts and rates, with 3·35 million cases (2·39–4·74) with a rate of 8·78 per 100 000 of the population (95% UI 6·09–12·05). For males, the DALY count was 2·33 million DALYs (1·60–3·23), and the rate was 5·87 per 100 000 of the population (4·04–8·16). The highest prevalence and DALY counts were observed in the age 65–69 years group, with 1·55 million cases (95% UI 1·18–2·03) and 1·02 million DALYs (0·07–1·48), respectively. An increasing trend in prevalence and DALY rates was noted with advancing age for both sexes (figure 2; appendix 1 pp 66–67).

### Burden of vision impairment due to AMD according to SDI and correlation with the HAQ Index

Total counts of prevalence and DALYs, stratified by SDI quintiles from 1990 to 2021, are presented in appendix 1 (pp 68–75). In 2021, the highest prevalence of AMD and highest DALYs were observed in the high-middle SDI quintile (2·10 million cases [95% UI 1·76–2·56] and 0·15 million [0·10–0·20], respectively).

**Figure 3:**
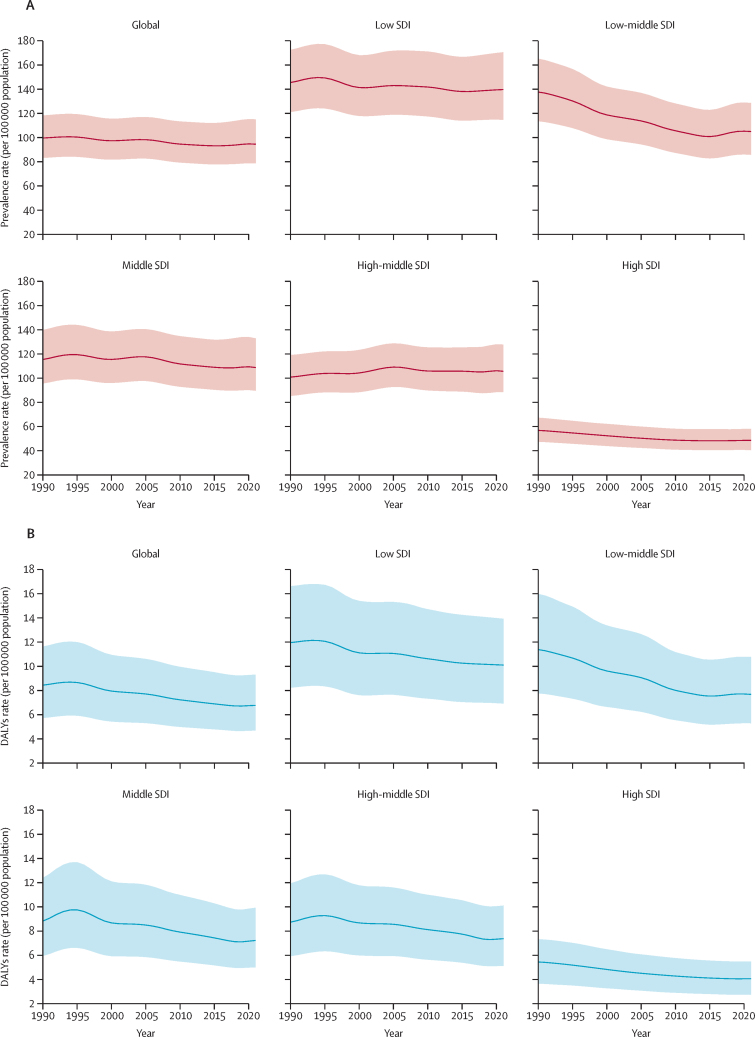
Age-standardised prevalence rate (A) and DALYs (B) per 100 000 of the population from age-related macular degeneration for both sexes by SDI, 2021 Shaded regions indicate 95% uncertainty intervals. DALYs=disability-adjusted life-years. SDI=Socio-demographic Index.

Despite the higher total counts in the high-middle SDI quintile, age-standardised prevalence and DALY rates tended to decrease with higher SDI quintiles. In 2021, age-standardised prevalence rates were highest in the low SDI quintile (139·92 per 100 000 of the population [95% UI 114·54–171·01]). Low-middle SDI, middle SDI, and high-middle SDI exhibited similar rates (104·67 [85·46–127·94], 107·56 [88·68–131·24], and 104·55 [87·88–126·60], respectively). The high SDI quintile showed the lowest prevalence rate of 48·43 (40·55–57·77; figure 3A; appendix 1 pp 72–73). A similar pattern was observed for age-standardised DALY rates. The low SDI quintile had a highest rate of 10·08 per 100 000 of the population (95% UI 6·91–13·86), while the high SDI quintile had the lowest rate (4·08 [2·76–5·49]). Rates for low-middle, middle, and high-middle SDI quintiles were 7·64 (5·28–10·75), 7·26 (5·00–9·96), and 7·40 (5·15–10·14), respectively (figure 3B and appendix 1 pp 74–75).

From 1990 to 2021, age-standardised prevalence and DALY rates exhibited a general downward trend across all SDI quintiles. The largest prevalence decrease occurred in the low-middle SDI quintile (138·32 per 100 000 of the population [95% UI 114·13–165·93] in 1990 to 104·67 [85·46–127·94] in 2021). Decreases were also observed in the high SDI quintile (56·98 [47·61–67·51] to 48·43 [40·55–57·77]), the middle SDI quintile (114·58 [94·89–138·84] to 107·56 [88·68–131·24]), the low-middle SDI quintile (138·32 [114·13–165·93] to 104·67 [85·46–127·94]), and the low SDI quintile (144·70 [120·56–171·58] to 139·92 [114·54–171·01]). By contrast, the high-middle SDI quintile showed a slight increase (100·42 [84·93–118·77] to 104·55 [87·88–126·60]; figure 3A; appendix 1 pp 74–75). The largest decrease in age-standardised DALY rates occurred in the low-middle SDI quintile (11·43 per 100 000 of the population [95% UI 7·79–16·04] in 1990 to 7·64 [5·28–10·75] in 2021). Decreases were also observed in the high SDI quintile (5·48 [3·68–7·35] to 4·08 [2·76–5·49]), high-middle SDI quintile (8·65 [5·88–11·82] to 7·40 [5·15–10·14]), middle SDI quintile (8·65 [5·85–12·13] to 7·26 [5·00–9·96]), and low SDI quintile (11·90 [8·19–16·58] to 10·08 [6·91–13·86]; figure 3B; appendix 1 pp 79–80).

Appendix 1 (p 49) illustrates the negative correlation between prevalence rates, DALY rates, and the HAQ Index. Specifically, the analysis reveals a negative correlation between prevalence rates and the HAQ Index (r –0·217, p<0·001). Additionally, DALY rates are negatively correlated with the HAQ Index (r –0·179, p=0·011).

### Burden of vision impairment due to AMD attributable to tobacco

In 2021, the total number of DALYs attributable to tobacco was 0·06 million (95% UI 0**·**03–0·10). Males were estimated to have 0·05 million (0·03–0·07) DALYs attributable to tobacco, or 18·72% (95% UI 11·69–26·02) of all AMD DALYs in males, whereas females were estimated to have 0·01 million (0·01–0·02) DALYs attributable to tobacco, or 3·89% (95% UI 2·20–5·83) of all DALYs from AMD in females in 2021 (appendix 1 pp 76–79).

**Figure 4:**
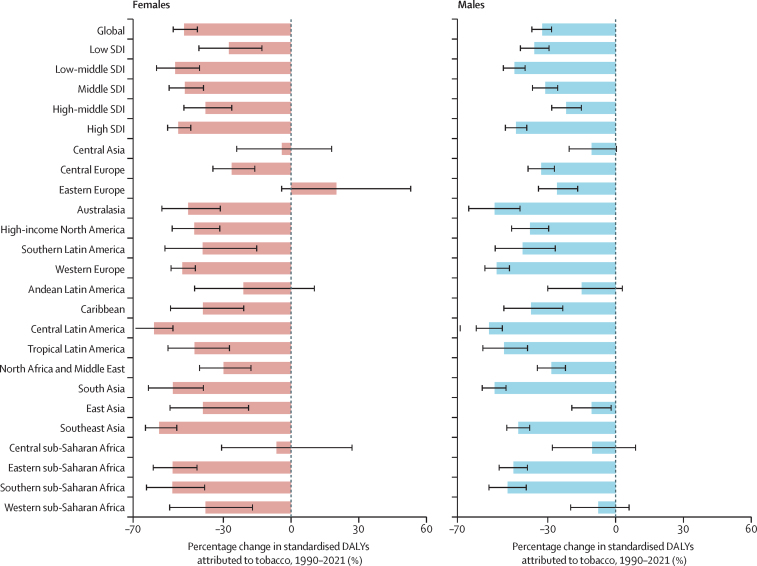
Percentage change in age-standardised DALYs attributed to tobacco as a risk factor from age-related macular degeneration by the SDI groups and regions, 1990–2021 Error bars indicate 95% uncertainty intervals. DALYs=disability-adjusted life-years. SDI=Socio-demographic Index.

A downward trend of attribution of tobacco to age-standardised DALYs is observed in all SDI levels. Globally, the percentages of DALYs attributable to tobacco decreased 20·03% from 12·45% (95% UI 7·73–17·37) in 1990 to 9·96% (6·12–14·06) in 2021. Specifically, the effect of tobacco on DALYs decreased across all SDI levels: 13·62% (95% UI 8·10–19·55) in 1990 to 10·00% (5·80–14·63) in 2021 for the high SDI quintile, 13·07% (8·14–17·97) to 11·81% (7·38–16·38) for the high-middle SDI quintile, 12·85% (8·14–17·57) to 10·03% (6·24–14·02) for the middle SDI quintile, 11·92% (7·40–16·44) to 9·24% (5·67–13·17) for the low-middle SDI quintile, and 7·84% (4·74–10·96) to 6·01% (3·56–8.60) for the low SDI quintile (figure 4; appendix 1 pp 76–79).

### Forecasting the burden of vision impairment due to AMD until 2050

**Figure 5:**
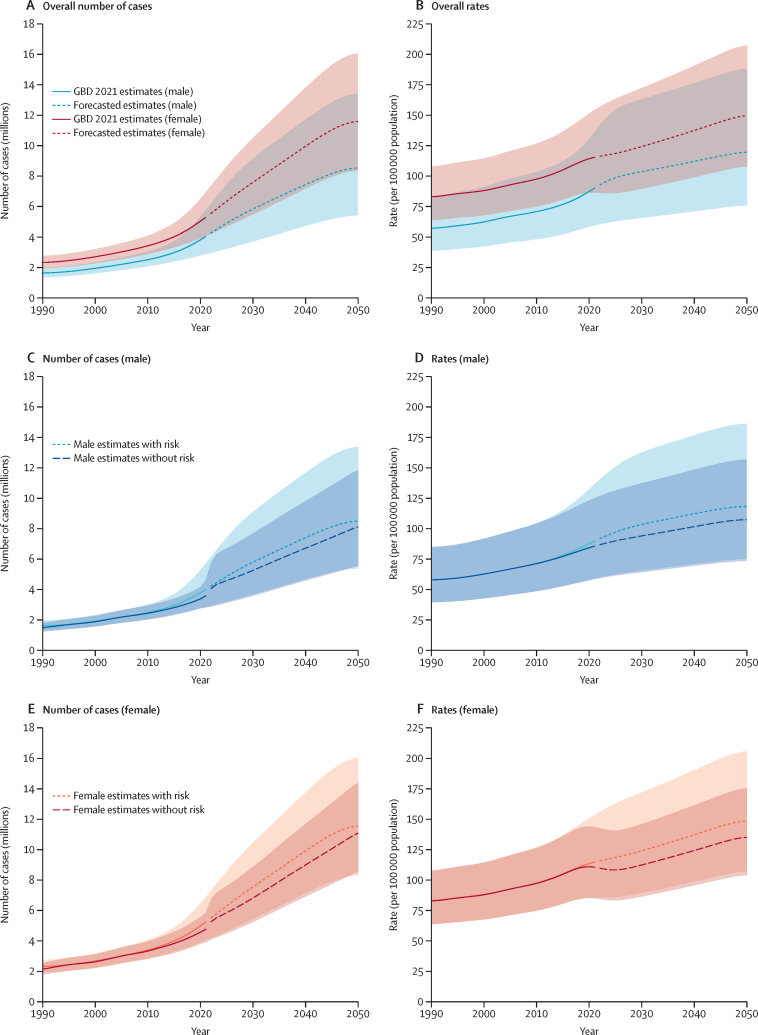
Forecasted prevalent cases and rates per 100 000 of the population of age-related macular degeneration by sex, comparing estimates with and without risk factor, all ages, 1990–2050 Number of cases and rates overall (A,B), for males (C,D), and for females (E,F). The shaded regions represent 95% uncertainty intervals, while the dashed line marks the forecast period beginning in 2022. The risk factor for this disease in this study is smoking.

The forecasted burden of vision impairment due to AMD is presented in figure 5 and appendix 1 (pp 80–83). In 2021, the number of individuals affected was estimated at 3·40 million males (95% UI 2·81–4·17) and 4·66 million females (3·88–5·65). These numbers are projected to increase substantially in the coming decades. By 2030, the burden is expected to rise to 5·82 million for males (3·69–9·16) and 7·54 for million females (5·44–10·46); the burden is forecasted to increase again to reach 7·43 million males (4·71–11·69) and 9·95 million females (7·17–13·80) by 2040. By 2050, the burden is projected to increase further to 9·02 million males (5·72–14·20) and 12·32 million females (8·88–17·08; figure 5A, B; appendix 1 pp 80–83) to a total of 21·34 million cases globally.

If the risk of tobacco use is eliminated, a substantial reduction in the burden of vision impairment due to AMD is expected for both sexes, with an overall reduction in 2050 of approximately 19·32 million. For males, the burden is projected to decrease as follows: in 2030, from 5·82 million (95% UI 3·69–9·16) to 5·27 million (3·61–7·70); in 2040, from 7·43 million (4·71–11·69) to 6·73 million (4·60–9·82); and in 2050, from 9·02 million (5·72–14·20) to 8·17 million (5·59–11·92; figure 5C, D; appendix 1 pp 80–83). For females, similar reductions are expected: in 2030, from 7·54 million (95% UI 5·44–10·46) to 6·84 million (5·26–8·88); in 2040, from 9·95 million (7·17–13·80) to 9·01 million (6·94–11·71); and in 2050, from 12·32 million (8·88–17·08) to 11·15 million (8·58–14·48; figure 5E, F; appendix 1 pp 80–83). Corresponding rates per 100 000 of the population also display decreases when tobacco use is eliminated (appendix 1 pp 80–83).

## Discussion

### Key findings

This study provides a comprehensive assessment of the global, regional, and national burden of vision impairment due to AMD, using estimates from GBD 2021. The total numbers, prevalence, and DALYs have increased since 1990, with a particularly notable rise in the east Asia region. This increase is probably driven by population growth and ageing, considering that age-standardised prevalence and DALY rates have shown a downward trend (–5·53% and –19·09%, respectively) compared with 1990. Subgroup analysis revealed that females consistently had higher rates than males for all measures across all age groups. A negative correlation was observed between HAQ Index and prevalence and DALY rates, with higher SDI quintiles associated with lower rates. This relationship underscores the crucial role of health-care access and quality in mitigating the burden of AMD, and highlights disparities in health-care resources as a major contributor to the unequal global burden. Furthermore, a consistent downward trend in age-standardised prevalence and DALY rates has been observed across all SDI levels from 1990 to 2021. This decline might be partly explained by the reduction of DALYs attributable to tobacco use by 20·03% globally over the same period. Lastly, projections of AMD-related vision impairment through to 2050 indicate a continuous increase in its global burden. However, the forecasts also highlight the potential for substantial reductions through effective tobacco regulation, particularly in lower SDI regions.

### Plausible underlying mechanism

AMD is a multifactorial disease characterised by a complex interplay between ageing, environmental risk factors, and genetic susceptibility.^20^ Among these factors, ageing is the most prominent risk factor.^21^, ^22^ A discernible increase in prevalence rates with advancing age was observed, in line with the findings from Wong and colleagues’ 2014 systemic review and meta-analysis that projected burden of AMD up to 2040.^7^

Distinctive findings in this study, compared to previous research and meta-analyses, include differences in the number of individuals affected by AMD. Unlike the review by Wong and colleagues, which estimated the total number of AMD cases without focusing on individuals with vision impairment,^7^ this study specifically examined individuals with vision impairment due to AMD. Consequently, while Wong and colleagues estimated approximately 196 million people would be affected by AMD globally in 2020,^7^ this study estimates approximately 8 million individuals with vision impairment caused by AMD in 2021. Recent data from the USA, which estimated that 1·49 million out of 18·34 million patients with AMD have vision-threatening late stage, correspond closely to this study's finding.^23^

Additionally, females exhibited higher prevalence and DALY rates than males in this study. There are reports indicating that hormonal differences might be associated with the progression of AMD.^24^ Nevertheless, there is ongoing debate regarding the role of oestrogen, with some studies suggesting that oestrogen could have a protective effect against AMD due to its antioxidative and anti-inflammatory signalling cascades.^25^, ^26^ There are reports indicating the onset of exudative AMD is associated with the use of either endogenous or exogenous oestrogen.^27^ Consequently, further investigation is necessary to conclusively clarify the effects of oestrogen on AMD.

An analysis of age-standardised DALY rates spanning the period from 1990 to 2021 revealed a consistent decline across all countries, excluding countries in central and southern sub-Saharan Africa. Additionally, a discernible global downward trend in age-standardised DALY rates was evident in all SDI quintiles. This reduction in DALY rates can be attributed to several factors. First, the implementation of the WHO Vision 2020 Act,^28^ which mandates the training of adequate numbers of eye care providers at all levels and the establishment of essential infrastructure and technology, has probably played a pivotal role.^29^ Vision 2020 has been instrumental in advocacy efforts by securing sufficient resources and the commitment to achieve its objectives.^29^, ^30^ Additionally, the emergence of novel medical resources such as anti-vascular endothelial growth factor (anti-VEGF) agents (eg, ranibizumab, aflibercept, faricimab, brolucizumab, and bevacizumab)^9^, ^17^ and ongoing research on treatments for non-exudative AMD, particularly geographic atrophy (eg, pegcetacoplan and avacincaptad pegol),^31^ might have contributed to the decline in DALY rates. Furthermore, advancements in diagnostic technologies, particularly the widespread adoption of optical coherence tomography for both detection and treatment monitoring, have significantly improved accuracy in diagnosis and treatment selection.^9^ However, given the substantial cost burden of anti-VEGF and disparities in medical resource allocation across different SDI quintiles, contribution of optical coherence tomography to the decrease in DALYs should not be overemphasised.^30^, ^32^ The global decline in tobacco use might have a more important role in decreasing age-standardised DALY rates across SDI quintiles.^33^ Furthermore, improvements in HAQ Index across all SDI quintiles,^30^ along with the heightened attention to personal health and education levels, probably contributed to the observed decline in age-standardised DALY rates.^34^

### Clinical and policy implications

Ageing is intrinsic and an unmodifiable element, yet is the strongest risk factor in AMD.^35^ Among modifiable risk factors, tobacco use is the most established, with previous research indicating a global three-fold increase in risk of AMD due to smoking.^9^ Males were more significantly affected by tobacco than females across all regions in 2021 (18·72% [95% UI 11·69–26·02] for males *vs* 3·89% [2·20–5·83] for females). This disparity could be attributed to the substantial difference in smoking prevalence between sexes, with male prevalence notably higher in 2015 (25·0% in males *vs* 5·4% in females).^33^ Surprisingly, a consistent decrease in the effect of tobacco on DALYs from 1990 to 2021 was observed across all SDI levels, potentially due to global efforts such as the implementation of the WHO Framework Convention on Tobacco Control, which have led to a decline in tobacco prevalence worldwide.^36^ The effect of tobacco on DALYs in the low-middle SDI quintile decreased notably in 2021 from 1990, with the reduction in age-standardised DALY rates being the highest, at 33·21%. This implies that in countries with limited resources, such as medical facilities, reducing the influence of tobacco could lead to a decrease in DALYs. Indeed, evidence suggests that strengthening tobacco control measures, including taxation policies, could further reduce prevalence.^36^ Targeting the reduction of modifiable risk factors is an essential strategy for preventing AMD. Thus, considering such evidence could be pivotal in future efforts to address AMD, particularly for lower SDI quintiles.

Although anti-VEGF treatments are known to be effective against the exudative form of AMD,^37^ given that the majority of patients with AMD have the non-exudative form, it is essential to develop treatments and preventive measures specifically targeting non-exudative AMD.^17^ As evidenced by the negative correlation between the HAQ Index and both prevalence and DALY rates, increased health-care resources are associated with a reduced burden of vision impairment due to AMD. However, access to costly treatments such as anti-VEGF therapies could be less accessible in low-income countries.^32^ Additionally, the progression of AMD from early to late stages and the implementation of visual restoration strategies, such as retinal pigment epithelium allograft transplantation, might also vary based on the level of medical resources in each country.^9^ To address the disproportionate burden of AMD globally, research on cost-effective treatment methods tailored to different SDI levels is essential. For instance, exploring the use of off-label bevacizumab might help reduce costs.^34^ Additionally, a high dietary intake of lutein and zeaxanthin might help reduce the risk of late AMD in lower SDI quintiles.^10^ Furthermore, prioritising service development for vulnerable groups in LMICs and implementing strategies to slow AMD progression could substantially reduce the global burden of AMD,^9^ and future studies should emphasise this focus.

### Strengths and limitations

Although our study benefited from a robust dataset comprised of national and regional levels, the present study is subject to several notable limitations. First, as this study used data from the GBD 2021, low data availability from certain countries, regions, and years cannot be overlooked. Consequently, due to this scarcity of data, descriptive statistics necessitated reliance on predictive modelling using covariates, potentially leading to less precise inferences compared with those for regions with more robust data. Second, an important limitation of our study is the absence of fundus photography data in certain regions, which are typically used for diagnosing AMD.^10^ This deficiency limits our understanding of whether the exudative or non-exudative form of AMD is responsible for the vision impairment. The availability of such data would facilitate distinguishing between these forms, allowing us to estimate the effect of anti-VEGF treatments on reducing DALYs. Moreover, analysing the effects of non-exudative AMD separately could provide valuable insights on the burden of vision impairment due to AMD, considering the limited treatment options and their potentially large influence on DALYs.^17^ Third, while this study provides data on the prevalence and DALYs of vision impairment due to AMD, there is a possibility that the burden of vision impairment due to AMD may have been underestimated, owing to the heterogeneous nature of AMD diagnostic classification. Fourth, the risk analysis focused solely on smoking as a risk factor, overlooking emerging risk factors such as low physical activity and lipid profiles.^9^ Incorporating various risk factors could have enabled the assessment of each factor's contribution to DALYs, thereby aiding addressing AMD on a global scale. Lastly, the effect of genetic susceptibility on AMD was not investigated, particularly regarding ethnicity. While prevalence rates tend to be higher among groups of European ancestry,^7^ our study, constrained by geographical data alone, did not reflect this aspect.

Despite these limitations, this study offers several strengths and novel contributions to the literature. Unlike previous global estimation studies,^7^ our study used robust GBD data to analyse both prevalence and DALYs, providing a more comprehensive understanding of AMD's burden. AMD, as a lifelong disability, has become an increasingly important public health concern due to increased longevity, contributing significantly to vision impairment, particularly in high-income countries.^2^ Thus, investigating DALYs rather than cross-sectional prevalence offers a more meaningful assessment of the effect of AMD on population health over time and informs strategies to mitigate the burden. Additionally, this study analysed the role of tobacco as a modifiable risk factor,^9^ quantifying the use of tobacco's contribution to DALYs across different SDI levels and observing a clear downward trend. These findings underscore the importance of addressing tobacco use to reduce AMD's global burden effectively. Furthermore, by forecasting the burden of vision impairment due to AMD through to 2050, and comparing scenarios with and without the effect of tobacco, our analysis highlights the potential for significant reductions in AMD-related vision impairment through targeted tobacco regulation.

## Conclusion

Vision impairment due to AMD remains a substantial global burden, affecting approximately 8 million individuals in 2021, with projections indicating an increase to 21·34 million by 2050. This study highlights the role of improved health-care access and reduced tobacco impact in lowering age-standardised DALY rates, particularly in lower SDI quintiles where resources are limited. Introducing cost-effective treatments and diagnostics, especially in LMICs, alongside tobacco regulation, could significantly reduce the global burden of AMD-related vision impairment.

### GBD 2021 Global AMD Collaborators

### Affiliations

### Contributors

### Data sharing

The findings from this study were produced using data available in public online repositories or in the published literature, data that are publicly available on request from the data provider, and data that are not publicly available due to restrictions by the data provider and which were used under licence for the current study. Details on data sources can be found on the GHDx website, including information about the data provider and links to where the data can be accessed or requested (where available). To download the data used in these analyses, please visit the Global Health Data Exchange GBD 2021 website.

## Declaration of interests

S Afzal reports support for the present manuscript from King Edward Medical University (Lahore, Pakistan); payment or honoraria for lectures, presentations, speakers bureaus, manuscript writing or educational events from King Edward Medical University and collaborative partners, including Johns Hopkins University (Baltimore, MD, USA), University of California (CA, USA), University of Massachusetts (MA, USA), King Edward Medical College Alumni Association of North America and the UK (KEMCAANA and KEMCA-UK), international scientific conferences, webinars and meetings; support for attending meetings, travel, or both from King Edward Medical University; participation on a Data Safety Monitoring Board or Advisory Board with the National Bioethics Committee Pakistan, King Edward Medical University Ethical Review Board, and Ethical Review Board Fatima Jinnah Medical University and Sir Ganga Ram Hospital; leadership or fiduciary role in other board, society, committee or advocacy group, paid or unpaid as a member of the Pakistan Association of Medical Editors, Fellow of Faculty of Public Health Royal Colleges UK (FFPH), Society of Prevention, Advocacy And Research, King Edward Medical University (SPARK), member Pakistan Society of Infectious Diseases, and member of the Technical Expert Advisory Group of the Government to formulate guidelines on the prevention, surveillance and research on infectious diseases; other financial or non-financial interests as Dean of Public Health and Preventive Medicine at King Edward Medical University, Chief Editor Annals of King Edward Medical University since 2014, Director Quality Enhancement Cell King Edward Medical University, at international level, Fellow of Faculty of Public Health UK, Advisory Board Member and Chair Scientific Session, KEMCA-UK, Chairperson International Scientific Conference, KEMCAANA, at national level, member of the Research and Publications Higher Education Commission, HEC (Pakistan), member of the Research and Journals Committee Pakistan Medical and Dental Council (Pakistan), member of the National Bioethics Committee (Pakistan), at Punjab level Member of the Corona Experts Advisory Group, member of the Technical Working Group for Infectious Diseases, member of the Dengue Experts Advisory Group, and Chair, Punjab Residency Program Research Committee; outside the submitted work. S Bhaskar reports grants or contracts from the Japan Society for the Promotion of Science (JSPS), Japanese Ministry of Education, Culture, Sports, Science and Technology (MEXT) and from The Australian Academy of Science; leadership or fiduciary roles in board, society, committee or advocacy groups, paid or unpaid as the visiting director in the department of neurology at the National Cerebral and Cardiovascular Center, Suita (Osaka, Japan), district chair of diversity, equity and inclusion at the Rotary District 9675, chair and manager of the Global Health and Migration Hub Community (Berlin, Germany), an editorial member of PLOS One, BMC Neurology, Frontiers in Neurology, Frontiers in Stroke, Frontiers in Aging, Frontiers in Public Health & BMC Medical Research Methodology, a member of the College of Reviewers (Canadian Institutes of Health Research, Government of Canada), a member of the scientific review committee at Cardiff University Biobank (UK), an expert advisor and reviewer with the Cariplo Foundation (Milan, Italy), a Visiting Director at the National Cerebral and Cardiovascular Center, Department of Neurology, Division of Cerebrovascular Medicine and Neurology (Suita, Osaka, Japan); outside the submitted work. R K Garg reports royalties or licences from MedLink Neurology and UpToDate; outside the submitted work. I Ilic reports support for the present manuscript from the Ministry of Education, Science and Technological development, Republic of Serbia (project No 175042, 2011–2023). M Ilic reports support for the present manuscript from the Ministry of Education, Science and Technological development, Republic of Serbia (number 451–03–47/2023–01/200111). K Krishan reports non-financial support from the UGC Centre of Advanced Study, CAS II, awarded to the Department of Anthropology, Panjab University (Chandigarh, India); outside the submitted work. M Lee reports support for the present manuscript from the Ministry of Education of the Republic of Korea and the National Research Foundation of Korea (NRF-2023S1A3A2A05095298). B Oancea reports grants or contracts from MRID, Project PNRR-I8 no 842027778, contract no 760096; outside the submitted work. R Passera reports participation on a Data Safety Monitoring Board or Advisory Board as a member of the Data Safety Monitoring Board of the clinical trial “Consolidation with ADCT-402 (loncastuximab tesirine) after immunochemotherapy: a phase II study in BTKi-treated/ineligible Relapse/Refractory Mantle Cell Lymphoma (MCL) patients” - FIL, Fondazione Italiana Linfomi, Alessandria; leadership or fiduciary role in other board, society, committee or advocacy group, paid or unpaid as a member of the EBMT Statistical Committee, European Society for Blood and Marrow Transplantation, Paris, France, and as a past member 2020–2023 (biostatistician) of the IRB/IEC Comitato Etico AO SS. Antonio e Biagio Alessandria-ASL AL-VC (Venice, Italy); outside the submitted work. Y L Samodra reports a leadership or fiduciary role as a co-founder of Benang Merah Research Center (Indonesia); outside the submitted work. V Sharma reports support from DFSS (MHA's) research project (DFSS28(1)2019/EMR/6) at Institute of Forensic Science & Criminology, Panjab University (Chandigarh, India); outside the submitted work. J I Shin reports support from the Yonsei Fellowship, funded by Lee Youn Jae. J A Singh reports consulting fees from ROMTech, Atheneum, Clearview healthcare partners, American College of Rheumatology, Yale, Hulio, Horizon Pharmaceuticals, DINORA, ANI/Exeltis, USA, Frictionless Solutions, Schipher, Crealta/Horizon, Medisys, Fidia, PK Med, Two labs., Adept Field Solutions, Clinical Care options, Putnam associates, Focus forward, Navigant consulting, Spherix, MedIQ, Jupiter Life Science, UBM LLC, Trio Health, Medscape, WebMD, and Practice Point communications; and the National Institutes of Health]; payment of honoraria for lectures, presentations, speakers bureaus, manuscript writing or education events as a member of the speaker's bureau of Simply Speaking; support for attending meetings as a past steering committee member of OMERACT; participation on a Data Safety Monitoring Board or Advisory Board with the FDA Arthritis Advisory Committee; Leadership or fiduciary role in other board, society, committee or advocacy group, paid as a past steering committee member of the OMERACT (an international organisation that develops measures for clinical trials and receives arm's length funding from 12 pharmaceutical companies), unpaid as a Co-Chair of the Veterans Affairs Rheumatology Field Advisory Committee, and unpaid as an editor and Director of the UAB Cochrane Musculoskeletal Group Satellite Center on Network Meta-analysis; stock or stock options in Atai life sciences, Kintara therapeutics, Intelligent Biosolutions, Acumen pharmaceutical, TPT Global Tech, Vaxart pharmaceuticals, Atyu biopharma, Adaptimmune Therapeutics, GeoVax Labs, Pieris Pharmaceuticals, Enzolytics, Seres Therapeutics, Tonix Pharmaceuticals Holding Corp., and Charlotte's Web Holdings, and previous stock options in Amarin, Viking, and Moderna Pharmaceuticals; outside the submitted work. J H V Ticoalu reports leadership or fiduciary roles as the co-founder of Benang Merah Research Center (Indonesia); outside the submitted work. M Zielinska reports other financial or non-financial interests as an Alexion, AstraZeneca Rare Disease employee; outside the submitted work. All other members declare no competing interests.
