# Supplementary material for: Global burden of vision impairment due to age-related macular degeneration, 1990–2021, with forecasts to 2050: a systematic analysis for the Global Burden of Disease Study 2021
Source: Lancet Glob Health. 2025 Jun 25;13(7):e1175–90. doi: 10.1016/S2214-109X(25)00143-3 (PMC12208786; doi:10.1016/S2214-109X(25)00143-3)
Supplement: Supplementary appendix 1 [file mmc1.pdf]

THE LANCET
Global Health

Supplementary appendix 1

This appendix formed part of the original submission and has been peer reviewed. We post it as supplied by the authors.

Supplement to: GBD 2021 Global AMD Collaborators. Global burden of vision impairment due to age-related macular degeneration, 1990–2021, with forecasts to 2050: a systematic analysis for the Global Burden of Disease Study 2021. *Lancet Glob Health* 2025; **13:** e1175–90.

Appendix 1

Table of contents

Section 1: GBD OVERVIEW....5

Section 1.1: Geographic locations of the analysis....5

Section 1.2: GBD cause list....6

Section 1.3: AMD methodology overview....6

Section 2: Data identification and preparation....9

Section 2.1: Case definition....9

Section 2.2: Data input....11

Section 2.2.1: Input data....11

Section 2.3: Data preparation and processing....13

Section 2.3.1: data preparation and processing....13

Section 2.3.2: MR-BRT meta-regression modelling....14

Section 2.3.3: Crosswalking....16

Section 2.3.4: Age sex splitting....16

Section 3: Disease Modelling Meta-Regression 2.1 modeling....17

Section 3.1: DisMod-MR 2.1 estimates....17

Section 3.1.1: Estimation of sequelae and causes....17

Section 3.1.2: DisMod-MR 2.1 description....17

Section 4: Modelling and post processing....20

Section 4.1: Modelling and post processing....20

Section 4.1.1: impairment and underlying squeeze....20

Section 4.1.2: Disability weights....21

Section 4.1.3: GBD 2013 European disability weights measurement study....21

Section 4.1.4: Comorbidity correction (COMO)....23

Section 4.1.5: YLD computation....24

Section 5: Forecasting for 2050 estimates....25

Section 5.1: Overview of the forecasting framework 25
Section 5.2: Forecasting non-fatal disease burden 25
Section 5.2.1: Prevalence-only models 26
Section 5.3: Ensemble modelling 26
Section 5.3.1: Ensemble modelling framework 26
Section 5.3.2: ARC model 28
Section 5.3.3: MR-BRT model 29
Section 5.4: Computing SEVs, PAFs, and scalars 30
Section 5.4.1: Risk exposure (summary exposure values) 30
Section 5.4.2: Forecasting direct smoking exposure 30
Section 5.4.3: Scalars 31
Section 5.5: Uncertainty interval estimation 31
Section 5.6: Ensemble model construction 32
Section 5.6.1: Root mean squared error (RMSE) 32
Section 5.7: Post-processing of the results of align with GBD 2021 33
Section 6: Risk factor 35
Section 6.1: Overview of risk factor 35
Section 6.2: Smoking 36
Section 6.2.1: Smoking flowchart 36
Section 6.2.2: Exposure and SEV 36
Section 6.2.3: Relative risk 38
Section 7: Healthcare Access and Quality Index 42
Section 7.1: Overview 42
Section 7.2: Utilizing HAQ index 43
Section 7.2.1: linear interpolation & extrapolation 43
Section 7.2.2: Age groupings 44
References 46

List of appendix figures and tables

Appendix figures **47**

Figure S1. Vision impairment due to age-macular degeneration by constituent sequelae prevalence for both sexes combined, 1990–2021. (A) Total number of moderate vision loss, severe vision loss, and blindness. (B) Rates (per 100,000 population) of age-standardized and all-age moderate vision loss, severe vision loss, and blindness. 47

Figure S2. Vision impairment due to age-macular degeneration by constituent sequelae YLDs for both sexes combined, 1990–2021. (A) Total number of moderate vision loss, severe vision loss, and blindness. (B) Rates (per 100,000 population) of age-standardized and all-age moderate vision loss, severe vision loss, and blindness. 48

Figure S3. Association between Healthcare Access and Quality Index and age-standardized rate from age-related macular degeneration by the SDI groups, 2021. (A) Prevalence rate and (B) DALYs rate.. 49

Appendix tables **50**

Table S1. Checklist of information that should be included in new reports of glob al health estimates for GBD 2021 50

Table S2. Global count (million) and percentage change of prevalence for age-related macular degeneration from 1990 to 2021 52

Table S3. Global count (thousands) and annual percentage change of DALYs for age-related macular degeneration from 1990 to 2021 54

Table S4. Global count and rates (per 100,000 population) of prevalence for age-related macular degeneration at the global level by sex, from 1990 to 2021 56

Table S5. Global count and rates (per 100,000 population) of DALYs for age-related macular degeneration at the global level by sex, from 1990 to 2021 59

Table S6. Global count and rates (per 100,000 population) of prevalence in three-categorized vision impairment due to age-macular degeneration from 1990 to 2021 61

Table S7. Global count and rates (per 100,000 population) of YLDs in three-categorized vision impairment due to age-macular degeneration from 1990 to 2021 64

Table S8. Global count and rates (per 100,000 population) of prevalence from age-related macular degeneration by age group and sex, 2021 66

Table S9. Global count and rates (per 100,000 population) of DALYs from age-related macular degeneration by age group and sex, 2021 67

Table S10. Total count of prevalent cases (million) for age-related macular degeneration for both sexes at the Socio-demographic Index, 1990–2021 68

Table S11. Total count of DALYs case(thousand) for age-related macular degeneration for both sexes at the Socio-demographic Index, 1990–2021 70

Table S12. Total age-standardized rate (per 100,000 population) of prevalence for age-related macular degeneration for both sexes at the Socio-demographic Index, 1990–2021 72

Table S13. Total age-standardized rate (per 100,000 population) of DALYs for age-related macular degeneration for both sexes at the Socio-demographic Index, 1990–2021......... 74

Table S14. Total counts of DALYs attributed to risk factor (tobacco) of age-related macular degeneration by region and sex, 1990 and 2021 76

Table S15. Age-standardized percentage and percentage change of DALYs attributed to risk factor (tobacco) of age-related macular degeneration by region and sex, 1990–2021 78

Table S16. Forecasted prevalent cases and rates (per 100,000 population) of age-related macular degeneration by sex, comparing estimates with risk factor, all ages, 1990–2050 80

Table S17. Forecasted prevalent cases and rates (per 100,000 population) of age-related macular degeneration by sex, comparing estimates without risk factor, all ages, 1990–2050 82

END...84

Section 1: GBD OVERVIEW

Section 1.1: Geographic locations of the analysis

We developed estimates for 204 countries and territories, organized into 21 regions and seven broad super-regions. These super-regions are: Central Europe, Eastern Europe, and Central Asia; High-Income; Latin America and the Caribbean; North Africa and the Middle East; South Asia; Southeast Asia, East Asia, and Oceania; and Sub-Saharan Africa. In GBD 2021, we continue subnational analyses for countries previously included, such as Brazil, China, Ethiopia, India, Indonesia, Iran, Italy, Japan, Kenya, Mexico, New Zealand, Nigeria, Norway, Pakistan, Russia, the Philippines, Poland, South Africa, Sweden, the UK, and the USA. These analyses are conducted at the first administrative level within each country, except for New Zealand (by Māori ethnicity), Sweden (by Stockholm and non-Stockholm areas), the UK (by local government authorities), and the Philippines (by provinces).

To comply with data usage requirements, we present subnational estimates for Brazil, India, Indonesia, Japan, Kenya, Mexico, Sweden, the UK, and the USA in this document. Subnational estimates for China are depicted in maps. Estimates for other countries will be released in separate publications. At the most detailed spatial resolution, we generated estimates for 983 unique locations. Similar to Global Burden of Disease Study (GBD) 2019, GBD 2021 employs a classification of standard and non-standard locations. Standard GBD locations include all subnational from countries with high-quality data and populations exceeding 200 million, along with all other countries. This classification encompasses subnational for China, India, the USA, and Brazil, but excludes Indonesia; data for China, India, the USA, and Brazil are also available at the national level. All other countries with subnational estimates are defined as non-standard locations.

Section 1.2: GBD cause list

The GBD cause and sequelae list is structured hierarchically to address the varied needs of its users. At Level 1, there are three main cause groups: communicable diseases and nutritional conditions, non-communicable diseases, and injuries. These broad categories are further divided into 22 cause groupings at Level 2, providing greater specificity. Levels 3 and 4 offer the most detailed information on causes, with some specific causes categorized at Level 3 and others at Level 4, while Level 3 remains an aggregate category for certain causes. Beyond these, the sequelae of diseases and injuries are classified at Levels 5 and 6, where sequelae are defined as distinct and mutually exclusive health consequences directly attributable to a cause. Level 6 provides the finest granularity, with sequelae aggregated into summary categories at Level 5 for causes with numerous sequelae. The cause list evolves continuously to align with public health priorities and the guidance of the Scientific Council and GBD collaborators. For GBD 2021, the causes of death list increased to 288 causes (from 286 in GBD 2019), and the non-fatal cause list expanded to 365 causes (from 364 in GBD 2019). Overall, GBD 2021 encompasses 371 fatal and non-fatal causes.

In the GBD framework, age-related macular degeneration (AMD) is classified as a Level 3 cause within the broader category of vision loss (a Level 2 cause), which itself falls under the overarching category of non-communicable diseases at Level 1. AMD is also included in the non-fatal cause list, highlighting its significant contribution to morbidity rather than mortality. This classification underscores the impact of AMD as a leading cause of vision impairment, with substantial implications for quality of life and public health policy.

Section 1.3: AMD methodology overview

We estimated the prevalence and disability-adjusted life years (DALYs) associated with vision impairment due to AMD over the period 1990–2021 and projected these estimates for the

forecast period 2022–2050. The analysis encompassed data stratified by sex, age, and region, covering a total of 204 countries globally.

Flowchart

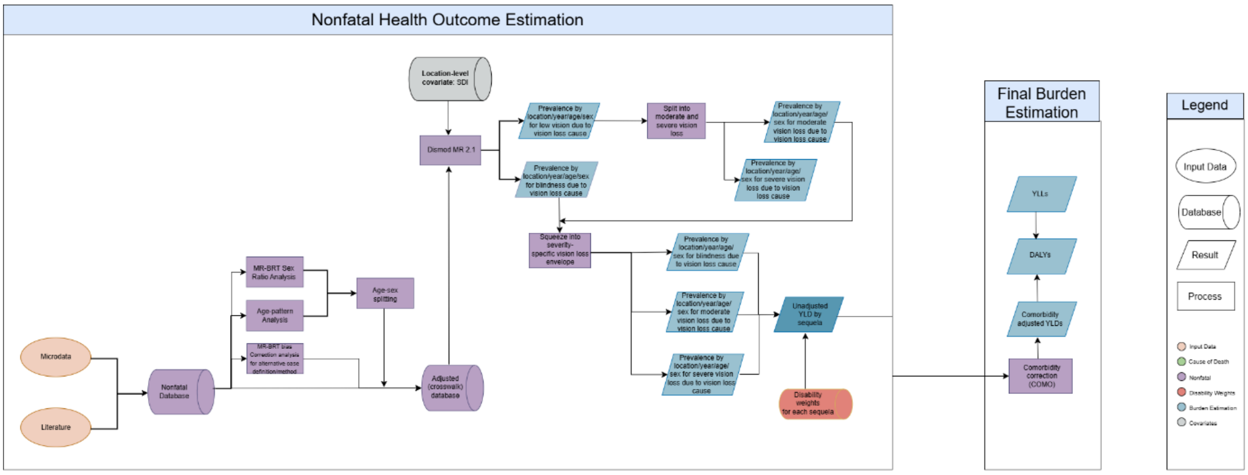

Our study followed an 8-step methodological framework to ensure comprehensive and robust estimates:

- Data identification and access
- Separation of raw data into datasets called "age-macular degeneration" for all-cause vision loss
- Studies only specifying "both" sex information split into male- and female-specific data points using MR-BRT
- Adjustment of non-reference data to the reference definition of presenting vision data that fit within the WHO severity categories
- Application of an age pattern to data with age groups greater than or equal to 45 years
- Dismod-MR 2.1 modeling

- Modeling and Post-Processing

- Extrapolation, Age-standardization & forecasting for 2022 and 2050 estimates

Section 2: Data identification and preparation

Section 2.1: Case definition

The classification of vision impairment follows the criteria defined by the International Classification of Diseases, 11th Edition (ICD-11), which categorizes vision loss into three levels of severity: Moderate Vision Loss, Severe Vision Loss, and Blindness. These categories are based on measured visual acuity using standardized tools such as the Snellen Chart or LogMAR Chart, ensuring consistency and accuracy. The thresholds for visual acuity, such as "≥6/60 and <6/18," correspond to the ICD-11 definitions for each severity level. Data collected using visual acuity tests are directly applied to these ICD-11 standards to classify the severity of vision impairment.

To summarize:

- ICD-11-based definitions:
  - The severity levels of vision impairment are defined according to ICD-11.
  - For instance, moderate vision loss is categorized as having a visual acuity of "≥6/60 and <6/18" for distance vision, as specified by ICD-11.
- Measurement Tools:
  - Visual acuity data are collected using standardized tools such as the Snellen Chart or LogMAR Chart.
  - These measured data are then mapped to the ICD-11 criteria to assign severity levels.

In summary, the severity of vision impairment is classified using ICD-11-defined thresholds, while the data for these classifications are derived from standardized visual acuity

measurements. This approach ensures consistency in assessing vision loss severity across studies and datasets (**Appendix Table A**).

Appendix Table A. Severity of vision loss assigned based on range of visual acuity for distance vision loss and presbyopia

Vison loss severity	Case definition
**Blindness**	Distance visual acuity of <3/60 or <10% visual field around central fixation
**Severe vision loss**	Distance visual acuity of ≥3/60 and <6/60
**Moderate vision loss**	Distance visual acuity of ≥6/60 and <6/18

We assess vision loss attributed to several causes, including uncorrected refractive error, cataract, glaucoma, macular degeneration, diabetic retinopathy, trachoma, vitamin A deficiency, retinopathy of prematurity, meningitis, encephalitis, onchocerciasis, and a residual category for other forms of vision loss. Vision loss related to vitamin A deficiency, retinopathy of prematurity, meningitis, encephalitis, and onchocerciasis is modeled as part of their respective underlying causes, as detailed in the relevant sections (**Appendix Table B**).

Appendix Table B. Causes of vision loss

Condition	Case definition
**Cataract**	Clouding of the lens of the eye due to protein buildup that impairs vision. Cataracts can be addressed via surgical lens replacement.
**Diabetic retinopathy**	Damage to the retina caused by damaged blood vessels that can leak blood into the retina and cause scarring of the retina.
**Glaucoma**	A condition with increased intraocular pressure which can lead to damage of the optic nerve.
**Age-related macular**	**Age-related deterioration of the macula, the part of**

**degeneration**	**the retina responsible for central vision, leading to central vision loss.**
**Uncorrected refractive error**	Blurry vision due to the lens's inability to focus. The blurriness caused by refractive error can be addressed through the use of contact lenses, glasses, or refractive surgery. Uncorrected refractive error is the difference in acuity between presenting vision (whatever corrective lens the individual uses) and best corrected vision.
**Trachoma**	Results from a conjunctival bacterial infection (Chlamydia trachomatis) that produces inflammation and inversion of the eyelids and eyelashes scratching and scarring the cornea, and eventually leading to trichiasis and impaired vision from corneal scarring.

Section 2.2: Data input

Section 2.2.1: Input data

The preparation of data for this analysis was carried out through a systematic review conducted by the Vision Loss Expert Group (VLEG), focusing on population-based studies of vision impairment and blindness. This review was supplemented by grey literature sources to ensure comprehensive data inclusion. Eligible studies were integrated with additional data sources, including the Rapid Assessment of Avoidable Blindness (RAAB) studies, the US National Health and Nutrition Examination Survey (NHANES), and the WHO Study on Global Ageing and Adult Health (SAGE). The data preparation process was supported by the GBD team to enhance the robustness of the analysis.

To perform a comprehensive review of the literature, the VLEG commissioned the York Health Economics Consortium in the UK. This effort involved systematic searches of major databases, including Embase, SciELO, MEDLINE, WHOLIS, and Open Grey, to identify relevant studies. Titles and abstracts were screened rigorously, and regional VLEG committees conducted detailed quality assessments to determine final inclusion eligibility. The methodology and search keywords used for this process are detailed in prior studies conducted

by the GBD team.

Additional data were drawn from RAAB surveys, with new five-year age-disaggregated data obtained from the RAAB repository. These surveys, which specifically target individuals aged 50 years and older, were critical for providing high-quality data in low- and middle-income countries (LMICs), where other data sources may be limited. Population-representative data, derived primarily from national and subnational cross-sectional surveys, were utilized to model cause-specific vision impairment. The methodology and implementation details for RAAB surveys, which play a pivotal role in capturing vision impairment in LMICs, have been described in prior publications. This integrated approach ensured a comprehensive and reliable analysis of global vision loss. The figure below illustrates the global distribution of RAAB and non-RAAB data sources used in this study (**Appendix Figure A-B***)*. For detailed information on the data, please refer to **Appendix 2**.

Appendix Figure A*. Global distribution of RAAB source for age-macular degeneration from 1990 to 2021*

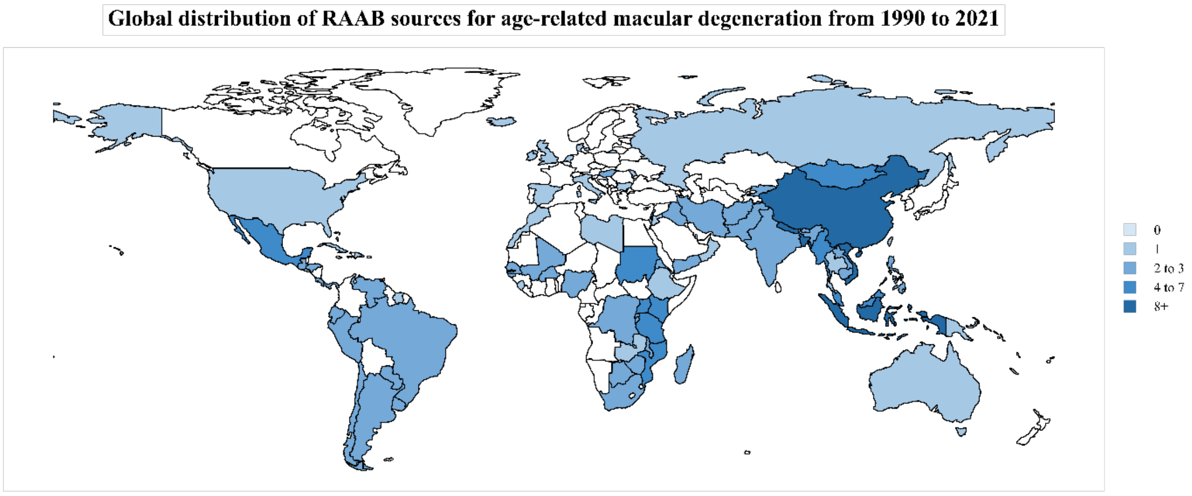

__Appendix Figure B__. Global distribution of non-RAAB source for age-macular degeneration from 1990 to 2021

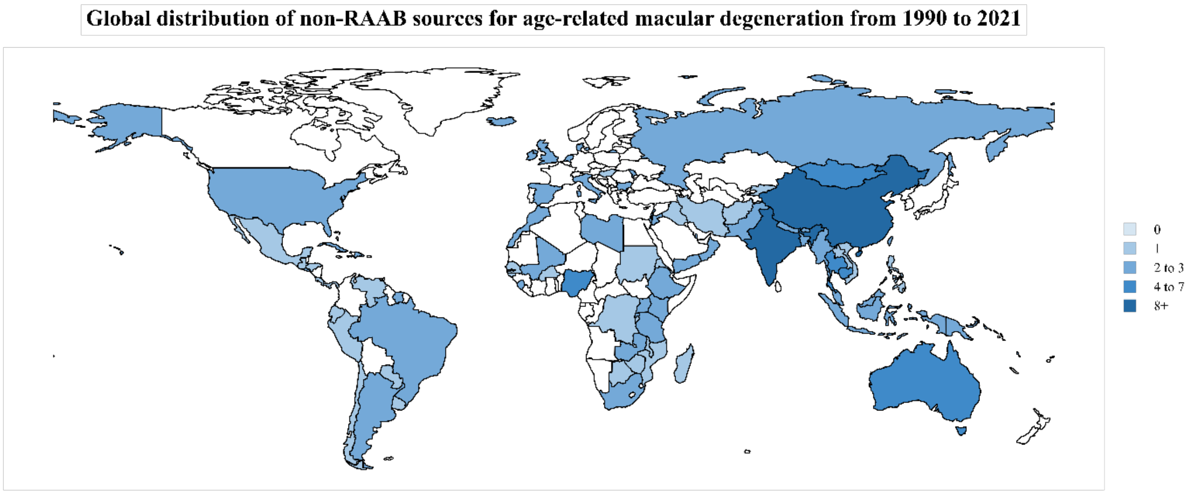

Section 2.3: Data preparation and processing

Section 2.3.1: data preparation and processing

Initially, raw data were organized into datasets containing age-macular degeneration "envelopes" for all-cause mild, moderate, and severe vision loss. For studies that reported combined male and female prevalence data, we disaggregated the data into male-specific and female-specific prevalence estimates. This was achieved by identifying within-study datapoints matched by age, year, and location that provided separate male and female prevalence data. The log ratio of female-to-male prevalence from these matched datapoints was then used as input for a mixed-effects meta-regression tool, MR-BRT (Meta-Regression–Bayesian, Regularized, Trimmed), developed by the Institute for Health Metrics and Evaluation (IHME). MR-BRT accounts for between-study heterogeneity in uncertainty adjustments and enables trimming of outlier datapoints. A detailed description of the MR-BRT methodology is available in previous publications. Using the results from this model and demographic population data by location, we estimated sex-specific prevalence rates of vision impairment.

Subsequently, MR-BRT was used to adjust non-reference data to align with the

reference definition of presenting vision, adhering to the WHO severity categories. This adjustment was applied to data from studies that did not use RAAB methods. For datasets reporting combined severity levels (e.g., moderate and severe vision loss or severe vision loss and blindness combined), we separated them into reference severity groups—moderate vision loss, severe vision loss, and blindness. This was achieved using a log-ratio meta-regression with a cubic spline on age and linear tails. The input data for this meta-regression consisted of studies that provided matched data by age, year, sex, and location for each severity category individually (e.g., moderate vision loss and severe vision loss reported separately). To ensure consistency across age ranges, any input data collected for age ranges exceeding 45 years were further split into five-year age bins, allowing for standardized comparisons and modeling across datasets.

Section 2.3.2: MR-BRT meta-regression modelling

MR-BRT is a meta-regression modeling tool developed at IHME, designed to incorporate uncertainty in the dependent variable, which is essential when input data come from scientific studies reported with uncertainty. Observations with higher uncertainty are given less weight in the model. MR-BRT can include both fixed effects (binary and continuous covariates) and random effects (group-level variation), enhancing its capability to describe variation. A linear mixed-effects meta-regression in MR-BRT is expressed as:

$$y_{ij} = \beta_0 + \beta_1 x_1 + \cdots + \beta_n x_n + u_j + \epsilon_{ij}$$

Where y_{ij} is the observation value, β terms are the linear predictor including covariates, u_j is a random intercept for study j, and ϵ_{ij} is the stochastic error.

Key features of MR-BRT include:

● Bayesian priors: Incorporate external information with uniform or Gaussian priors.

● LASSO and ridge regression: Implement L1 (Laplace priors) and L2 (Gaussian priors) regularization.

● Trimming: Identifies and removes outliers during the model fitting process.

● Spline terms: Uses B-splines to model nonlinear effects of covariates, with control over flexibility.

● Ratio model: Handles pairs of exposure intervals, often used with a spline to capture nonlinear exposure effects.

In MR-BRT, a combination of fixed effects and random effects is utilized, enabling the model to account for both within-study and between-study variability. This flexible modeling approach allows for the integration of diverse datasets and produces more accurate and reliable estimates. Fixed effects are used to model covariates observed within studies, such as age, sex, location, and year. These effects are assumed to be consistent across all studies and directly quantify the influence of each covariate on the outcome variable. By capturing the direct impact of these covariates, fixed effects provide a clear understanding of their relationship with the outcome.

Random effects, on the other hand, are introduced to explain variability across studies. They account for the unique characteristics of each study and their potential influence on the results. Random effects address between-study heterogeneity, reflecting the distinct variability inherent in individual studies. This approach ensures that differences between studies are appropriately considered, enhancing the robustness of the model's estimates. The source code for MR-BRT is publicly available on GitHub as the Python package mrtool (ihmeuw-msca,

2023) The mrtool package builds upon the open source mixed effects package LimeTr (https://github.com/zhengp0/limetr).

Section 2.3.3: Crosswalking

Crosswalking involves adjusting data to accommodate known biases. An observation is deemed biased if it consistently deviates from the standard GBD definition of the modeled parameter. For instance, this might include disease incidence measures that are self-reported rather than diagnosed by a doctor, or diagnostic tests with lower sensitivity or specificity compared to the gold standard method. When the disparity between an alternative measurement method and the GBD definition is consistent and systematic, it can be modeled using covariates. This allows us to predict the required adjustment for a given alternative or non-standard observation. Through this process, GBD models can integrate data from a broader range of sources.

Section 2.3.4: Age sex splitting

Prior to modeling, we ran a DisMod-MR 2.1 model with age-disaggregated data to derive age patterns for countries. Subsequently, we employed these patterns to divide aggregated all-age data into the desired 5-year age groups for ST-GPR modeling. This process entailed computing a constant, denoted as "k," which represented the ratio of the all-age data point to the all-age estimated utilization rate.

$$k = \frac{\mu_{all\ age}}{\hat{\mu}}$$

The age-specific utilization rates were then multiplied by this constant, thereby integrating observation-specific and estimated age-pattern uncertainties into the final dataset. The segmented data were subsequently incorporated into the final DisMod-MR 2.1 model, $\hat{\mu}$.

Section 3: Disease Modelling Meta-Regression 2.1 modeling

Section 3.1: DisMod-MR 2.1 estimates

Section 3.1.1: Estimation of sequelae and causes

The Bayesian meta-regression method DisMod-MR 2.1 is the most commonly used estimation technique. However, for specific diseases like HIV/AIDS or measles, disease-specific natural history models are employed because the basic three-state model in DisMod-MR 2.1 (susceptible, cases, dead) cannot sufficiently capture the disease's complexity. For diseases with various severity levels of sequelae, such as COPD or diabetes mellitus, DisMod-MR 2.1 is used to meta-analyze overall prevalence data, while separate models are used to analyze the proportions of cases with different severity levels or sequelae. Similarly, DisMod-MR 2.1 is used to meta-analyze data on the proportions of liver cancer and cirrhosis attributed to underlying causes like hepatitis B, hepatitis C, and alcohol use disorders.

Section 3.1.2: DisMod-MR 2.1 description

In DisMod-MR 2.1, analysts have the option to choose between Gaussian, log-Gaussian, Laplace, or log-Laplace likelihood functions. The default equation for the data likelihood in log-Gaussian form is presented as follows:

$$-\log[p(y_j|\Phi)] = \log(\sqrt{2\pi}) + \log(\delta_j + s_j) + \frac{1}{2}\left(\frac{\log(a_j + \eta_j) - \log(m_j + \eta_j)}{\delta_j + s_j}\right)^2$$

Where:

y_j is a measurement value (ie, datapoint)

Φ denotes all model random variables

η_j is the offset value, eta, for a particular integrand (prevalence, incidence, remission, excess mortality rate, with-condition mortality rate, cause-specific mortality rate, relative risk, or standardised mortality ratio)

a_j is the adjusted measurement for datapoint j, defined by:

$$a_j = e^{(-u_j - c_j)} y_j$$

Where:

u_j is the total area effect (ie, the sum of the random effects at three levels of the cascade: super-region, region, and country)

c_j is the total covariate effect (ie, the mean combined fixed effects for sex, study-level, and country-level covariates), defined by:

$$c_j = \sum_{k=0}^{K[I(j)]-1} \beta_{I(j),k} \hat{X}_{k,j}$$

with standard deviation (SD)

$$s_j = \sum_{l=0}^{L[I(j)]-1} \zeta_{I(j),l} \hat{Z}_{k,j}$$

Where:

k denotes the mean value of each datapoint in relation to a covariate (also called x-covariate)

$I(j)$ denotes a datapoint for a particular integrand, j

$\beta_{I(j),k}$ is the multiplier of the kth x-covariate for the ith integrand

$\hat{X}_{k,j}$ is the covariate value corresponding to the datapoint j for covariate k

l denotes the SD of each datapoint in relation to a covariate (also called z-covariate)

$\zeta_{I(j),l}$ is the multiplier of the lth z-covariate for the ith integrand

δ_j is the SD for adjusted measurement j, defined by

$$\delta_j = \log[y_j + e^{(-u_j - c_j)} \eta_j + c_j] - \log[y_j + e^{(-u_j - c_j)} \eta_j]$$

Where mj denotes the model for the jth measurement, not counting effects or measurement noise and defined by:

$$m_j = \frac{1}{B(j) - A(j)} \int_{A(j)}^{B(j)} I_j(a) da$$

Where:

$A(j)$ is the lower bound of the age range for a datapoint j

$B(j)$ is the upper bound of the age range for a datapoint j

$I(j)$ denotes the function of age corresponding to the integrand for datapoint j

In GBD 2021, the estimation of non-fatal impairment due to macular degeneration was performed using the DisMod-MR 2.1 model. This model ensures consistency among all disease parameters by utilizing differential equations with appropriate boundary conditions. Additionally, it employs an offset log-normal model with fixed effects for location-specific covariates and random effects to account for variations across different locations.

Section 4: Modelling and post processing

Section 4.1: Modelling and post processing

Section 4.1.1: impairment and underlying squeeze

For the GBD 2021, consistent with previous iterations (GBD 2019, GBD 2017, and GBD 2016), we estimated the country-age-sex-year prevalence of nine specific impairments. These impairments represent conditions or domains of functional health loss associated with various GBD causes, where better data exists for estimating the overall prevalence of the impairment than for each underlying sequela. The impairments analyzed included anemia, epilepsy, hearing loss, heart failure, intellectual disability, infertility, vision loss, Guillain-Barré syndrome, and pelvic inflammatory disease. Prevalence estimates for overall impairments were derived using DisMod-MR 2.1, applying constraints on cause-specific estimates to ensure that the total prevalence for each impairment aligned with the sum of its constituent causes, such as the 19 causes related to blindness.

Severity levels for anemia, epilepsy, hearing loss, heart failure, and intellectual disability were estimated at varying degrees. For infertility, we differentiated between primary infertility (inability to conceive) and secondary infertility (difficulty conceiving again), and assessed the impact on both men and women. In the case of epilepsy, we analyzed proportions of idiopathic versus secondary epilepsy and categorized cases into severe and less severe forms using mixed effects regression models. Sparse data regarding the proportion of seizure-free individuals with treated epilepsy were aggregated using a random effects meta-analysis. DisMod-MR 2.1 also generated country-, age-, sex-, and year-specific severity levels for hearing and vision loss. Due to limited information on the severity of intellectual disability, we assumed a globally similar distribution of severity based on a random effects meta-analysis of IQ-specific data. This assumption was further informed by cause-specific severity distributions related to chromosomal abnormalities and iodine deficiency. The severity of intellectual disability

associated with long-term sequelae from conditions such as neonatal disorders, meningitis, encephalitis, neonatal tetanus, and malaria was estimated within combined health states that included multiple impairments, such as motor impairment.

Section 4.1.2: Disability weights

To calculate Years Lived with Disability (YLDs) for a specific health condition within a certain population, the prevalence of that condition is multiplied by a disability weights (DWs) which indicates the degree of health impairment linked to the condition. DWs are assessed on a scale from 0 to 1: 0 indicates a state equivalent to perfect health, while 1 represents a state equivalent to death.

Section 4.1.3: GBD 2013 European disability weights measurement study

The protocol for the European DWs measurement study was based on the methodology established in the GBD 2010 DWs measurement study. For the GBD 2010 study, a primary data collection effort was undertaken to measure health loss, rather than welfare loss, using a standardized methodology based on simple comparison questions directed to the general public across diverse communities. Between October 28, 2009, and June 23, 2010, multi-country household surveys were conducted in five countries—Bangladesh, Indonesia, Peru, Tanzania, and the United States—selected to capture a broad spectrum of cultural, linguistic, and socioeconomic diversity. Further methodological details are provided elsewhere. The estimation of DWs for GBD 2010 was heavily influenced by how health outcomes were described to survey participants. To balance validity and parsimony, the descriptions of health states were carefully designed, which necessitated omitting certain details about specific conditions. These lay descriptions were developed collaboratively by expert groups focused on

different health issues, leading to some variability in language and detail. Feedback, including criticisms and recommendations for improvement, was provided by several commentators, underscoring areas for refinement in the measurement of the GBD 2010 DWs.

The protocol for the European DWs measurement study adhered to the methodology established and applied in the GBD 2010 DWs measurement study. Lay descriptions for specific health states were revised to address inconsistencies in wording across severity levels and the omission of significant symptoms. The European DWs measurement study assessed 255 health states, 183 of which were analyzed in GBD 2013. Of these, 135 remained unchanged from the 220 health states included in the GBD 2010 DWs study, while 30 were drawn from GBD 2010 with revised lay descriptions. Additionally, DWs were developed for new sequelae introduced in GBD 2013 that had not been addressed in GBD 2010.

Given the strong correlation between DWs obtained from country-specific surveys and web-based surveys, the analysis incorporated results from both collectively. Pairwise comparison responses were analyzed using probit regression. Dummy variables were assigned to represent each pair: a value of 1 for the first health state, -1 for the second, and 0 for states not included in the pair. This approach formalized the concept that equally severe health states would yield evenly split responses, whereas states with significant differences in severity would yield asymmetric responses. Statistical methods were applied to infer the relative distances between health states based on response frequencies. The next analytical step involved anchoring these relative estimates onto the 0–1 DW scale, where 0 represents no health loss, and 1 represents health loss equivalent to death. Anchoring was achieved using population health equivalence data from the GBD 2010 web survey. Linear regression was performed on the probit coefficients derived from paired comparisons against logit-transformed DW estimates obtained from interval regressions of population health equivalence

responses. Mean DW values on the 0–1 scale were estimated through numerical integration, and uncertainty was quantified using bootstrapping with 1,000 samples.

Section 4.1.4: Comorbidity correction (COMO)

The final step in estimating YLDs involves a microsimulation process known as "COMO" (comorbidity correction). For both GBD 2019 and 2021, this process addresses the co-occurrence of various diseases by simulating 20,000 individuals for each location-age-sex-year combination, considering the independent probability of having any of the sequelae included in GBD based on their prevalence.

The two essential components required for computing YLDs and serving as inputs for COMO are:

1) the prevalence of each disease sequela, and
2) DWs.

Prevalence values for causes are primarily derived using DisMod-MR 2.1 and, for causes with multiple sequelae, subsequently allocated into sequela-specific prevalence based on available estimates of the severity distribution. The estimation of DWs and severity distributions has been detailed earlier in this appendix.

The microsimulation process, conducted for each age-sex-location-year combination, can be succinctly described as a four-step procedure. Firstly, simulated individuals (referred to as "simulants") are exposed to independent probabilities corresponding to each sequela, where the probability equals the estimated prevalence. For each simulant, the likelihood of having a disease sequela aligns with the estimated prevalence, and their status of having or not having a

disease sequela is determined by a draw from a binomial distribution. As a result of this simulation, simulants may end up with any number of sequelae, ranging from 0 to the theoretical maximum given their demographic characteristics. Secondly, the DWs for each simulant are calculated based on the disease sequelae they have acquired. The cumulative DW for a simulant is derived using a formula where it equals one minus the multiplicative sum of one minus each DW present.

Section 4.1.5: YLD computation

In GBD 2021, YLDs were calculated for each sequela by multiplying prevalence with the DWs associated with that particular health state. The uncertainty ranges reported around YLDs encompass uncertainties in both prevalence and DW. To achieve this, we utilized 500 samples of comorbidity-corrected YLDs and 500 samples of DW to generate a distribution of 500 YLD samples. We assumed no correlation in the uncertainty between prevalence and DWs. The 95% uncertainty interval (UI) is determined by the 25th and 975th values of this distribution.

The uncertainty intervals for YLDs at various time points (1990, 1995, 2000, 2005, 2010, 2015, 2020, and 2021) for a given disease or sequela are correlated due to the shared uncertainty in DW, and DW draws are not specific to years. Consequently, changes in YLDs over time may be significant even if the uncertainty intervals of the two YLD estimates mostly overlap, as prevalence uncertainty intervals are utilized to assess the significance of changes in YLDs over time since DW draws are not specific to years.

Section 5: Forecasting for 2050 estimates

Section 5.1: Overview of the forecasting framework

Over recent decades, multiple forecasting studies have been conducted using historical estimates from the GBD. Early GBD forecasts projected mortality and DALYs at the regional level for 1990–2020[1] and were later updated to extend projections to 2030.[2] In 2011, forecasts for communicable and non-communicable diseases through 2060 were developed, integrating GBD estimates with methodologies from the International Futures economic modelling framework. More recent studies have provided country-level forecasts of mortality and health drivers to 2040,[3] as well as detailed projections of population demographics and all-cause mortality for 195 countries and territories to 2100.[4] Building on this foundation, recent study extends GBD forecasts to include non-fatal disease burden, presenting estimates of years lived YLDs and DALYs for 204 countries and territories to 2050.[5] Additionally, projections of fatal disease burden—mortality, years of life lost, and life expectancy—are included.

In this study, we developed a forecasting model tailored to estimate the non-fatal disease burden of AMD. Using data from the GBD 2021, we projected prevalence by age, sex, and location from 2022 to 2050. Specifically, we estimated prevalence at the global level, disaggregated by sex, to provide a comprehensive understanding of disease burden. These projections serve to forecast the future burden of AMD from 2022 to 2050.

Section 5.2: Forecasting non-fatal disease burden

The GBD 2021 study independently modeled non-fatal disease burden for individual causes, tailored to the characteristics of each condition. Prevalence was primarily modeled using mixed-effects models, either by directly estimating prevalence or by leveraging mortality-to-incidence ratios to infer incidence rates. Specifically, non-fatal disease burden was modeled

using either mortality-to-incidence ratios or mortality-to-prevalence ratios, depending on the nature of the cause. For non-fatal conditions where mortality was not observed, prevalence was directly estimated using linear mixed-effects models to predict prevalence.

Section 5.2.1: Prevalence-only models

For causes where prevalence was directly modelled due to the absence of mortality data, such as in the case of age-related macular degeneration, the modelling approach is as follows:

$$\text{logit}(P_{a,s,l,y}) = \beta_0 + \beta_1 SDI_{l,y} + \pi_{0:a,s,l} + \epsilon_{a,s,l,y}$$

where $P_{a,s,l,y}$ is the age-sex-location-year specific prevalence of a given cause, with location-year specific SDI as the covariate for the fixed slope and $\pi_{0:a,s,l}$ as the age-sex-location specific random intercept.

Section 5.3: Ensemble modelling

Section 5.3.1: Ensemble modelling framework

To forecast, we applied a generalized ensemble modeling approach that incorporated 12 distinct sub-models. These sub-models utilized two primary modeling strategies: the annualized rate of change (ARC) and a two-stage spline model based on the Meta-Regression Bayesian Regularized Trimmed Tool (MR-BRT). Each sub-model employed six different recency-weighting parameters (omega) ranging from 0 to 2.5, where higher values assigned greater emphasis to more recent years.

For the ARC sub-models, the annual change was calculated by applying a logit transformation to the GBD 2021 AMD prevalence data, stratified by age, sex, and location, for

the years 1990 through 2021. To mitigate the impact of data noise, we replaced annual changes outside the 2.5th and 97.5th percentiles with the corresponding percentile values. The two-stage MR-BRT sub-models used the first stage to fit age-standardized, location-, and sex-specific logit of the prevalence on SDI:

$$logit(R_{a,s,l,t}) = \beta_0 + \beta_1 spline(SDI_{l,t}) + \epsilon_{a,s,l,t}$$

where $logit(R_{a,s,l,t})$ is the logit of the prevalence rate in age a, sex s, location l, and year t, $\beta 0$ is an intercept, $\beta 1$ is a coefficient matrix, *spline* is the spline with five knots placed evenly across the distribution of SDI data and it assumes both right and left linear tails, and $\epsilon_{a,s,l,t}$ is the residual. This is then followed by the second stage, where the logit of the residuals from the first stage was linearly modelled on time (year):

$$logit(\epsilon_{a,s,l,t}) = \beta_0 + year_t + \lambda + \Psi_{a,s,l,t}$$

where λ is a fixed intercept value, and $\Psi_{c,s,t}$ is an error term.

The weight assigned to each sub-model was determined through out-of-sample predictive validity experiments. Sub-models were trained using data from 1990 to 2009 and validated using data from 2010 to 2021. Performance of each sub-model was evaluated using root mean square error (RMSE), which was then used to calculate the sampling weights for each sub-model. Final sub-model forecasts were generated using a training dataset spanning 1990–2009 for each sub-model. For the ARC sub-models, the annualized change, adjusted with the respective recency-weighting parameter, was utilized to forecast prevalence rate for 2022–2050. In the MR-BRT sub-models, forecasted SDI values and recency weights were applied to project SEV values based on the model fit. The final ensemble forecasts were created by combining the outputs from the individual sub-models, weighted according to the sampling weights derived from the out-of-sample validation experiments (appendix figure B).

Appendix Figure C. Ensemble modelling framework.

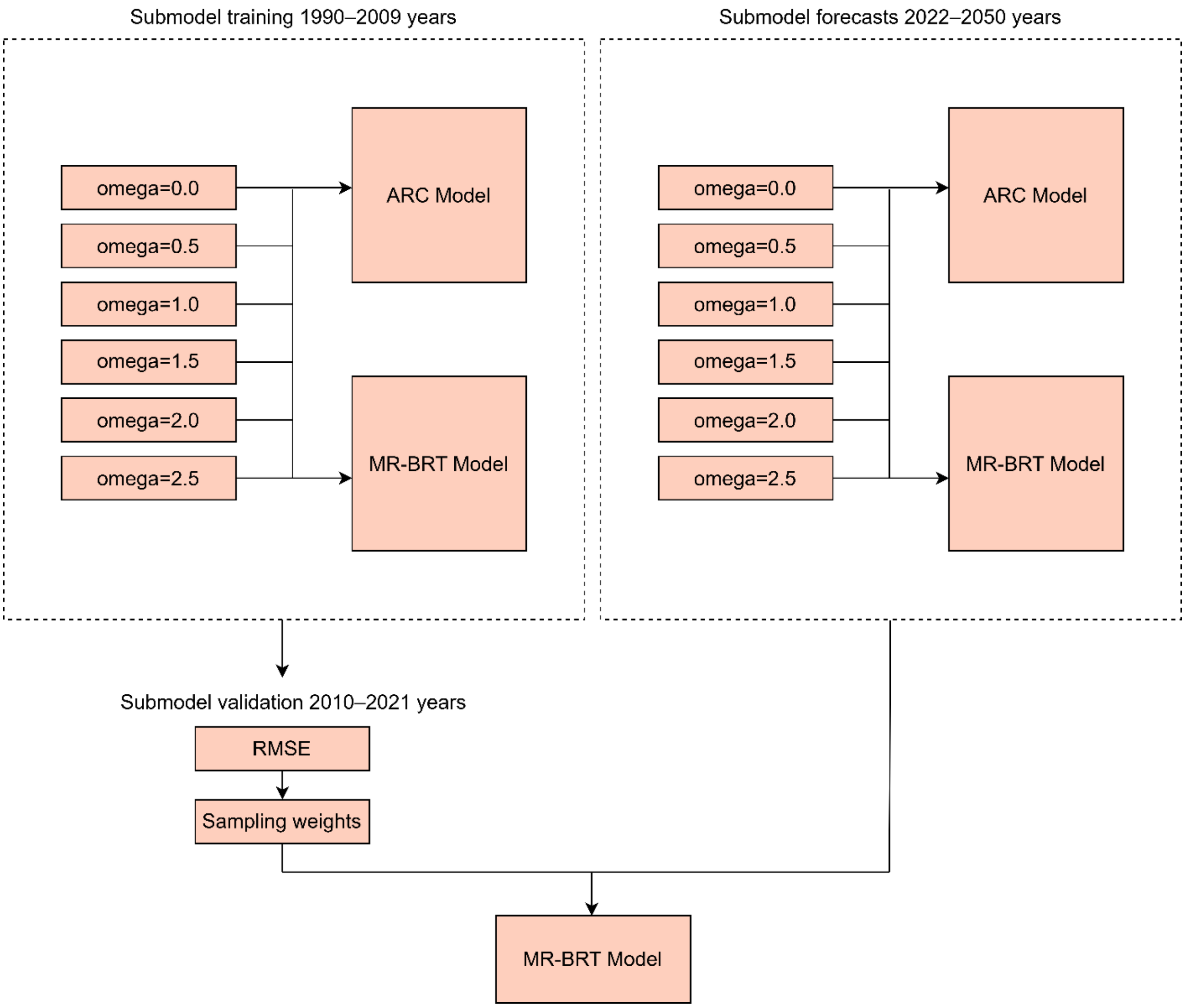

Section 5.3.2: ARC model

The ensemble model consisted of six ARC sub-models, each employing different recency-weighting parameters, where higher weights emphasized more recent years. The annual change in the logit-transformed prevalence rate was calculated, stratified by age, sex, and location. To mitigate the impact of noisy data, annual changes outside the 2.5^{th} and 97.5^{th} percentiles were replaced with the corresponding percentile values. Sub-model weights were determined through out-of-sample predictive validity experiments, where each sub-model was trained on data from 1990 to 2009 and validated using GBD estimates from 2010 to 2021. The performance of each sub-model was evaluated using RMSE, which informed the calculation

of sampling weights. Using the 1990–2021 training dataset, forecasts were generated for each sub-model, and the values were sampled based on the RMSE to produce the final ensemble forecasts.

Section 5.3.3: MR-BRT model

We forecasted prevalence rate for AMD with risk factor using an ensemble model comprising six annualised rate of change models and six MR-BRT spline models driven by SDI. Within each model branch, we varied recency-weighting schemes among the six sub-models, from treating all years equally (recency weight=0) to exponentially weighting the most recent trends more heavily. MR-BRT includes a range of statistical models—primarily non-linear and linear mixed effects models—and fitting procedures. The two-stage MR-BRT sub-models used the first stage to fit age-standardized, sex-specific logit of the prevalence on SDI using a third-order spline with five evenly spaced interior knots:

$$logit(R_{c,s,t}) = \beta_0 + \beta_1 spline(SDI_{c,t}) + \epsilon_{c,s,t}$$

where $logit(R_{c,s,t})$ is the logit of the age-standardized rate in country c, sex s and year t, $\beta 0$ is an intercept, $\beta 1$ is a coefficient matrix, *spline* is the spline with five knots placed evenly across the distribution of SDI data and it assumes both right and left linear tails, and $\epsilon_{c,s,t}$ is the residual. This is then followed by the second stage, where the logit of the residuals from the first stage was linearly modelled on time (year):

$$logit(\epsilon_{c,s,t}) = \beta_0 + year_t + \lambda + \Psi_{c,s,t}$$

where λ is a fixed intercept value, and $\Psi_{c,s,t}$ is an error term.

Each sub-model was trained using data from 1990 to 2009 and validated using data from 2010 to 2021, based on the GBD 2021 estimates. For the MR-BRT sub-models, forecasted SDI

values, combined with recency weights, were utilized to predict the SEV values based on the model fit. The performance of each sub-model was evaluated using RMSE, which informed the determination of sampling weights for each sub-model.

Section 5.4: Computing SEVs, PAFs, and scalars

Section 5.4.1: Risk exposure (summary exposure values)

A risk factor's summary exposure value (SEV) represents the prevalence of risk exposure weighted by relative risk (RR). To analyse the mediation effects among risk factors for a given outcome c, we began with the population attributable fraction (PAF) for a single risk *r*, defined as:

$$\boldsymbol{PAF_{cr}} = \frac{\boldsymbol{P[c] - P[c\,|\bar{r}]}}{\boldsymbol{P[c]}},$$

For computational simplicity, we used an alternative definition of PAF_i via SEV_i and maximum relative risk RR_i^{max}

$$PAF_i = \frac{1}{SEV_i(RR_i^{max} - 1) + 1}$$

Note that the original SEV_i is within [0, 1].

Section 5.4.2: Forecasting direct smoking exposure

The GBD 2021 provides information on risk-outcome pairs. For AMD, the GBD 2021 risk matrix was utilized, considering risks that meet its criteria.[6] Smoking was identified as the key risk factor for AMD based on this framework. Details of the risk selection criteria and the computation of the risk matrix are described in Section 6. To predict AMD prevalence through

2050 based on smoking exposure, we applied the PAF method. Using the formula for PAF, we integrated data on RR and SEV estimates for smoking, stratified by region, sex, five-year age groups, and year, for both current and former smokers. This comprehensive evaluation provided a robust basis for estimating PAF values. These PAF values were further incorporated into the MR-BRT framework to project AMD prevalence through 2050, allowing us to evaluate the long-term impact of smoking on AMD prevalence trends.

Section 5.4.3: Scalars

The scalar S_c is used to adjust or standardize the overall magnitude of a specific outcome. It facilitates the evaluation of risk factor contributions using PAF and is applied to refine the outcome adjustment process.

$$S_c = \frac{1}{1 - PAF_c}$$

This approach is consistent with the methodology outlined in Foreman et al.[7]

Section 5.5: Uncertainty interval estimation

Uncertainty arising from data inputs, estimated model parameters, and bias-correction procedures was accounted for at the age-sex-location-year level across all measures included in the multi-step estimation processes of the GBD framework (encompassing population, risk factors, causes of death, and non-fatal estimates). This methodology captures the uncertainty at each stage of modeling and propagates it throughout the entire estimation process. Point estimates were calculated as the mean of 500 draws from the final posterior draw distribution, while 95% uncertainty intervals (UIs) were derived from the 2.5th and 97.5th percentiles of the

distribution.

Section 5.6: Ensemble model construction

Section 5.6.1: Root mean squared error (RMSE)

RMSE is a statistical metric used to measure the average magnitude of error between observed and predicted values. It is calculated as the square root of the mean of the squared differences between these values. In this study, RMSE was utilized to evaluate the out-of-sample predictive performance of each sub-model, with lower RMSE values indicating higher accuracy. More specifically, RMSE was used to determine sampling weights for each sub-model within the ensemble framework. The final ensemble forecasts were constructed by integrating 500 draws generated by each sub-model, with weights assigned based on RMSE-derived performance metrics. Sub-models with lower RMSE values, reflecting superior performance, were given greater influence in the final ensemble predictions. This approach ensured that the most accurate sub-models contributed more significantly to the overall forecasts.

Advantage

- Performance optimization:

  By assigning weights to sub-models based on RMSE, the ensemble model prioritized the most accurate predictions, enhancing overall predictive reliability.

- Preservation of diversity:

  Incorporating multiple sub-models allowed the ensemble to integrate various modeling approaches and assumptions, reducing the risk of overfitting to any single methodology.

- Robust Forecasts:

The weighted integration of draws ensured that the final predictions captured the strengths of individual sub-models while mitigating their weaknesses.

Through this methodology, the ensemble model leveraged the strengths of individual sub-models while providing comprehensive and accurate forecasts for prevalence estimates during the 2022–2050 period. This approach balanced model diversity and precision to deliver robust predictions.

Section 5.7: Post-processing of the results of align with GBD 2021

To align the forecasts with the most recently generated GBD 2021 results, we implemented a calibration process to ensure that all burden prevalence estimates are consistent with the GBD 2021 data for the year 2021. This calibration involved two primary steps: reference adjustment and scenario adjustment.

- Reference adjustment

  The reference adjustment aligns the predicted values to the GBD 2021 data for the year 2021, using a combination of relative difference and absolute difference. The relative difference quantifies the proportional discrepancy between the predicted values and the GBD 2021 data, while the absolute difference represents the numerical discrepancy. The following formula was applied to combine these differences:

$$D_{ref} = \alpha * D_{relative} + (1 - \alpha) * D_{absolute}$$

  Here, α represents a weighting coefficient that balances the contributions of the relative and absolute differences. The combined difference D_{ref} ensures a calibrated alignment of the model predictions to the GBD 2021 reference data for 2021.

- Scenario adjustment

  The scenario adjustment extends the reference-adjusted values to future years (post-2021) to generate scenario-based predictions. This step involves incorporating PAF values to account for the impact of smoking on AMD prevalence. Two distinct scenarios were developed:

  - Smoking-included scenario: incorporates the influence of smoking on AMD prevalence using PAF-adjusted values.
  - Smoking-excluded scenario: assumes no contribution from smoking, providing a counterfactual estimate of AMD prevalence.

These adjustments ensure that the forecasted AMD prevalence aligns with the GBD 2021 data in the base year and accounts for different potential trajectories of smoking-related impacts on AMD prevalence.

Section 6: Risk factor

Section 6.1: Overview of risk factor

Since 1996, the GBD study has conducted analyses of attributable risk burden, initially including 10 risk factors. GBD 2021 presents age-, sex-, and location-specific estimates for 88 risk factors globally, regionally, and nationally from 1990 to 2021. While various research organizations and initiatives, such as the Non-Communicable Disease Risk Factor Collaboration in partnership with WHO and the Prospective Urban and Rural Epidemiology study, have provided valuable population-level evidence for specific risk factors or groups in certain populations, GBD offers a systematic analysis and evaluation of the health impacts of a comprehensive selection of risk factors across all countries and regions worldwide.

GBD 2021 has advanced previous GBD estimates of risk exposure levels, RR, and attributable risk burdens in several significant ways. For 211 risk-outcome pairs, it quantified the evidence for associations further by using burden of proof risk function (BPRF) analyses to address unexplained between-study heterogeneity in the input data. This approach yielded conservative interpretations of risk-outcome associations. To facilitate the interpretation and comparison of BPRF measures across risk factors, summary risk-outcome scores were calculated and mapped to a star-rating system (ranging from 1 to 5 stars), summarizing the strength of associations between risks and outcomes. These updates reflect new or updated systematic reviews conducted since 2019. Additionally, the methodology for theoretical minimum risk exposure levels (TMREL) was applied to systematically assess and SEV for these risk factors, further strengthening the GBD framework's ability to evaluate risk factors comprehensively and consistently.

Section 6.2: Smoking

Section 6.2.1: Smoking flowchart

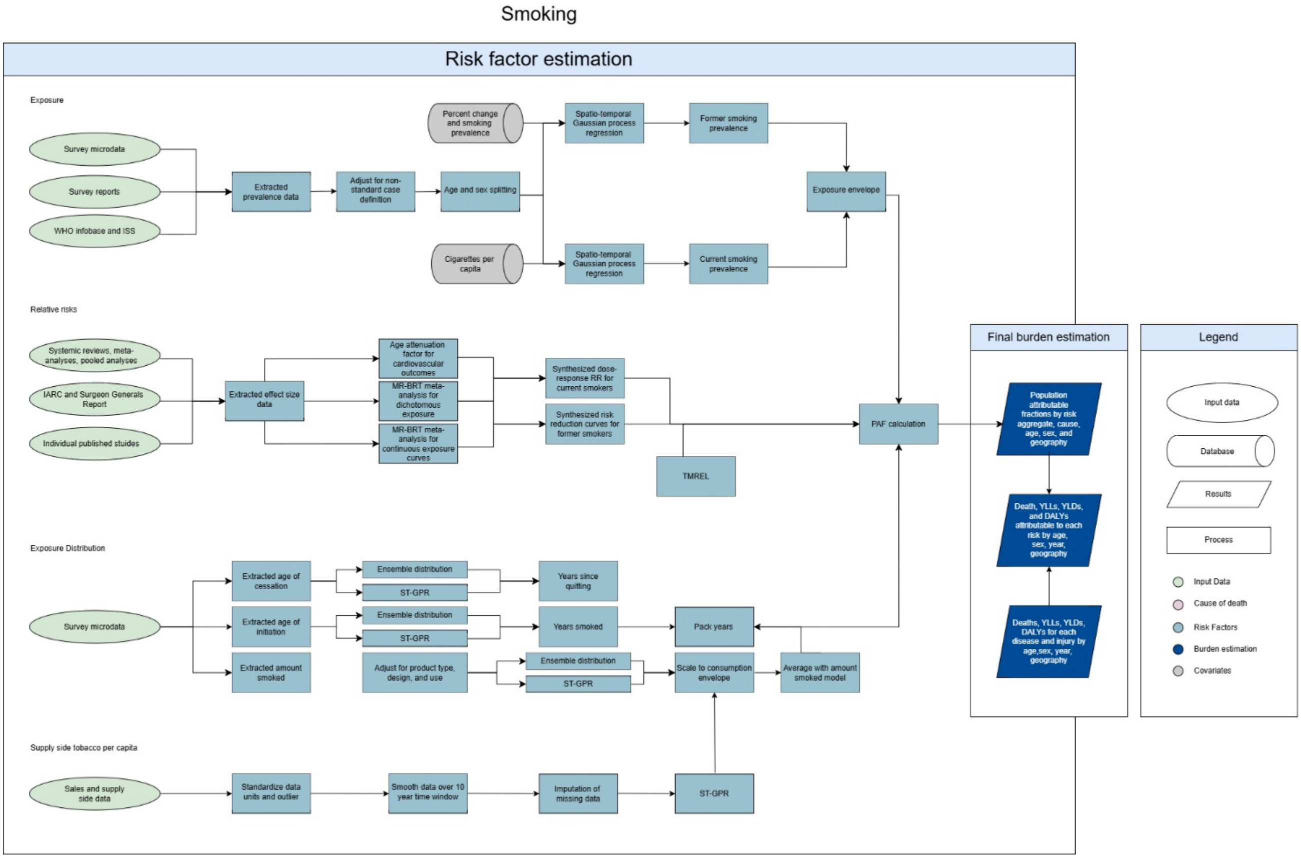

Section 6.2.2: Exposure and SEV

Definition and input data

Smoking prevalence in this study was defined as the proportion of individuals within a population currently or formerly using any smoked tobacco product. Current smoking was defined as the daily or occasional use of any smoked tobacco product, including cigarettes, hookah, cigars, pipes, or other forms of smoked tobacco. Former smoking was defined as the cessation of all smoked tobacco use for at least six months, whenever this information was available, or otherwise according to the definition provided by the respective data source. This definition was applied consistently across all surveys to ensure standardization and comparability of data.

To accommodate variations in survey methodologies, adjustments were made to align alternative definitions with the GBD standard definitions for smoking prevalence. Surveys providing broader definitions of smoking behaviors or mixed reporting formats were standardized using transformation coefficients derived from regression models. This approach ensured that all extracted data reflected a unified and comparable definition of smoking prevalence across different datasets and geographic regions.

Appendix Table C*. Data inputs for exposure for smoked tobacco*

	Countries with data	New sources	Total sources
Exposure	201	164	3603

Crosswalk

The GBD smoking case definitions were standardized as current smoking of any tobacco product and former smoking of any tobacco product. Any alternative case definitions were adjusted to align with these standardized definitions. For data sources that provided multiple case definitions, adjustment coefficients were developed to transform the alternative definitions into the GBD-standardized definitions. These adjustment coefficients were calculated as beta values from linear regression models with a single predictor and no intercept. As the same adjustment coefficients from GBD 2019 were used, a detailed explanation of the methodology has not been included in this appendix, as it has already been thoroughly documented in previous reports.[9]

Age and sex splitting

Prior to modeling, we ran a DisMod-MR 2.1 model with age-disaggregated data to derive age

patterns for countries. Subsequently, we employed these patterns to divide aggregated all-age data into the desired 5-year age groups for ST-GPR modeling. This process entailed computing a constant, denoted as "k," which represented the ratio of the all-age data point to the all-age estimated utilization rate.

$$k = \frac{\mu_{all\ age}}{\hat{\mu}}$$

The age-specific utilization rates were then multiplied by this constant, thereby integrating observation-specific and estimated age-pattern uncertainties into the final dataset. The segmented data were subsequently incorporated into the final DisMod-MR 2.1 model, $\hat{\mu}$.

Spatiotemporal Gaussian process regression (ST-GPR) modelling

The input data underwent modeling using Spatio-Temporal Gaussian Process Regression (ST-GPR) to smooth estimates across age, time, and location, particularly for incomplete datasets. Unlike linear models, ST-GPR assumes trends adhere to a Gaussian process characterized by a mean and covariance function. Initially, a DisMod-MR 2.1 model was executed to estimate age patterns, segmenting aggregated all-age data into 5-year age groups. This process entails computing a constant ratio and applying it to age-specific rates. The ST-GPR model integrated covariates and random effects while employing weights based on temporal, spatial, and age adjustments to mitigate variability and enhance accuracy. The final weights were normalized to ensure consistency across the dataset.

Section 6.2.3: Relative risk

Input data

GBD 2021 introduced key advancements in estimating risk factor exposure levels, RRs, and attributable burdens. Using burden of proof meta-regression methods, it accounted for differences in exposure ranges across comparison groups and utilized ensemble spline models

to capture non-linear risk–outcome relationships. BPRF analyses addressed unexplained heterogeneity in study data, yielding conservative interpretations of risk–outcome associations. Summary risk–outcome scores were mapped to a star rating system, with 37.9% of risk–outcome pairs rated as having moderate to very strong evidence (three to five stars). Mediation methods were updated to account for indirect relationships between risks and outcomes, while nitrogen dioxide air pollution was added as a new risk factor, along with 117 refined risk–outcome pairs informed by new evidence and updated methodologies.

To improve smoking-specific relative risk modeling, GBD 2021 conducted systematic reviews and meta-analyses of prospective cohort and case-control studies published between January 1, 1970, and May 31, 2022. Eligible studies reported relative risks, hazard ratios, or odds ratios for smoked tobacco exposure (e.g., pack-years or cigarettes per day) and GBD outcomes in the general population, with appropriate uncertainty measures. The refined search strategy enabled the inclusion of a broader range of studies, while data extraction focused on study design, confounder adjustments, and exposure measurement. These enhancements improved the precision of smoking-related RR curves, ensuring that the modeled risk–outcome relationships and attributable burden estimates were grounded in the most comprehensive and robust evidence available.

Appendix Table D*. Data inputs for exposure for smoked tobacco*

	Countries with data	New sources	Total sources
Relative risks	55	218	730

Exposure among current and former smokers

Exposure for current smokers was estimated using two continuous indicators: cigarettes smoked per smoker per day and pack-years. Pack-years combine smoking duration and

intensity, where one pack-year equals smoking one pack (20 cigarettes) daily for one year. Individual smoking histories were simulated based on distributions of smoking initiation age and quantity smoked, using cross-sectional survey data modeled with ST-GPR. Estimates of cigarettes per day were rescaled to match supply-side data, and pack-years were calculated by summing age- and time-specific distributions within birth cohorts to capture trends. For former smokers, exposure was estimated using years since cessation, with mean cessation age modeled using ST-GPR. Ensemble distributions of years since cessation were generated for all demographic groups, capturing the decline in exposure over time.

Theoretical minimum-risk exposure level

The theoretical minimum-risk exposure level, TMREL, for smoking is defined as zero exposure, meaning no use of any smoked tobacco products. This serves as the baseline for evaluating the health risks associated with smoking, enabling the calculation of PAFs and the overall burden of smoking-related diseases. The table below presents RR estimates specific to AMD, calculated based on the methods described above. These estimates were derived using the outlined modeling approaches, including exposure simulations, dose-response relationships, and adjustments for demographic and geographic variations.

Appendix Table E*. Relative risk estimates for age-related macular degeneration by cigarette consumption, sex, and age group from GBD 2021.*

**Risk**	**Category / Units**	**Sex**	**45-49 years**	**50-54 years**	**55-59 years**	**60-64 years**	**65-69 years**	**70-74 years**	**75-79 years**	**80-84 years**	**85-89 years**	**90-94 years**	**95+ years**
Age-related macular degeneration	0 Cigarettes Per Day	Female	1.00 (1.00 to 1.00)	1.00 (1.00 to 1.00)	1.00 (1.00 to 1.00)	1.00 (1.00 to 1.00)	1.00 (1.00 to 1.00)	1.00 (1.00 to 1.00)	1.00 (1.00 to 1.00)	1.00 (1.00 to 1.00)	1.00 (1.00 to 1.00)	1.00 (1.00 to 1.00)	1.00 (1.00 to 1.00)
Age-related macular degeneration	0 Cigarettes Per Day	Male	1.00 (1.00 to 1.00)	1.00 (1.00 to 1.00)	1.00 (1.00 to 1.00)	1.00 (1.00 to 1.00)	1.00 (1.00 to 1.00)	1.00 (1.00 to 1.00)	1.00 (1.00 to 1.00)	1.00 (1.00 to 1.00)	1.00 (1.00 to 1.00)	1.00 (1.00 to 1.00)	1.00 (1.00 to 1.00)
Age-related macular degeneration	10 Cigarettes Per Day	Female	1.45 (1.09	1.45 (1.09	1.45 (1.09	1.45 (1.09	1.45 (1.09	1.45 (1.09	1.45 (1.09	1.45 (1.09	1.45 (1.09	1.45 (1.09	1.45 (1.09

			to 1.88)	to 1.88)	to 1.88)	to 1.88)	to 1.88)	to 1.88)	to 1.88)	to 1.88)	to 1.88)	to 1.88)	to 1.88)
Age-related macular degeneration	10 Cigarettes Per Day	Male	1.45 (1.09 to 1.88)	1.45 (1.09 to 1.88)	1.45 (1.09 to 1.88)	1.45 (1.09 to 1.88)	1.45 (1.09 to 1.88)	1.45 (1.09 to 1.88)	1.45 (1.09 to 1.88)	1.45 (1.09 to 1.88)	1.45 (1.09 to 1.88)	1.45 (1.09 to 1.88)	1.45 (1.09 to 1.88)
Age-related macular degeneration	20 Cigarettes Per Day	Female	1.91 (1.39 to 2.48)	1.91 (1.39 to 2.48)	1.91 (1.39 to 2.48)	1.91 (1.39 to 2.48)	1.91 (1.39 to 2.48)	1.91 (1.39 to 2.48)	1.91 (1.39 to 2.48)	1.91 (1.39 to 2.48)	1.91 (1.39 to 2.48)	1.91 (1.39 to 2.48)	1.91 (1.39 to 2.48)
Age-related macular degeneration	20 Cigarettes Per Day	Male	1.91 (1.39 to 2.48)	1.91 (1.39 to 2.48)	1.91 (1.39 to 2.48)	1.91 (1.39 to 2.48)	1.91 (1.39 to 2.48)	1.91 (1.39 to 2.48)	1.91 (1.39 to 2.48)	1.91 (1.39 to 2.48)	1.91 (1.39 to 2.48)	1.91 (1.39 to 2.48)	1.91 (1.39 to 2.48)
Age-related macular degeneration	30 Cigarettes Per Day	Female	2.42 (1.71 to 3.14)	2.42 (1.71 to 3.14)	2.42 (1.71 to 3.14)	2.42 (1.71 to 3.14)	2.42 (1.71 to 3.14)	2.42 (1.71 to 3.14)	2.42 (1.71 to 3.14)	2.42 (1.71 to 3.14)	2.42 (1.71 to 3.14)	2.42 (1.71 to 3.14)	2.42 (1.71 to 3.14)
Age-related macular degeneration	30 Cigarettes Per Day	Male	2.42 (1.71 to 3.14)	2.42 (1.71 to 3.14)	2.42 (1.71 to 3.14)	2.42 (1.71 to 3.14)	2.42 (1.71 to 3.14)	2.42 (1.71 to 3.14)	2.42 (1.71 to 3.14)	2.42 (1.71 to 3.14)	2.42 (1.71 to 3.14)	2.42 (1.71 to 3.14)	2.42 (1.71 to 3.14)
Age-related macular degeneration	40 Cigarettes Per Day	Female	2.70 (1.70 to 3.89)	2.70 (1.70 to 3.89)	2.70 (1.70 to 3.89)	2.70 (1.70 to 3.89)	2.70 (1.70 to 3.89)	2.70 (1.70 to 3.89)	2.70 (1.70 to 3.89)	2.70 (1.70 to 3.89)	2.70 (1.70 to 3.89)	2.70 (1.70 to 3.89)	2.70 (1.70 to 3.89)
Age-related macular degeneration	40 Cigarettes Per Day	Male	2.70 (1.70 to 3.89)	2.70 (1.70 to 3.89)	2.70 (1.70 to 3.89)	2.70 (1.70 to 3.89)	2.70 (1.70 to 3.89)	2.70 (1.70 to 3.89)	2.70 (1.70 to 3.89)	2.70 (1.70 to 3.89)	2.70 (1.70 to 3.89)	2.70 (1.70 to 3.89)	2.70 (1.70 to 3.89)
Age-related macular degeneration	50 Cigarettes Per Day	Female	2.89 (1.58 to 4.65)	2.89 (1.58 to 4.65)	2.89 (1.58 to 4.65)	2.89 (1.58 to 4.65)	2.89 (1.58 to 4.65)	2.89 (1.58 to 4.65)	2.89 (1.58 to 4.65)	2.89 (1.58 to 4.65)	2.89 (1.58 to 4.65)	2.89 (1.58 to 4.65)	2.89 (1.58 to 4.65)
Age-related macular degeneration	50 Cigarettes Per Day	Male	2.89 (1.58 to 4.65)	2.89 (1.58 to 4.65)	2.89 (1.58 to 4.65)	2.89 (1.58 to 4.65)	2.89 (1.58 to 4.65)	2.89 (1.58 to 4.65)	2.89 (1.58 to 4.65)	2.89 (1.58 to 4.65)	2.89 (1.58 to 4.65)	2.89 (1.58 to 4.65)	2.89 (1.58 to 4.65)
Age-related macular degeneration	60 Cigarettes Per Day	Female	3.08 (1.46 to 5.42)	3.08 (1.46 to 5.42)	3.08 (1.46 to 5.42)	3.08 (1.46 to 5.42)	3.08 (1.46 to 5.42)	3.08 (1.46 to 5.42)	3.08 (1.46 to 5.42)	3.08 (1.46 to 5.42)	3.08 (1.46 to 5.42)	3.08 (1.46 to 5.42)	3.08 (1.46 to 5.42)
Age-related macular degeneration	60 Cigarettes Per Day	Male	3.08 (1.46 to 5.42)	3.08 (1.46 to 5.42)	3.08 (1.46 to 5.42)	3.08 (1.46 to 5.42)	3.08 (1.46 to 5.42)	3.08 (1.46 to 5.42)	3.08 (1.46 to 5.42)	3.08 (1.46 to 5.42)	3.08 (1.46 to 5.42)	3.08 (1.46 to 5.42)	3.08 (1.46 to 5.42)

Section 7: Healthcare Access and Quality Index

Section 7.1: Overview

The Healthcare Access and Quality (HAQ) Index is a comprehensive metric designed to evaluate personal health-care access and quality across diverse regions. Building on the GBD 2019, the HAQ Index was assessed both overall and for three select age groups: young (0–14 years), working age (15–64 years), and post-working age (65+ years), across 204 locations from 1990 to 2021. Recent refinements include adding new systematic reviews for risk–outcome pairs, enabling a more detailed and accurate representation of conditions where health-care access and quality are critical. These enhancements improve the index's ability to identify areas requiring targeted health system interventions. Detailed methodological explanations of these advancements are provided elsewhere.[6]

Section 7.2: Utilizing HAQ index

Section 7.2.1: linear interpolation & extrapolation

HAQ Index Construction

The HAQ Index is constructed using age-specific mortality-to-incidence ratios (MIR) and risk-standardized death rates (RSDR), adjusted by adding an offset of one death per million to address cases of zero values in specific age-cause combinations. All RSDR and MIR values were log-transformed and scaled using the 1st and 99th percentiles, with 0 representing the worst outcomes and 100 the best. These calculations were performed separately for all estimates, countries, and years across 32 causes and four HAQ age groups. The HAQ Index captures performance differences by presenting the values for the best and worst performers in each group, conceptualizing health system performance relative to these benchmarks for 1990–2019. Differences in scaling for each group ensure that varying outcomes across age groups do not distort representation of health-care access and quality.

2021 HAQ estimates via linear interpolation and extrapolation

To estimate the HAQ Index for 2021, linear interpolation and extrapolation methods were applied based on values from 1990 to 2019. Linear interpolation was used to estimate missing data points within this range, while extrapolation extended the trends observed in 1990–2019 to predict values for 2021. The estimation formula used for extrapolation was:

$$HAQ_{2021} = HAQ_{2019} + \frac{HAQ_{2019} - HAQ_{1990}}{2019 - 1990} \times (2021 - 2019)$$

Where:

- HAQ_{1990} is the value of the HAQ index in 1990.
- HAQ_{2019} is the value of the HAQ index in 2019.
- The slope term $\frac{HAQ_{2019} - HAQ_{1990}}{2019 - 1990}$ represents the annual rate of change over the period.

The same approach was applied to the upper and lower bounds (e.g., $upper_{2021}$, $lower_{2021}$) to ensure consistency in uncertainty ranges. This methodology ensures continuity and comparability of HAQ Index values while extending insights into health-care system performance through 2021. By relying on observed trends and leveraging interpolation and extrapolation, this approach provides a robust framework for projecting health-care access and quality into recent years.

Section 7.2.2: Age groupings

This study builds on prior research by extending the analysis of the HAQ Index to examine variations in health-care access and quality across different stages of life. Specifically, it addresses two primary questions: (1) how does health-care access and quality differ by age group, and (2) to what extent does convergence or divergence occur over time across age groups? To answer these questions, the HAQ Index was computed separately for three distinct age groups: young (0–14 years), working age (15–64 years), and post-working age (65–74 years). These groupings align with the Organisation for Economic Co-operation and Development definition of the working-age population (15–64 years) and the upper age limit of 75 years identified by Nolte and McKee for amenable mortality.[7]

In addition to the overall HAQ Index (ages 0–74 years), these age groupings were chosen to reflect the unique dynamics of health-care access and quality across the life course. The working-age group captures access tied to employment and economic activity, while the post-working age group highlights health-care access linked to social health insurance and aging-related needs. The young group focuses on access to and quality of child health care. The age cap at 75 years ensures consistency with Nolte and McKee's framework, which identifies deaths beyond this age as less amenable to health-care interventions.

Following this structure, we further adapted the age groupings to align with the specific age distribution of AMD. This allowed us to map and apply the HAQ Index effectively to assess health-care access and quality in relation to AMD across relevant age groups.

References

1. Murray CJ, Lopez AD. Alternative projections of mortality and disability by cause 1990-2020: Global Burden of Disease Study. *Lancet* 1997; **349**(9064): 1498-504.

2. Mathers CD, Loncar D. Projections of global mortality and burden of disease from 2002 to 2030. *PLoS Med* 2006; **3**(11): e442.

3. Foreman KJ, Marquez N, Dolgert A, et al. Forecasting life expectancy, years of life lost, and all-cause and cause-specific mortality for 250 causes of death: reference and alternative scenarios for 2016–40 for 195 countries and territories. *The Lancet* 2018; **392**(10159): 2052-90.

4. Vollset SE, Goren E, Yuan CW, et al. Fertility, mortality, migration, and population scenarios for 195 countries and territories from 2017 to 2100: a forecasting analysis for the Global Burden of Disease Study. *Lancet* 2020; **396**(10258): 1285-306.

5. Vollset SE, Ababneh HS, Abate YH, et al. Burden of disease scenarios for 204 countries and territories, 2022–2050: a forecasting analysis for the Global Burden of Disease Study 2021. *The Lancet* 2024; **403**(10440): 2204-56.

6. Assessing performance of the Healthcare Access and Quality Index, overall and by select age groups, for 204 countries and territories, 1990-2019: a systematic analysis from the Global Burden of Disease Study 2019. *Lancet Glob Health* 2022; **10**(12): e1715-e43.

7. Nolte E, McKee M. Measuring the health of nations: analysis of mortality amenable to health care. *Bmj* 2003; **327**(7424): 1129.

Appendix figures

Figure S1. Vision impairment due to age-macular degeneration by constituent sequelae prevalence for both sexes combined, 1990–2021. (A) Total number of moderate vision loss, severe vision loss, and blindness. (B) Rates (per 100,000 population) of age-standardized and all-age moderate vision loss, severe vision loss, and blindness. Shaded regions indicate 95% uncertainty intervals.

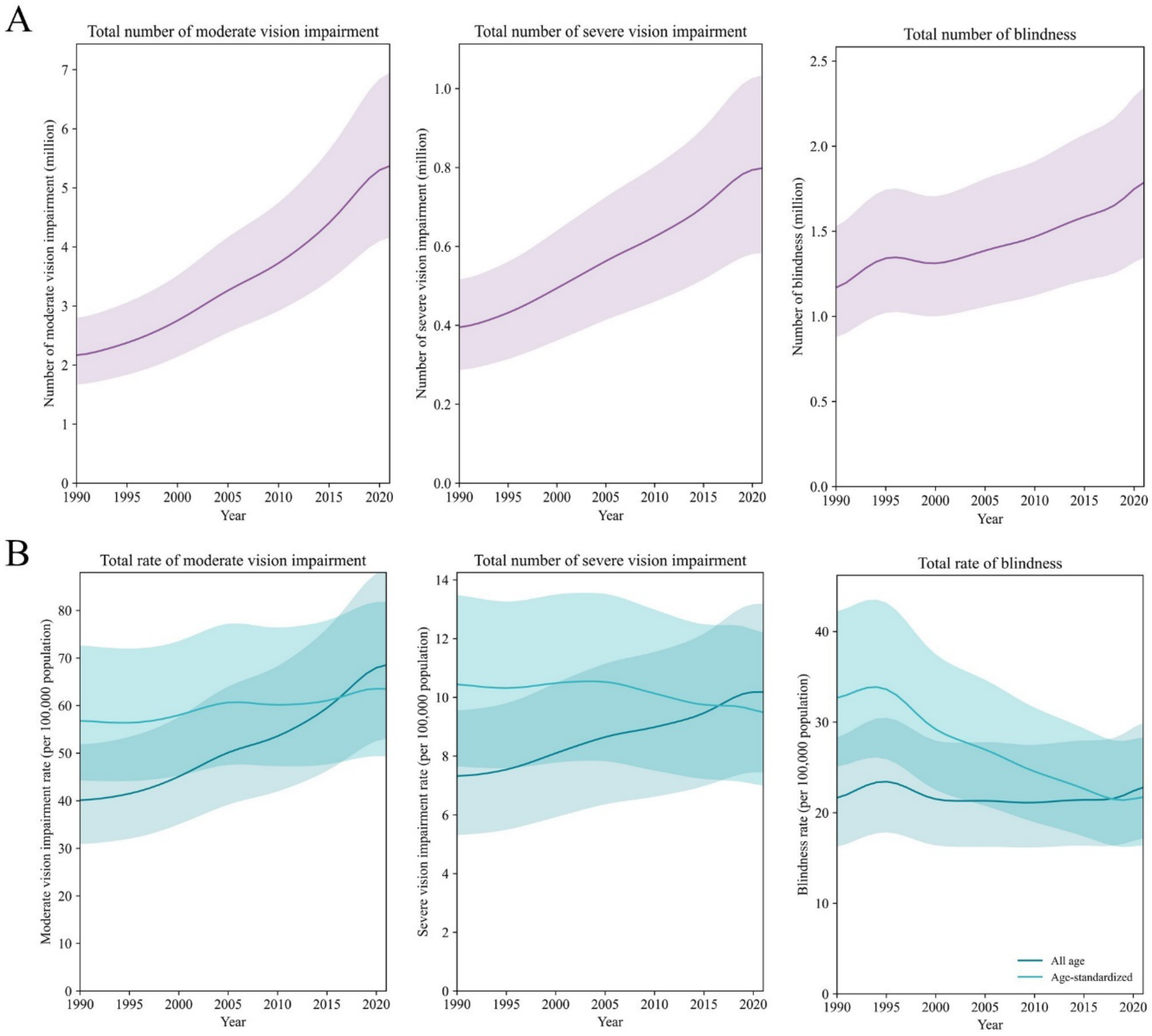

Figure S2. Vision impairment due to age-macular degeneration by constituent sequelae YLDs for both sexes combined, 1990–2021. (A) Total number of moderate vision loss, severe vision loss, and blindness. (B) Rates (per 100,000 population) of age-standardized and all-age moderate vision loss, severe vision loss, and blindness. Shaded regions indicate 95% uncertainty intervals. YLDs, years lived with disability.

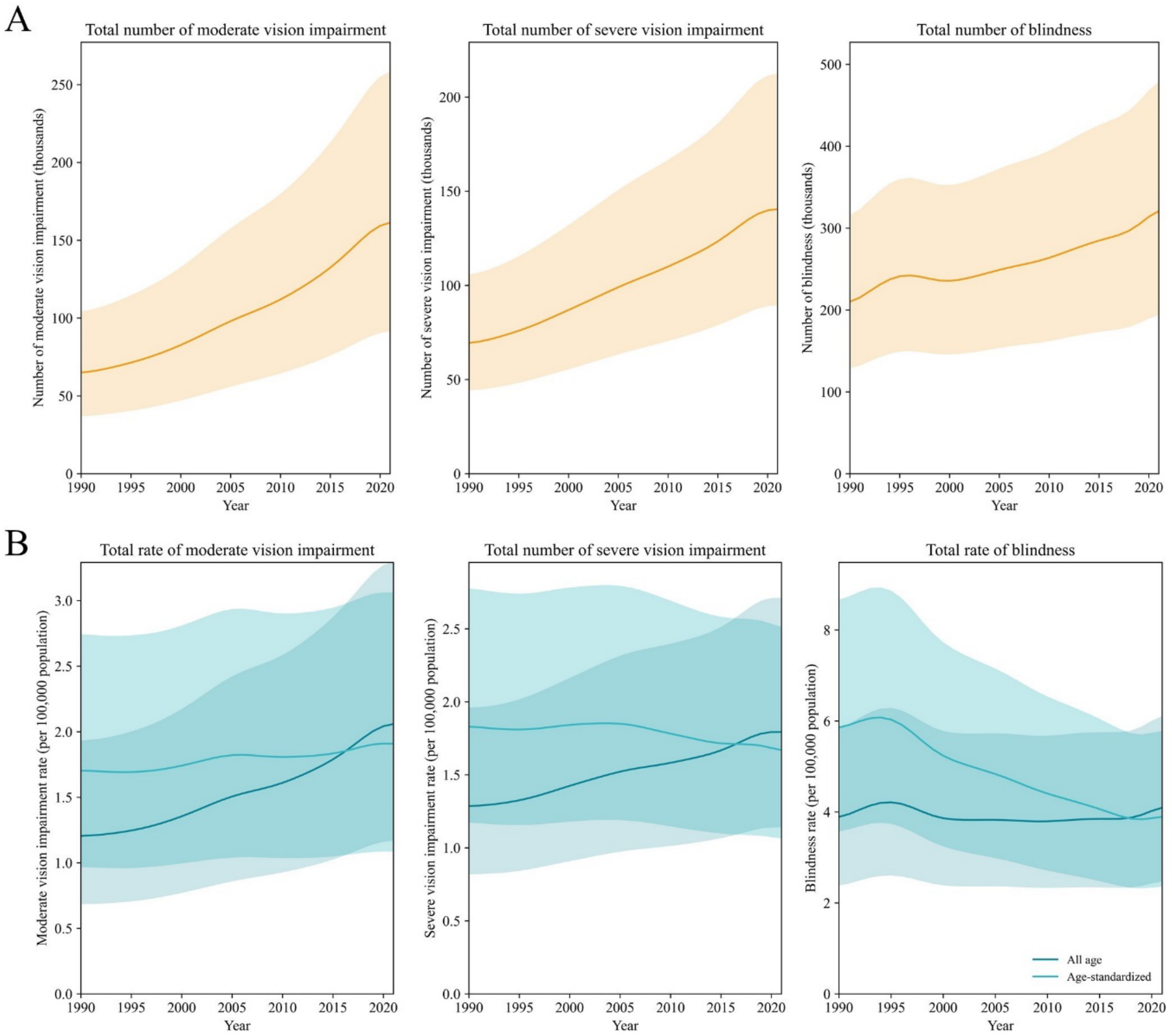

Figure S3. Association between Healthcare Access and Quality Index and age-standardized rate from age-related macular degeneration by the SDI groups, 2021. (A) Prevalence rate and (B) DALYs rate. The solid line represents the regression line, while the shaded region illustrates the 95% uncertainty intervals. DALYs, disability-adjusted life-years; SDI, Socio-demographic Index.

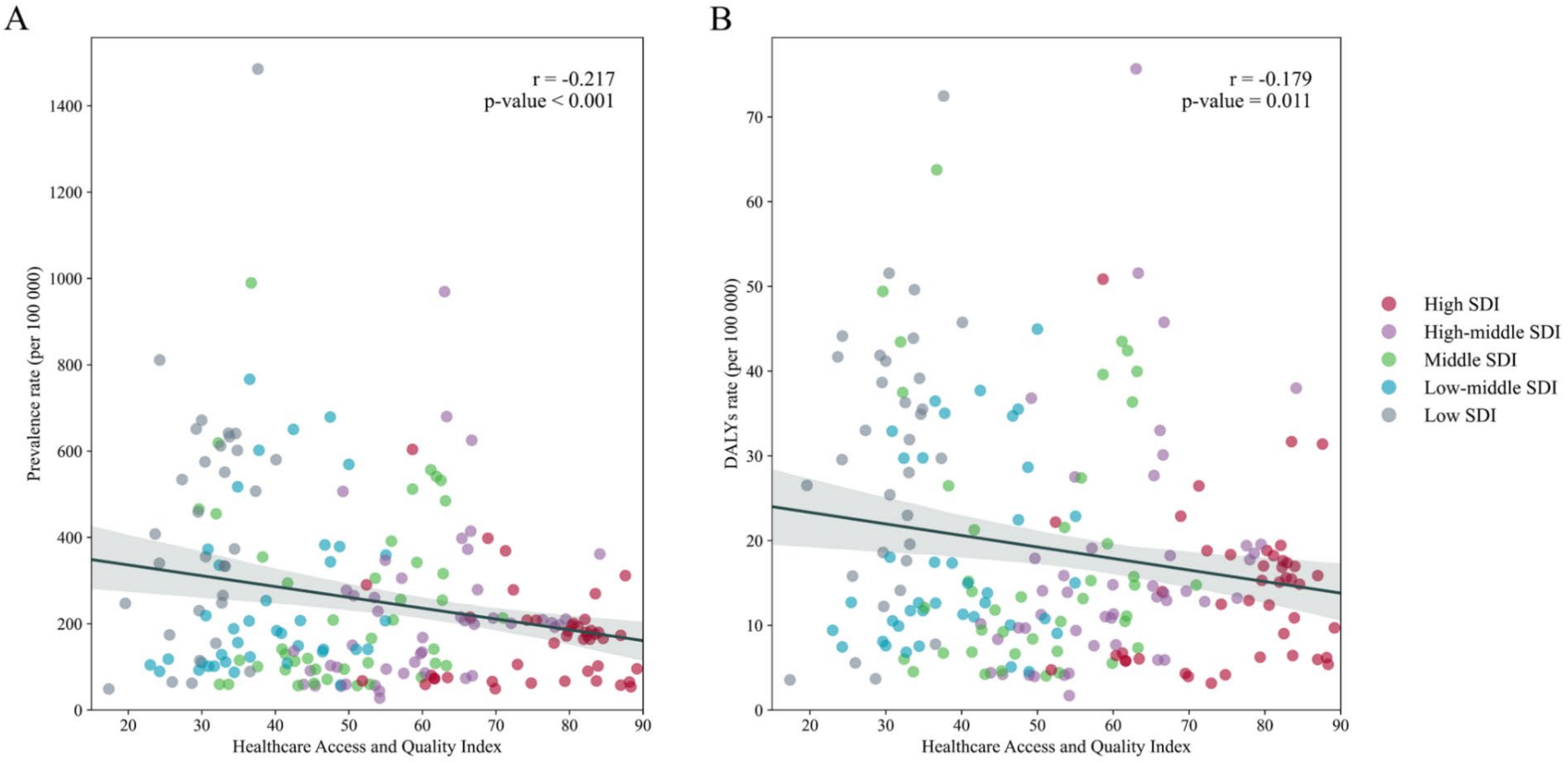

Appendix tables

Table S1. Checklist of information that should be included in new reports of global health estimates for GBD 2021

Item #	Checklist item	Reported on page #
**Objectives and funding**		
**1**	Define the indicator(s), populations (including age, sex, and geographic entities), and time period(s) for which estimates were made.	Methods, p.9
**2**	List the funding sources for the work.	Methods, p.15
**Data Inputs**		
For all data inputs from multiple sources that are synthesized as part of the study:		
**3**	Describe how the data were identified and how the data were accessed.	Methods, p.9-10
**4**	Specify the inclusion and exclusion criteria. Identify all ad-hoc exclusions.	Methods, p. 9-10
**5**	Provide information on all included data sources and their main characteristics. For each data source used, report reference information or contact name/institution, population represented, data collection method, year(s) of data collection, sex and age range, diagnostic criteria or measurement method, and sample size, as relevant.	Methods, p.9-15
**6**	Identify and describe any categories of input data that have potentially important biases (e.g., based on characteristics listed in item 5).	Methods, p.9-10
For data inputs that contribute to the analysis but were not synthesized as part of the study:		
**7**	Describe and give sources for any other data inputs.	Methods, p.9-10
For all data inputs:		
**8**	Provide all data inputs in a file format from which data can be efficiently extracted (e.g., a spreadsheet rather than a PDF), including all relevant meta-data listed in item 5. For any data inputs that cannot be shared because of ethical or legal reasons, such as third-party ownership, provide a contact name or the name of the institution that retains the right to the data.	Methods, p.9-10
**Data analysis**		
**9**	Provide a conceptual overview of the data analysis method. A diagram may be helpful.	Methods, p.9
**10**	Provide a detailed description of all steps of the analysis, including mathematical formulae. This description should cover, as relevant, data cleaning, data pre-processing, data adjustments and weighting of data sources, and mathematical or statistical model(s).	Methods, p.9-15
**11**	Describe how candidate models were evaluated and how the final model(s) were selected.	Methods, p.9-15

**12**	Provide the results of an evaluation of model performanc e, if done, as well as the results of any relevant sensitiv ity analysis.	Methods, p.10-15, Supplementary appendix 1
**13**	Describe methods for calculating uncertainty of the estim ates. State which sources of uncertainty were, and were not, accounted for in the uncertainty analysis.	Methods, p.12, Supplementary appendix 1
**14**	State how analytic or statistical source code used to gen erate estimates can be accessed.	Methods, p.10-15, Supplementary appendix 1
**Results and Discussion**		
**15**	Provide published estimates in a file format from which data can be efficiently extracted.	Results, p.16-22, Supplementary appendix 1
**16**	Report a quantitative measure of the uncertainty of the e stimates (e.g. uncertainty intervals).	Results, p.16-22, and Tables, p.37-45
**17**	Interpret results in light of existing evidence. If updating a previous set of estimates, describe the reasons for ch anges in estimates.	Results, p.16-22
**18**	Discuss limitations of the estimates. Include a discussion of any modelling assumptions or data limitations that aff ect interpretation of the estimates.	Discussion, p.27-28

Table S2. Global count (million) and percentage change of prevalence for age-related macular degeneration from 1990 to 2021

	Prevalence		
	**1990 (95% UI)**	**2021 (95% UI)**	**Annual percentage change between 1990 to 2021 (%)**
**Global**	**3.64 (3.04 to 4.35)**	**8.06 (6.71 to 9.82)**	**121.35 (114.01 to 128.68)**
**Sex**			
Male	1.50 (1.25 to 1.81)	3.40 (2.81 to 4.17)	126.77 (118.28 to 135.33)
Female	2.14 (1.79 to 2.54)	4.66 (3.88 to 5.65)	117.55 (110.65 to 124.95)
**SDI regions**			
High SDI	0.64 (0.54 to 0.76)	1.13 (0.95 to 1.35)	76.56 (70.87 to 82.22)
High-middle SDI	0.92 (0.77 to 1.09)	2.10 (1.76 to 2.56)	129.24 (119.21 to 139.85)
Middle SDI	1.04 (0.86 to 1.28)	2.79 (2.30 to 3.42)	167.90 (158.64 to 177.52)
Low-middle SDI	0.75 (0.61 to 0.91)	1.40 (1.14 to 1.73)	86.89 (79.27 to 94.56)
Low SDI	0.29 (0.24 to 0.34)	0.62 (0.51 to 0.77)	117.86 (108.71 to 128.03)
**Central Europe, Eastern Europe, and Central Asia**			
Central Asia	0.03 (0.03 to 0.04)	0.05 (0.04 to 0.07)	63.87 (57.00 to 71.35)
Central Europe	0.09 (0.08 to 0.11)	0.14 (0.12 to 0.18)	51.71 (45.52 to 57.99)
Eastern Europe	0.08 (0.07 to 0.10)	0.09 (0.07 to 0.11)	22.34 (18.15 to 26.63)
**High-income regions**			
Australasia	0.01 (0.01 to 0.01)	0.02 (0.02 to 0.03)	119.75 (105.63 to 134.06)
High-income Asian Pacific	0.04 (0.04 to 0.05)	0.12 (0.10 to 0.14)	167.42 (146.14 to 191.14)
High-income North America	0.11 (0.09 to 0.13)	0.20 (0.16 to 0.23)	77.78 (73.38 to 82.48)
Southern Latin America	0.02 (0.01 to 0.02)	0.03 (0.02 to 0.04)	77.52 (66.55 to 89.34)
Western Europe	0.65 (0.55 to 0.77)	0.94 (0.79 to 1.11)	44.05 (37.66 to 51.28)

**Latin America and Caribbean**			
Andean Latin America	0.02 (0.02 to 0.03)	0.07 (0.05 to 0.08)	193.13 (171.41 to 214.27)
Caribbean	0.01 (0.01 to 0.01)	0.01 (0.01 to 0.02)	96.12 (88.24 to 104.68)
Central Latin America	0.04 (0.03 to 0.05)	0.12 (0.10 to 0.15)	188.43 (176.55 to 199.43)
Tropical Latin America	0.07 (0.05 to 0.08)	0.20 (0.17 to 0.25)	202.94 (190.62 to 215.23)
**North Africa and Middle East**	0.28 (0.23 to 0.34)	0.73 (0.59 to 0.89)	158.50 (145.79 to 170.54)
**South Asia**	0.80 (0.65 to 0.97)	1.58 (1.29 to 1.96)	98.31 (88.83 to 108.12)
**Southeast Asia, East Asia, and Oceania**			
East Asia	0.89 (0.73 to 1.11)	2.68 (2.21 to 3.30)	199.59 (188.26 to 211.19)
Oceania	0.00 (0.00 to 0.00)	0.00 (0.00 to 0.00)	124.17 (112.82 to 136.44)
Southeast Asia	0.21 (0.17 to 0.26)	0.47 (0.38 to 0.56)	118.62 (104.60 to 133.16)
**Sub-Saharan Africa**			
Central Sub-Saharan Africa	0.01 (0.00 to 0.01)	0.01 (0.01 to 0.02)	153.20 (140.83 to 166.56)
Eastern Sub-Saharan Africa	0.08 (0.07 to 0.10)	0.17 (0.13 to 0.20)	101.80 (92.43 to 113.25)
Southern Sub-Saharan Africa	0.01 (0.01 to 0.01)	0.02 (0.02 to 0.03)	130.80 (124.29 to 137.94)
Western Sub-Saharan Africa	0.18 (0.15 to 0.22)	0.40 (0.32 to 0.50)	120.22 (111.50 to 128.49)

Abbreviations: SDI, Socio-demographic Index; UI, uncertainty interval.

Table S3. Global count (thousands) and annual percentage change of DALYs for age-related macular degeneration from 1990 to 2021

	DALYs		
	**1990 (95% UI)**	**2021 (95% UI)**	**Annual percentage change between 1990 to 2021 (%)**
**Global**	**302.90 (206.48 to 421.95)**	**578.02 (401.24 to 797.57)**	**90.83 (82.13 to 100.23)**
**Sex**			
Male	119.15 (80.94 to 167.89)	232.61 (159.81 to 323.23)	95.23 (85.29 to 107.04)
Female	183.75 (125.26 to 254.82)	345.41 (239.41 to 473.69)	87.98 (80.27 to 97.00)
**SDI regions**			
High SDI	61.28 (41.09 to 82.51)	95.84 (65.15 to 129.96)	56.41 (49.73 to 64.19)
High-middle SDI	77.00 (52.26 to 105.77)	147.38 (102.64 to 202.09)	91.40 (81.29 to 102.57)
Middle SDI	78.55 (53.55 to 110.86)	187.90 (129.23 to 259.07)	139.19 (127.99 to 153.39)
Low-middle SDI	62.34 (42.17 to 88.17)	101.90 (70.14 to 143.94)	63.46 (54.69 to 72.68)
Low SDI	23.49 (16.02 to 32.69)	44.62 (30.57 to 61.77)	89.97 (80.20 to 101.57)
**Central Europe, Eastern Europe, and Central Asia**			
Central Asia	2.24 (1.52 to 3.14)	3.49 (2.37 to 4.94)	56.05 (45.30 to 67.42)
Central Europe	6.88 (4.66 to 9.53)	9.51 (6.53 to 13.12)	38.24 (30.51 to 47.10)
Eastern Europe	6.34 (4.16 to 8.93)	7.00 (4.62 to 9.77)	10.57 (3.59 to 18.99)
**High-income regions**			
Australasia	1.10 (0.72 to 1.58)	2.20 (1.43 to 3.09)	99.59 (78.85 to 121.99)
High-income Asian Pacific	4.72 (3.00 to 6.64)	11.17 (7.47 to 15.41)	136.60 (113.90 to 165.63)
High-income North America	11.14 (7.51 to 15.33)	18.58 (12.73 to 25.35)	66.78 (59.97 to 74.78)
Southern Latin America	1.63 (1.03 to 2.29)	2.54 (1.66 to 3.51)	56.31 (40.40 to 76.58)
Western Europe	70.97 (47.10 to 96.61)	93.33 (62.84 to 127.28)	31.51 (24.21 to 40.11)
**Latin America and Caribbean**			

Andean Latin America	1.74 (1.18 to 2.45)	4.53 (3.13 to 6.31)	161.22 (134.97 to 187.19)
Caribbean	0.55 (0.36 to 0.78)	0.96 (0.64 to 1.36)	74.20 (57.62 to 91.18)
Central Latin America	3.65 (2.50 to 5.10)	9.25 (6.31 to 12.76)	153.26 (137.95 to 169.54)
Tropical Latin America	3.95 (2.75 to 5.44)	11.21 (7.87 to 15.36)	183.98 (168.48 to 199.90)
**North Africa and Middle East**	25.30 (17.22 to 35.83)	57.18 (38.43 to 79.96)	125.99 (114.44 to 138.43)
**South Asia**	66.82 (44.48 to 96.06)	113.53 (77.77 to 159.83)	69.90 (58.64 to 82.54)
**Southeast Asia, East Asia, and Oceania**			
East Asia	54.43 (37.57 to 76.21)	154.30 (106.46 to 213.90)	183.50 (170.01 to 199.01)
Oceania	0.09 (0.06 to 0.14)	0.18 (0.12 to 0.27)	99.72 (76.90 to 125.05)
Southeast Asia	18.69 (12.26 to 27.12)	35.07 (23.45 to 50.05)	87.59 (75.47 to 101.87)
**Sub-Saharan Africa**			
Central Sub-Saharan Africa	0.33 (0.22 to 0.48)	0.81 (0.54 to 1.16)	143.84 (117.02 to 175.47)
Eastern Sub-Saharan Africa	9.24 (6.14 to 12.99)	15.98 (10.62 to 22.80)	72.86 (63.89 to 82.41)
Southern Sub-Saharan Africa	0.76 (0.50 to 1.09)	1.66 (1.11 to 2.35)	116.57 (104.97 to 128.75)
Western Sub-Saharan Africa	12.33 (8.81 to 17.03)	25.53 (18.07 to 35.78)	107.11 (99.23 to 114.62)

Abbreviations: DALYs, disability-adjusted life-years; SDI, Socio-demographic Index; UI, uncertainty interval.

Table S4. Global count and rates (per 100,000 population) of prevalence for age-related macular degeneration at the global level by sex, from 1990 to 2021

	Prevalence					
	Overall (95% UI)		Male (95% UI)		Female (95% UI)	
**Year**	**Count, millions**	**Age-standardized rate (per 100,000)**	**Count, millions**	**Age-standardized rate (per 100,000)**	**Count, millions**	**Age-standardized rate (per 100,000)**
**1990**	3.64 (3.04 to 4.35)	99.50 (83.16 to 118.04)	1.50 (1.25 to 1.81)	92.23 (76.87 to 110.83)	2.14 (1.79 to 2.54)	104.34 (87.50 to 123.10)
**1991**	3.76 (3.14 to 4.49)	100.08 (83.72 to 118.76)	1.55 (1.28 to 1.87)	92.54 (77.29 to 111.17)	2.21 (1.85 to 2.62)	105.16 (88.06 to 124.11)
**1992**	3.87 (3.23 to 4.62)	100.51 (84.18 to 119.31)	1.59 (1.32 to 1.92)	92.78 (77.48 to 111.35)	2.28 (1.90 to 2.70)	105.78 (88.73 to 124.95)
**1993**	3.97 (3.32 to 4.75)	100.79 (84.41 to 119.68)	1.63 (1.35 to 1.98)	92.93 (77.60 to 111.44)	2.34 (1.96 to 2.78)	106.20 (89.22 to 125.58)
**1994**	4.07 (3.40 to 4.86)	100.88 (84.54 to 119.81)	1.68 (1.38 to 2.02)	92.94 (77.59 to 111.39)	2.40 (2.01 to 2.84)	106.39 (89.43 to 125.93)
**1995**	4.16 (3.48 to 4.97)	100.71 (84.41 to 119.63)	1.71 (1.42 to 2.07)	92.74 (77.40 to 111.04)	2.45 (2.05 to 2.90)	106.27 (89.36 to 125.92)
**1996**	4.24 (3.54 to 5.06)	100.13 (83.91 to 118.96)	1.75 (1.44 to 2.11)	92.27 (77.13 to 110.50)	2.49 (2.09 to 2.95)	105.64 (88.83 to 125.12)
**1997**	4.30 (3.59 to 5.13)	99.19 (83.25 to 117.85)	1.78 (1.48 to 2.15)	91.62 (76.61 to 109.72)	2.52 (2.12 to 2.99)	104.53 (87.96 to 123.75)
**1998**	4.36 (3.65 to 5.19)	98.20 (82.50 to 116.70)	1.81 (1.50 to 2.18)	90.99 (76.10 to 108.91)	2.55 (2.14 to 3.02)	103.32 (87.09 to 122.24)
**1999**	4.43 (3.72 to 5.28)	97.47 (81.96 to 115.77)	1.85 (1.54 to 2.23)	90.59 (75.67 to 108.47)	2.58 (2.18 to 3.06)	102.37 (86.32 to 121.01)
**2000**	4.53 (3.80 to 5.39)	97.26 (81.79 to 115.35)	1.90 (1.58 to 2.28)	90.60 (75.58 to 108.48)	2.63 (2.22 to 3.12)	102.04 (86.03 to 120.51)
**2001**	4.66 (3.91 to 5.55)	97.54 (82.02 to 115.65)	1.96 (1.63 to 2.36)	90.98 (75.89 to 108.98)	2.70 (2.28 to 3.21)	102.27 (86.25 to 120.73)
**2002**	4.80 (4.03 to 5.72)	97.90 (82.34 to 116.11)	2.02 (1.68 to 2.43)	91.43 (76.34 to 109.56)	2.78 (2.35 to 3.30)	102.58 (86.55 to 121.12)
**2003**	4.94 (4.16 to 5.90)	98.27 (82.69 to 116.68)	2.08 (1.74 to 2.51)	91.87 (76.78 to 110.03)	2.86 (2.42 to 3.40)	102.92 (86.86 to 121.64)
**2004**	5.09	98.55 (82.97 to 117.14)	2.15 (1.79 to 2.58)	92.18 (77.10 to 110.36)	2.94	103.20 (87.12 to 122.07)

	(4.28 to 6.08)				(2.49 to 3.49)	
**2005**	5.22 (4.39 to 6.24)	98.57 (83.04 to 117.28)	2.21 (1.84 to 2.65)	92.21 (77.18 to 110.37)	3.02 (2.55 to 3.58)	103.24 (87.19 to 122.18)
**2006**	5.35 (4.50 to 6.39)	98.15 (82.65 to 116.91)	2.26 (1.88 to 2.72)	91.79 (76.78 to 109.98)	3.09 (2.61 to 3.67)	102.85 (86.91 to 121.83)
**2007**	5.45 (4.59 to 6.52)	97.17 (81.78 to 115.91)	2.30 (1.92 to 2.77)	90.84 (75.94 to 108.97)	3.15 (2.67 to 3.74)	101.89 (86.15 to 120.81)
**2008**	5.54 (4.66 to 6.64)	96.04 (80.75 to 114.72)	2.34 (1.95 to 2.82)	89.74 (74.96 to 107.78)	3.20 (2.71 to 3.81)	100.77 (85.25 to 119.60)
**2009**	5.66 (4.75 to 6.77)	95.08 (79.85 to 113.73)	2.39 (1.99 to 2.88)	88.81 (74.19 to 106.80)	3.26 (2.77 to 3.88)	99.84 (84.52 to 118.62)
**2010**	5.79 (4.86 to 6.94)	94.58 (79.37 to 113.20)	2.45 (2.03 to 2.96)	88.32 (73.78 to 106.34)	3.34 (2.83 to 3.98)	99.36 (84.08 to 118.18)
**2011**	5.96 (4.99 to 7.14)	94.39 (79.20 to 113.13)	2.52 (2.09 to 3.04)	88.12 (73.56 to 106.14)	3.44 (2.91 to 4.10)	99.22 (83.82 to 118.21)
**2012**	6.12 (5.13 to 7.35)	94.02 (78.81 to 112.74)	2.59 (2.15 to 3.12)	87.75 (73.13 to 105.64)	3.53 (2.98 to 4.21)	98.88 (83.36 to 118.01)
**2013**	6.29 (5.27 to 7.55)	93.64 (78.40 to 112.29)	2.66 (2.21 to 3.21)	87.37 (72.67 to 105.13)	3.63 (3.05 to 4.33)	98.53 (82.88 to 117.79)
**2014**	6.48 (5.42 to 7.77)	93.39 (78.11 to 112.02)	2.74 (2.27 to 3.31)	87.10 (72.40 to 104.77)	3.73 (3.13 to 4.46)	98.31 (82.50 to 117.70)
**2015**	6.66 (5.57 to 7.99)	93.21 (77.88 to 111.84)	2.82 (2.34 to 3.41)	86.91 (72.28 to 104.50)	3.84 (3.22 to 4.60)	98.18 (82.24 to 117.63)
**2016**	6.88 (5.75 to 8.28)	93.29 (78.02 to 112.30)	2.92 (2.41 to 3.53)	86.94 (72.18 to 104.88)	3.97 (3.32 to 4.76)	98.31 (82.38 to 118.18)
**2017**	7.12 (5.93 to 8.61)	93.48 (78.18 to 112.99)	3.02 (2.50 to 3.67)	87.08 (72.17 to 105.46)	4.10 (3.43 to 4.95)	98.56 (82.49 to 118.96)
**2018**	7.37 (6.13 to 8.96)	93.80 (78.24 to 113.78)	3.13 (2.58 to 3.81)	87.32 (72.27 to 105.97)	4.25 (3.55 to 5.14)	98.97 (82.68 to 119.86)
**2019**	7.65 (6.35 to 9.31)	94.25 (78.43 to 114.50)	3.24 (2.67 to 3.96)	87.63 (72.43 to 106.50)	4.41 (3.68 to 5.35)	99.54 (83.05 to 120.88)
**2020**	8.04 (6.68 to 9.77)	96.11 (79.98 to 116.76)	3.43 (2.82 to 4.18)	89.80 (74.08 to 109.21)	4.61 (3.85 to 5.59)	101.15 (84.44 to 122.69)
**2021**	8.06 (6.71 to 9.82)	94.00 (78.32 to 114.42)	3.40 (2.81 to 4.17)	87.10 (71.99 to 106.29)	4.66 (3.88 to 5.65)	99.53 (82.98 to 120.72)

Abbreviations: UI, uncertainty interval.

Table S5. Global count and rates (per 100,000 population) of DALYs for age-related macular degeneration at the global level by sex, from 1990 to 2021

	DALYs					
	Overall (95% UI)		Male (95% UI)		Female (95% UI)	
**Year**	**Count, millions**	**Age-standardized rate (per 100,000)**	**Count, millions**	**Age-standardized rate (per 100,000)**	**Count, millions**	**Age-standardized rate (per 100,000)**
**1990**	0.30 (0.21 to 0.42)	8.38 (5.70 to 11.53)	0.12 (0.08 to 0.17)	7.37 (5.01 to 10.24)	0.18 (0.13 to 0.25)	9.04 (6.16 to 12.45)
**1991**	0.32 (0.21 to 0.44)	8.52 (5.80 to 11.74)	0.12 (0.08 to 0.18)	7.48 (5.08 to 10.38)	0.19 (0.13 to 0.27)	9.22 (6.29 to 12.71)
**1992**	0.33 (0.22 to 0.46)	8.62 (5.86 to 11.92)	0.13 (0.09 to 0.18)	7.55 (5.13 to 10.52)	0.20 (0.14 to 0.28)	9.34 (6.34 to 12.91)
**1993**	0.34 (0.23 to 0.47)	8.69 (5.94 to 12.03)	0.13 (0.09 to 0.19)	7.60 (5.19 to 10.56)	0.21 (0.14 to 0.29)	9.43 (6.40 to 13.01)
**1994**	0.35 (0.24 to 0.49)	8.72 (5.96 to 12.06)	0.14 (0.09 to 0.19)	7.62 (5.21 to 10.55)	0.21 (0.14 to 0.29)	9.47 (6.44 to 13.05)
**1995**	0.36 (0.24 to 0.50)	8.70 (5.95 to 12.04)	0.14 (0.09 to 0.20)	7.60 (5.19 to 10.55)	0.22 (0.15 to 0.30)	9.45 (6.45 to 13.05)
**1996**	0.36 (0.25 to 0.50)	8.59 (5.89 to 11.91)	0.14 (0.10 to 0.20)	7.51 (5.12 to 10.43)	0.22 (0.15 to 0.30)	9.34 (6.37 to 12.89)
**1997**	0.36 (0.25 to 0.50)	8.41 (5.75 to 11.62)	0.14 (0.10 to 0.20)	7.37 (5.01 to 10.25)	0.22 (0.15 to 0.30)	9.13 (6.26 to 12.63)
**1998**	0.36 (0.25 to 0.50)	8.20 (5.60 to 11.29)	0.14 (0.10 to 0.20)	7.21 (4.92 to 10.02)	0.22 (0.15 to 0.30)	8.89 (6.09 to 12.32)
**1999**	0.36 (0.25 to 0.50)	8.02 (5.49 to 11.04)	0.14 (0.10 to 0.20)	7.08 (4.86 to 9.81)	0.22 (0.15 to 0.30)	8.68 (5.94 to 11.99)
**2000**	0.37 (0.25 to 0.50)	7.92 (5.42 to 10.91)	0.15 (0.10 to 0.20)	7.01 (4.81 to 9.71)	0.22 (0.15 to 0.30)	8.56 (5.86 to 11.81)
**2001**	0.37 (0.26 to 0.51)	7.89 (5.41 to 10.81)	0.15 (0.10 to 0.21)	6.99 (4.82 to 9.61)	0.22 (0.15 to 0.31)	8.52 (5.85 to 11.68)
**2002**	0.38 (0.26 to 0.53)	7.85 (5.38 to 10.80)	0.15 (0.11 to 0.21)	6.98 (4.80 to 9.65)	0.23 (0.16 to 0.31)	8.47 (5.82 to 11.59)
**2003**	0.39 (0.27 to 0.54)	7.81 (5.36 to 10.74)	0.16 (0.11 to 0.22)	6.95 (4.77 to 9.59)	0.23 (0.16 to 0.32)	8.42 (5.79 to 11.57)
**2004**	0.40 (0.27 to 0.55)	7.77 (5.35 to 10.67)	0.16 (0.11 to 0.22)	6.93 (4.76 to 9.59)	0.24 (0.16 to 0.33)	8.37 (5.77 to 11.50)
**2005**	0.41 (0.28 to 0.56)	7.72 (5.33 to 10.61)	0.16 (0.11 to 0.23)	6.89 (4.77 to 9.52)	0.24 (0.17 to 0.33)	8.32 (5.73 to 11.42)
**2006**	0.41 (0.29 to 0.57)	7.66 (5.27 to 10.52)	0.17 (0.11 to 0.23)	6.82 (4.70 to 9.44)	0.25 (0.17 to 0.34)	8.25 (5.68 to 11.34)
**2007**	0.42 (0.29 to 0.58)	7.54 (5.21 to 10.37)	0.17 (0.12 to 0.24)	6.71 (4.65 to 9.28)	0.25 (0.17 to 0.34)	8.14 (5.61 to 11.18)
**2008**	0.42 (0.29 to 0.58)	7.41 (5.12 to 10.18)	0.17 (0.12 to 0.24)	6.59 (4.56 to 9.11)	0.25 (0.18 to 0.35)	8.00 (5.53 to 10.97)
**2009**	0.43 (0.30 to 0.59)	7.30 (5.05 to 10.06)	0.17 (0.12 to 0.24)	6.49 (4.49 to 8.99)	0.26 (0.18 to 0.35)	7.89 (5.45 to 10.83)
**2010**	0.44 (0.30 to 0.60)	7.22 (4.99 to 9.92)	0.18 (0.12 to 0.25)	6.42 (4.46 to 8.86)	0.26 (0.18 to 0.36)	7.82 (5.40 to 10.70)
**2011**	0.45 (0.31 to 0.62)	7.17 (4.96 to 9.87)	0.18 (0.13 to 0.25)	6.37 (4.42 to 8.84)	0.27 (0.19 to 0.37)	7.76 (5.35 to 10.66)
**2012**	0.46 (0.32 to 0.63)	7.10 (4.92 to 9.76)	0.19 (0.13 to 0.26)	6.31 (4.38 to 8.74)	0.27 (0.19 to 0.38)	7.69 (5.31 to 10.59)
**2013**	0.47 (0.33 to 0.65)	7.02 (4.88 to 9.66)	0.19 (0.13 to 0.26)	6.24 (4.33 to 8.63)	0.28 (0.19 to 0.38)	7.61 (5.26 to 10.43)
**2014**	0.48 (0.33 to 0.67)	6.96 (4.84 to 9.62)	0.19 (0.13 to 0.27)	6.19 (4.29 to 8.60)	0.29 (0.20 to 0.39)	7.54 (5.23 to 10.39)

**2015**	0.49 (0.34 to 0.68)	6.90 (4.80 to 9.50)	0.20 (0.14 to 0.28)	6.14 (4.27 to 8.49)	0.29 (0.20 to 0.40)	7.47 (5.19 to 10.26)
**2016**	0.50 (0.35 to 0.69)	6.84 (4.78 to 9.40)	0.20 (0.14 to 0.28)	6.09 (4.23 to 8.43)	0.30 (0.21 to 0.41)	7.40 (5.16 to 10.15)
**2017**	0.51 (0.36 to 0.71)	6.76 (4.71 to 9.29)	0.21 (0.14 to 0.29)	6.04 (4.18 to 8.33)	0.30 (0.21 to 0.42)	7.32 (5.08 to 10.04)
**2018**	0.52 (0.36 to 0.73)	6.70 (4.65 to 9.23)	0.21 (0.15 to 0.30)	5.99 (4.14 to 8.27)	0.31 (0.22 to 0.43)	7.25 (5.04 to 9.94)
**2019**	0.54 (0.37 to 0.74)	6.68 (4.63 to 9.18)	0.22 (0.15 to 0.31)	5.97 (4.12 to 8.27)	0.32 (0.22 to 0.44)	7.23 (5.02 to 9.92)
**2020**	0.56 (0.39 to 0.78)	6.77 (4.71 to 9.31)	0.23 (0.16 to 0.32)	6.04 (4.18 to 8.35)	0.33 (0.23 to 0.46)	7.34 (5.11 to 10.07)
**2021**	0.58 (0.40 to 0.80)	6.78 (4.70 to 9.32)	0.23 (0.16 to 0.32)	6.01 (4.15 to 8.29)	0.35 (0.24 to 0.47)	7.38 (5.11 to 10.12)

Abbreviations: DALYs, disability-adjusted life-years; UI, uncertainty interval.

Table S6. Global count and rates (per 100,000 population) of prevalence in three-categorized vision impairment due to age-macular degeneration from 1990 to 2021

	Prevalence of vision impairment					
	Moderate vision loss (95% UI)		Severe vision loss (95% UI)		Blindness (95% UI)	
**Year**	**Count, millions**	**Age-standardized rate (per 100,000)**	**Count, millions**	**Age-standardized rate (per 100,000)**	**Count, millions**	**Age-standardized rate (per 100,000)**
**1990**	2.13 (1.64 to 2.76)	56.98 (44.40 to 72.90)	0.39 (0.28 to 0.51)	10.49 (7.68 to 13.55)	1.12 (0.84 to 1.47)	32.02 (24.57 to 41.51)
**1991**	2.17 (1.67 to 2.81)	56.69 (44.21 to 72.48)	0.40 (0.29 to 0.52)	10.42 (7.63 to 13.45)	1.19 (0.89 to 1.55)	32.97 (25.31 to 42.51)
**1992**	2.22 (1.71 to 2.86)	56.49 (44.09 to 72.19)	0.40 (0.29 to 0.53)	10.36 (7.60 to 13.37)	1.25 (0.94 to 1.62)	33.65 (25.87 to 43.30)
**1993**	2.27 (1.75 to 2.92)	56.38 (44.04 to 72.02)	0.41 (0.30 to 0.54)	10.33 (7.58 to 13.30)	1.30 (0.98 to 1.69)	34.09 (26.21 to 43.83)
**1994**	2.32 (1.79 to 2.99)	56.32 (44.04 to 71.95)	0.42 (0.31 to 0.55)	10.31 (7.57 to 13.26)	1.33 (1.01 to 1.74)	34.25 (26.40 to 44.00)
**1995**	2.37 (1.83 to 3.05)	56.29 (44.04 to 71.90)	0.43 (0.31 to 0.56)	10.29 (7.57 to 13.22)	1.36 (1.04 to 1.77)	34.13 (26.32 to 43.83)
**1996**	2.43 (1.87 to 3.12)	56.35 (44.14 to 71.88)	0.44 (0.32 to 0.57)	10.30 (7.59 to 13.23)	1.37 (1.04 to 1.78)	33.48 (25.86 to 42.95)
**1997**	2.50 (1.93 to 3.21)	56.58 (44.30 to 72.03)	0.45 (0.33 to 0.59)	10.34 (7.62 to 13.29)	1.35 (1.03 to 1.75)	32.27 (24.87 to 41.41)
**1998**	2.57 (1.99 to 3.30)	56.94 (44.52 to 72.34)	0.47 (0.34 to 0.60)	10.40 (7.69 to 13.38)	1.32 (1.01 to 1.72)	30.86 (23.79 to 39.64)
**1999**	2.66 (2.06 to 3.39)	57.40 (44.81 to 72.78)	0.48 (0.35 to 0.62)	10.46 (7.75 to 13.47)	1.30 (0.99 to 1.68)	29.61 (22.83 to 37.99)
**2000**	2.74 (2.13 to 3.50)	57.89 (45.13 to 73.28)	0.49 (0.36 to 0.64)	10.50 (7.79 to 13.53)	1.30 (0.99 to 1.68)	28.88 (22.27 to 37.02)
**2001**	2.84 (2.21 to 3.62)	58.48 (45.61 to 74.14)	0.51 (0.37 to 0.66)	10.53 (7.82 to 13.56)	1.31 (1.00 to 1.71)	28.54 (22.01 to 36.59)
**2002**	2.95 (2.30 to 3.77)	59.21 (46.24 to 75.15)	0.52 (0.38 to 0.67)	10.54 (7.84 to 13.57)	1.33 (1.02 to 1.73)	28.16 (21.70 to 36.12)
**2003**	3.06 (2.38 to 3.91)	59.95 (46.88 to 76.21)	0.54 (0.39 to 0.69)	10.55 (7.85 to 13.57)	1.35 (1.03 to 1.76)	27.77 (21.38 to 35.64)

**2004**	3.17 (2.48 to 4.05)	60.60 (47.45 to 77.15)	0.55 (0.40 to 0.71)	10.56 (7.86 to 13.57)	1.37 (1.04 to 1.78)	27.39 (21.07 to 35.18)
**2005**	3.27 (2.56 to 4.18)	60.97 (47.80 to 77.74)	0.56 (0.41 to 0.73)	10.56 (7.87 to 13.57)	1.39 (1.06 to 1.81)	27.04 (20.79 to 34.75)
**2006**	3.36 (2.63 to 4.29)	61.00 (47.84 to 77.70)	0.58 (0.42 to 0.74)	10.53 (7.82 to 13.52)	1.41 (1.07 to 1.84)	26.62 (20.49 to 34.22)
**2007**	3.44 (2.70 to 4.39)	60.70 (47.66 to 77.22)	0.59 (0.43 to 0.76)	10.43 (7.74 to 13.38)	1.42 (1.08 to 1.85)	26.04 (20.08 to 33.49)
**2008**	3.52 (2.76 to 4.48)	60.30 (47.39 to 76.63)	0.60 (0.44 to 0.77)	10.31 (7.63 to 13.22)	1.43 (1.09 to 1.86)	25.43 (19.63 to 32.70)
**2009**	3.61 (2.83 to 4.59)	60.02 (47.20 to 76.20)	0.61 (0.45 to 0.78)	10.20 (7.55 to 13.07)	1.44 (1.10 to 1.88)	24.87 (19.22 to 31.98)
**2010**	3.71 (2.91 to 4.72)	59.98 (47.21 to 76.08)	0.62 (0.46 to 0.80)	10.13 (7.51 to 12.98)	1.46 (1.12 to 1.90)	24.47 (18.91 to 31.50)
**2011**	3.83 (3.00 to 4.89)	60.16 (47.24 to 76.41)	0.64 (0.47 to 0.82)	10.07 (7.46 to 12.89)	1.49 (1.14 to 1.94)	24.17 (18.66 to 31.13)
**2012**	3.96 (3.09 to 5.05)	60.25 (47.21 to 76.67)	0.65 (0.48 to 0.84)	9.96 (7.38 to 12.76)	1.52 (1.16 to 1.97)	23.81 (18.35 to 30.67)
**2013**	4.09 (3.19 to 5.22)	60.36 (47.18 to 76.86)	0.66 (0.49 to 0.85)	9.85 (7.30 to 12.61)	1.54 (1.18 to 2.01)	23.43 (18.04 to 30.22)
**2014**	4.23 (3.29 to 5.41)	60.56 (47.30 to 77.19)	0.68 (0.50 to 0.87)	9.76 (7.24 to 12.49)	1.56 (1.20 to 2.04)	23.07 (17.75 to 29.79)
**2015**	4.38 (3.41 to 5.60)	60.79 (47.46 to 77.54)	0.70 (0.52 to 0.90)	9.71 (7.21 to 12.43)	1.59 (1.22 to 2.08)	22.72 (17.47 to 29.36)
**2016**	4.56 (3.54 to 5.84)	61.32 (47.84 to 78.31)	0.72 (0.53 to 0.92)	9.70 (7.18 to 12.44)	1.61 (1.23 to 2.10)	22.27 (17.07 to 28.87)
**2017**	4.76 (3.69 to 6.11)	62.08 (48.38 to 79.41)	0.74 (0.55 to 0.96)	9.72 (7.17 to 12.47)	1.62 (1.23 to 2.11)	21.69 (16.56 to 28.19)
**2018**	4.98 (3.84 to 6.40)	62.90 (48.89 to 80.54)	0.77 (0.56 to 0.99)	9.73 (7.17 to 12.50)	1.63 (1.23 to 2.13)	21.17 (16.10 to 27.60)
**2019**	5.20 (4.01 to 6.68)	63.60 (49.33 to 81.52)	0.79 (0.58 to 1.02)	9.74 (7.16 to 12.51)	1.66 (1.26 to 2.18)	20.91 (15.86 to 27.35)
**2020**	5.48 (4.24 to 7.08)	65.14 (50.62 to 83.78)	0.82 (0.60 to 1.06)	9.75 (7.17 to 12.56)	1.74 (1.31 to 2.28)	21.23 (16.07 to 27.79)

**2021**	5.39 (4.18 to 7.00)	62.46 (48.59 to 80.76)	0.79 (0.58 to 1.03)	9.22 (6.81 to 11.84)	1.88 (1.41 to 2.46)	22.32 (16.81 to 29.05)

Abbreviations: UI, uncertainty interval.

Table S7. Global count and rates (per 100,000 population) of YLDs in three-categorized vision impairment due to age-macular degeneration from 1990 to 2021

	YLDs of vision impairment					
	Moderate vision loss (95% UI)		Severe vision loss (95% UI)		Blindness (95% UI)	
**Year**	**Count, thousands**	**Age-standardized rate (per 100,000)**	**Count, thousands**	**Age-standardized rate (per 100,000)**	**Count, thousands**	**Age-standardized rate (per 100,000)**
**1990**	64.02 (36.37 to 102.70)	1.71 (0.97 to 2.75)	68.41 (43.58 to 104.14)	1.84 (1.18 to 2.79)	201.04 (122.42 to 302.21)	5.74 (3.48 to 8.52)
**1991**	65.30 (37.07 to 105.08)	1.70 (0.97 to 2.74)	69.69 (44.42 to 106.45)	1.83 (1.17 to 2.77)	213.38 (131.08 to 321.34)	5.91 (3.61 to 8.74)
**1992**	66.67 (37.75 to 106.60)	1.70 (0.96 to 2.73)	71.04 (45.18 to 108.00)	1.82 (1.16 to 2.76)	224.09 (138.37 to 335.26)	6.04 (3.71 to 8.88)
**1993**	68.07 (38.61 to 109.03)	1.69 (0.96 to 2.72)	72.50 (45.94 to 110.02)	1.81 (1.16 to 2.75)	233.03 (143.87 to 348.22)	6.11 (3.78 to 9.00)
**1994**	69.59 (39.41 to 111.81)	1.69 (0.96 to 2.73)	74.07 (46.90 to 112.55)	1.81 (1.15 to 2.74)	240.06 (148.27 to 358.52)	6.14 (3.82 to 9.03)
**1995**	71.16 (40.31 to 114.46)	1.69 (0.96 to 2.74)	75.68 (48.08 to 115.25)	1.81 (1.15 to 2.74)	245.05 (151.57 to 365.50)	6.12 (3.79 to 8.99)
**1996**	72.91 (41.27 to 117.02)	1.69 (0.96 to 2.73)	77.48 (49.49 to 117.91)	1.81 (1.16 to 2.73)	245.96 (152.04 to 366.63)	6.01 (3.73 to 8.83)
**1997**	74.97 (42.43 to 120.50)	1.70 (0.96 to 2.75)	79.70 (50.68 to 121.37)	1.82 (1.15 to 2.75)	242.54 (150.18 to 361.61)	5.79 (3.59 to 8.50)
**1998**	77.28 (44.01 to 124.00)	1.71 (0.98 to 2.76)	82.09 (52.36 to 124.71)	1.83 (1.17 to 2.76)	237.22 (146.24 to 354.71)	5.54 (3.43 to 8.14)
**1999**	79.79 (45.33 to 127.72)	1.72 (0.98 to 2.78)	84.58 (54.23 to 128.47)	1.84 (1.18 to 2.78)	232.89 (144.30 to 349.09)	5.31 (3.30 to 7.85)
**2000**	82.38 (46.89 to 132.31)	1.74 (1.00 to 2.81)	86.95 (55.26 to 132.00)	1.84 (1.18 to 2.78)	232.69 (143.60 to 347.68)	5.18 (3.20 to 7.65)
**2001**	85.31 (48.55 to 136.83)	1.76 (1.00 to 2.83)	89.30 (56.93 to 135.36)	1.85 (1.19 to 2.79)	235.98 (146.00 to 353.44)	5.12 (3.17 to 7.55)
**2002**	88.58 (50.46 to 142.10)	1.78 (1.02 to 2.86)	91.71 (58.37 to 139.46)	1.85 (1.19 to 2.80)	239.21 (148.07 to 358.39)	5.05 (3.13 to 7.47)
**2003**	91.92 (52.29 to 147.81)	1.80 (1.03 to 2.91)	94.13 (60.57 to 142.95)	1.85 (1.20 to 2.80)	242.20 (148.72 to 361.64)	4.98 (3.06 to 7.33)
**2004**	95.29 (54.09 to 153.25)	1.82 (1.04 to 2.93)	96.64 (61.93 to 147.25)	1.86 (1.20 to 2.81)	245.59 (151.41 to 366.78)	4.91 (3.03 to 7.25)
**2005**	98.29 (56.00 to 158.15)	1.83 (1.04 to 2.95)	99.16 (63.18 to 151.15)	1.86 (1.18 to 2.80)	249.22 (154.12 to 373.31)	4.85 (3.00 to 7.17)
**2006**	101.03 (57.74 to 162.66)	1.83 (1.05 to 2.95)	101.59 (65.16 to 153.82)	1.85 (1.19 to 2.78)	252.69 (156.16 to 379.30)	4.77 (2.95 to 7.07)
**2007**	103.46 (59.26 to 166.27)	1.82 (1.04 to 2.93)	103.60 (66.14 to 158.12)	1.83 (1.18 to 2.78)	254.86 (157.36 to 380.55)	4.67 (2.88 to 6.92)
**2008**	105.73 (60.82 to 170.50)	1.81 (1.04 to 2.92)	105.40 (67.75 to 160.22)	1.81 (1.17 to 2.74)	256.57 (158.41 to 385.24)	4.56 (2.82 to 6.76)
**2009**	108.35 (62.49 to 174.00)	1.80 (1.04 to 2.90)	107.42 (68.92 to 163.22)	1.79 (1.16 to 2.72)	258.86 (159.19 to 386.52)	4.46 (2.75 to 6.60)
**2010**	111.42 (64.20 to 178.50)	1.80 (1.03 to 2.89)	109.78 (70.34 to 166.03)	1.78 (1.15 to 2.69)	262.77 (161.02 to 393.40)	4.39 (2.70 to 6.53)
**2011**	115.15 (66.35 to 184.93)	1.81 (1.04 to 2.90)	112.40 (71.58 to 170.59)	1.77 (1.13 to 2.68)	267.78 (163.92 to 400.03)	4.34 (2.66 to 6.43)
**2012**	118.98 (68.34 to 190.83)	1.81 (1.04 to 2.91)	114.82 (73.81 to 172.95)	1.75 (1.13 to 2.63)	272.45 (167.79 to 408.31)	4.27 (2.63 to 6.36)
**2013**	122.90 (70.53 to 197.20)	1.81 (1.04 to 2.91)	117.11 (74.85 to 176.62)	1.73 (1.11 to 2.61)	276.77 (169.15 to 413.69)	4.20 (2.57 to 6.25)

**2014**	127.21 (72.89 to 204.79)	1.82 (1.04 to 2.93)	119.70 (76.82 to 180.37)	1.72 (1.10 to 2.59)	281.26 (171.60 to 420.69)	4.14 (2.54 to 6.15)
**2015**	131.61 (75.51 to 211.66)	1.83 (1.04 to 2.93)	122.68 (78.68 to 185.03)	1.71 (1.10 to 2.58)	285.66 (173.63 to 426.54)	4.08 (2.49 to 6.08)
**2016**	136.98 (78.57 to 220.16)	1.84 (1.05 to 2.96)	126.49 (80.84 to 190.33)	1.71 (1.09 to 2.57)	288.92 (176.05 to 431.72)	4.00 (2.44 to 5.94)
**2017**	143.13 (81.82 to 229.64)	1.87 (1.07 to 3.00)	130.79 (82.89 to 197.05)	1.71 (1.08 to 2.57)	290.42 (175.18 to 435.53)	3.89 (2.35 to 5.80)
**2018**	149.67 (85.31 to 239.28)	1.89 (1.08 to 3.03)	135.12 (85.57 to 203.49)	1.71 (1.09 to 2.56)	292.48 (176.30 to 436.10)	3.80 (2.30 to 5.65)
**2019**	156.21 (88.62 to 250.08)	1.91 (1.09 to 3.07)	139.49 (88.77 to 210.83)	1.71 (1.09 to 2.58)	298.44 (179.87 to 446.01)	3.75 (2.26 to 5.58)
**2020**	164.71 (93.42 to 264.03)	1.96 (1.11 to 3.13)	143.71 (91.48 to 218.17)	1.71 (1.10 to 2.59)	311.88 (188.17 to 465.77)	3.81 (2.30 to 5.66)
**2021**	162.02 (92.09 to 259.40)	1.88 (1.07 to 3.01)	139.33 (88.61 to 210.23)	1.62 (1.03 to 2.44)	336.76 (203.86 to 501.91)	4.00 (2.43 to 5.93)

Abbreviations: YLDs, years lived with disability; UI, uncertainty interval.

Table S8. Global count and rates (per 100,000 population) of prevalence from age-related macular degeneration by age group and sex, 2021

	Prevalence					
	Overall (95% UI)		Male (95% UI)		Female (95% UI)	
	**Count, thousands**	**Rate (per 100,000)**	**Count, thousands**	**Rate (per 100,000)**	**Count, thousands**	**Rate (per 100,000)**
**All ages**	**8058 (6705 to 9823)**	**102.11 (84.97 to 124.48)**	**3399.69 (2808.35 to 4167.12)**	**85.86 (70.93 to 105.25)**	**4657.83 (3881.91 to 5650.81)**	**118.46 (98.73 to 143.71)**
**Age, years**						
**45–49**	19.07 (11.32 to 28.90)	4.03 (2.39 to 6.10)	8.75 (5.10 to 13.28)	3.68 (2.15 to 5.58)	10.31 (6.19 to 15.54)	4.38 (2.63 to 6.60)
**50–54**	189.10 (129.07 to 255.85)	42.50 (29.01 to 57.50)	85.98 (58.59 to 116.27)	38.73 (26.40 to 52.38)	103.12 (71.21 to 138.79)	46.25 (31.94 to 62.26)
**55–59**	635.57 (457.26 to 851.42)	160.61 (115.55 to 215.15)	287.53 (206.22 to 384.22)	147.66 (105.90 to 197.31)	348.04 (250.51 to 462.13)	173.15 (124.63 to 229.91)
**60–64**	1121.46 (807.56 to 1540.57)	350.40 (252.32 to 481.35)	505.15 (362.26 to 694.54)	324.78 (232.91 to 446.55)	616.31 (445.57 to 839.84)	374.63 (270.84 to 510.51)
**65–69**	1554.18 (1179.51 to 2027.66)	563.43 (427.60 to 735.08)	693.63 (521.56 to 903.66)	526.14 (395.62 to 685.46)	860.55 (657.50 to 1124.20)	597.57 (456.57 to 780.65)
**70–74**	1498.30 (1161.98 to 1953.67)	727.90 (564.51 to 949.13)	660.91 (510.26 to 865.10)	685.66 (529.37 to 897.49)	837.39 (651.34 to 1086.88)	765.10 (595.12 to 993.05)
**75–79**	1190.22 (944.33 to 1499.31)	902.47 (716.03 to 1136.84)	509.93 (402.32 to 644.66)	852.92 (672.93 to 1078.26)	680.29 (540.41 to 856.54)	943.57 (749.56 to 1188.03)
**80–84**	917.31 (710.15 to 1166.10)	1047.36 (810.83 to 1331.42)	358.36 (274.26 to 457.44)	977.73 (748.29 to 1248.07)	558.95 (435.11 to 704.57)	1097.46 (854.31 to 1383.38)
**85–89**	576.23 (456.94 to 713.12)	1260.30 (999.40 to 1559.69)	196.13 (152.05 to 243.28)	1136.80 (881.29 to 1410.07)	380.10 (305.89 to 469.07)	1335.13 (1074.46 to 1647.63)
**90–94**	259.49 (205.89 to 331.39)	1450.52 (1150.89 to 1852.43)	72.04 (56.51 to 91.42)	1235.97 (969.63 to 1568.55)	187.45 (149.69 to 238.45)	1554.20 (1241.10 to 1977.08)
**95+**	96.60 (73.09 to 125.31)	1772.40 (1340.98 to 2299.15)	21.28 (16.20 to 27.88)	1407.51 (1071.37 to 1843.71)	75.32 (57.07 to 97.69)	1912.49 (1449.00 to 2480.41)

Abbreviations: UI, uncertainty interval.

Table S9. Global count and rates (per 100,000 population) of DALYs from age-related macular degeneration by age group and sex, 2021

	DALYs					
	Overall (95% UI)		Male (95% UI)		Female (95% UI)	
	Count, thousands	Rate (per 100,000)	Count, thousands	Rate (per 100,000)	Count, thousands	Rate (per 100,000)
**All ages**	**578.02 (401.24 to 797.57)**	**7.32 (5.08 to 10.11)**	**232.61 (159.81 to 323.23)**	**5.87 (4.04 to 8.16)**	**345.41 (239.41 to 473.69)**	**8.78 (6.09 to 12.05)**
**Age, years**						
**40–44**	0.00 (0.00 to 0.00)	0.00 (0.00 to 0.00)	0.00 (0.00 to 0.00)	0.00 (0.00 to 0.00)	0.00 (0.00 to 0.00)	0.00 (0.00 to 0.00)
**45–49**	2.08 (1.03 to 3.84)	0.44 (0.22 to 0.81)	0.96 (0.47 to 1.76)	0.40 (0.20 to 0.74)	1.12 (0.57 to 2.08)	0.48 (0.24 to 0.88)
**50–54**	16.67 (9.38 to 26.80)	3.75 (2.11 to 6.02)	7.60 (4.22 to 12.15)	3.43 (1.90 to 5.47)	9.07 (5.11 to 14.54)	4.07 (2.29 to 6.52)
**55–59**	46.52 (29.03 to 69.92)	11.76 (7.34 to 17.67)	20.81 (13.00 to 31.41)	10.69 (6.67 to 16.13)	25.71 (16.03 to 38.57)	12.79 (7.97 to 19.19)
**60–64**	76.60 (50.17 to 113.58)	23.93 (15.68 to 35.49)	33.87 (21.93 to 50.21)	21.78 (14.10 to 32.28)	42.73 (27.90 to 63.54)	25.97 (16.96 to 38.62)
**65–69**	102.05 (67.07 to 147.82)	36.99 (24.31 to 53.59)	44.14 (29.17 to 64.62)	33.48 (22.13 to 49.01)	57.90 (38.08 to 84.22)	40.21 (26.44 to 58.48)
**70–74**	100.49 (68.19 to 142.41)	48.82 (33.13 to 69.18)	42.57 (28.72 to 60.39)	44.17 (29.79 to 62.66)	57.92 (39.38 to 82.04)	52.92 (35.98 to 74.96)
**75–79**	83.93 (57.88 to 117.64)	63.64 (43.89 to 89.20)	34.23 (23.65 to 48.02)	57.25 (39.55 to 80.32)	49.70 (33.99 to 69.86)	68.93 (47.14 to 96.89)
**80–84**	69.96 (46.76 to 98.84)	79.88 (53.39 to 112.86)	25.68 (17.02 to 36.31)	70.05 (46.44 to 99.07)	44.28 (29.73 to 62.75)	86.95 (58.38 to 123.20)
**85–89**	47.43 (31.17 to 66.50)	103.73 (68.18 to 145.44)	14.98 (9.89 to 20.91)	86.82 (57.32 to 121.22)	32.45 (21.32 to 45.52)	113.98 (74.88 to 159.89)
**90–94**	23.09 (15.43 to 32.98)	129.06 (86.27 to 184.35)	5.90 (3.90 to 8.54)	101.31 (66.93 to 146.59)	17.18 (11.48 to 24.45)	142.47 (95.21 to 202.74)
**95+**	9.21 (6.00 to 13.72)	168.99 (110.02 to 251.71)	1.86 (1.18 to 2.82)	122.95 (78.22 to 186.61)	7.35 (4.79 to 10.90)	186.67 (121.64 to 276.76)

Abbreviations: DALYs, disability-adjusted life-years; UI, uncertainty interval.

Table S10. Total count of prevalent cases (million) for age-related macular degeneration for both sexes at the Socio-demographic Index, 1990–2021

	Prevalence				
**Year**	**High SDI (95% UI)**	**High-middle SDI (95% UI)**	**Middle SDI (95% UI)**	**Low-middle SDI (95% UI)**	**Low SDI (95% UI)**
**1990**	0.64 (0.54 to 0.76)	0.92 (0.77 to 1.09)	1.04 (0.86 to 1.28)	0.75 (0.61 to 0.91)	0.29 (0.24 to 0.34)
**1991**	0.65 (0.54 to 0.77)	0.95 (0.80 to 1.13)	1.09 (0.90 to 1.33)	0.76 (0.63 to 0.92)	0.30 (0.25 to 0.36)
**1992**	0.66 (0.55 to 0.78)	0.98 (0.83 to 1.17)	1.14 (0.94 to 1.39)	0.78 (0.64 to 0.94)	0.31 (0.25 to 0.37)
**1993**	0.67 (0.56 to 0.79)	1.01 (0.85 to 1.20)	1.19 (0.98 to 1.44)	0.79 (0.65 to 0.95)	0.32 (0.26 to 0.38)
**1994**	0.67 (0.57 to 0.80)	1.04 (0.88 to 1.23)	1.23 (1.01 to 1.49)	0.80 (0.66 to 0.97)	0.33 (0.27 to 0.39)
**1995**	0.68 (0.57 to 0.81)	1.07 (0.90 to 1.26)	1.26 (1.04 to 1.53)	0.81 (0.67 to 0.98)	0.33 (0.27 to 0.40)
**1996**	0.69 (0.58 to 0.82)	1.09 (0.93 to 1.30)	1.29 (1.06 to 1.56)	0.82 (0.67 to 0.99)	0.34 (0.28 to 0.40)
**1997**	0.70 (0.59 to 0.83)	1.12 (0.95 to 1.33)	1.32 (1.09 to 1.60)	0.82 (0.68 to 0.99)	0.34 (0.28 to 0.40)
**1998**	0.71 (0.59 to 0.84)	1.14 (0.97 to 1.36)	1.35 (1.11 to 1.63)	0.82 (0.68 to 0.99)	0.34 (0.28 to 0.41)
**1999**	0.72 (0.60 to 0.85)	1.17 (0.99 to 1.39)	1.38 (1.13 to 1.67)	0.83 (0.68 to 1.00)	0.34 (0.28 to 0.41)
**2000**	0.73 (0.61 to 0.86)	1.20 (1.02 to 1.43)	1.42 (1.17 to 1.72)	0.84 (0.69 to 1.01)	0.35 (0.29 to 0.42)
**2001**	0.74 (0.62 to 0.88)	1.24 (1.05 to 1.47)	1.47 (1.21 to 1.78)	0.86 (0.71 to 1.03)	0.36 (0.29 to 0.43)
**2002**	0.75 (0.63 to 0.89)	1.28 (1.09 to 1.52)	1.52 (1.26 to 1.85)	0.88 (0.72 to 1.06)	0.37 (0.30 to 0.44)
**2003**	0.76 (0.64 to 0.90)	1.33 (1.12 to 1.57)	1.58 (1.31 to 1.91)	0.89 (0.74 to 1.08)	0.38 (0.31 to 0.45)
**2004**	0.77 (0.65 to 0.92)	1.37 (1.16 to 1.62)	1.64 (1.37 to 1.98)	0.91 (0.75 to 1.10)	0.39 (0.32 to 0.47)
**2005**	0.79 (0.66 to 0.94)	1.41 (1.19 to 1.67)	1.69 (1.41 to 2.04)	0.93 (0.77 to 1.12)	0.40 (0.33 to 0.48)
**2006**	0.80 (0.67 to 0.95)	1.44 (1.21 to 1.71)	1.74 (1.45 to 2.10)	0.95 (0.78 to 1.14)	0.41 (0.34 to 0.49)
**2007**	0.82 (0.69 to 0.97)	1.46 (1.24 to 1.74)	1.78 (1.48 to 2.15)	0.96 (0.79 to 1.16)	0.42 (0.35 to 0.51)
**2008**	0.83 (0.70 to 0.99)	1.49 (1.25 to 1.76)	1.82 (1.51 to 2.19)	0.97 (0.80 to 1.17)	0.43 (0.36 to 0.52)
**2009**	0.85 (0.71 to 1.01)	1.51 (1.28 to 1.80)	1.86 (1.55 to 2.24)	0.99 (0.81 to 1.19)	0.44 (0.36 to 0.54)
**2010**	0.87 (0.73 to 1.03)	1.55 (1.30 to 1.84)	1.91 (1.59 to 2.31)	1.01 (0.83 to 1.22)	0.46 (0.37 to 0.55)
**2011**	0.89 (0.74 to 1.06)	1.59 (1.34 to 1.89)	1.98 (1.64 to 2.38)	1.03 (0.85 to 1.25)	0.47 (0.38 to 0.57)
**2012**	0.91 (0.76 to 1.08)	1.64 (1.38 to 1.94)	2.04 (1.69 to 2.46)	1.05 (0.87 to 1.28)	0.48 (0.39 to 0.58)
**2013**	0.93 (0.78 to 1.11)	1.69 (1.42 to 2.00)	2.11 (1.75 to 2.54)	1.07 (0.88 to 1.30)	0.49 (0.40 to 0.60)
**2014**	0.95 (0.80 to 1.13)	1.74 (1.47 to 2.06)	2.18 (1.81 to 2.63)	1.09 (0.90 to 1.33)	0.50 (0.41 to 0.61)
**2015**	0.98 (0.82 to 1.16)	1.79 (1.51 to 2.12)	2.26 (1.86 to 2.72)	1.12 (0.92 to 1.37)	0.51 (0.42 to 0.63)
**2016**	1.00 (0.84 to 1.19)	1.84 (1.55 to 2.19)	2.34 (1.93 to 2.83)	1.17 (0.95 to 1.43)	0.53 (0.43 to 0.64)

**2017**	1.03 (0.86 to 1.22)	1.89 (1.58 to 2.26)	2.43 (2.00 to 2.95)	1.22 (1.00 to 1.50)	0.55 (0.45 to 0.67)
**2018**	1.05 (0.88 to 1.26)	1.94 (1.61 to 2.33)	2.52 (2.08 to 3.09)	1.29 (1.05 to 1.59)	0.57 (0.47 to 0.70)
**2019**	1.08 (0.91 to 1.29)	2.00 (1.66 to 2.41)	2.63 (2.16 to 3.25)	1.34 (1.09 to 1.66)	0.59 (0.48 to 0.72)
**2020**	1.12 (0.93 to 1.33)	2.12 (1.76 to 2.56)	2.80 (2.31 to 3.42)	1.39 (1.13 to 1.71)	0.61 (0.50 to 0.75)
**2021**	1.13 (0.95 to 1.35)	2.10 (1.76 to 2.56)	2.79 (2.30 to 3.42)	1.40 (1.14 to 1.73)	0.62 (0.51 to 0.77)

Abbreviations: SDI, Socio-demographic Index; UI, uncertainty interval.

Table S11. Total count of DALYs case (thousand) for age-related macular degeneration for both sexes at the Socio-demographic Index, 1990–2021

	DALYs				
**Year**	**High SDI (95% UI)**	**High-middle SDI (95% UI)**	**Middle SDI (95% UI)**	**Low-middle SDI (95% UI)**	**Low SDI (95% UI)**
**1990**	61.28 (41.09 to 82.51)	77.00 (52.26 to 105.77)	78.55 (53.55 to 110.86)	62.34 (42.17 to 88.17)	23.49 (16.02 to 32.69)
**1991**	62.05 (41.78 to 83.87)	81.41 (55.30 to 111.98)	85.15 (57.50 to 120.78)	63.30 (42.79 to 89.62)	24.30 (16.72 to 33.97)
**1992**	62.73 (42.18 to 85.01)	85.31 (58.14 to 117.48)	91.14 (61.44 to 129.14)	64.15 (43.49 to 90.81)	25.05 (17.12 to 35.00)
**1993**	63.40 (42.53 to 85.77)	88.73 (60.45 to 121.74)	96.37 (65.00 to 136.80)	65.01 (43.97 to 91.62)	25.74 (17.70 to 36.01)
**1994**	64.04 (43.15 to 86.56)	91.59 (62.73 to 126.11)	100.67 (67.80 to 142.51)	65.80 (44.90 to 92.68)	26.29 (18.13 to 36.62)
**1995**	64.70 (43.88 to 87.34)	93.87 (64.38 to 129.02)	103.85 (70.18 to 147.50)	66.65 (45.24 to 94.00)	26.69 (18.31 to 37.44)
**1996**	65.19 (44.11 to 88.04)	95.34 (65.54 to 130.49)	105.32 (70.83 to 149.19)	67.14 (45.67 to 95.15)	26.89 (18.45 to 37.73)
**1997**	65.58 (44.50 to 88.68)	95.76 (66.03 to 130.98)	105.28 (70.51 to 148.70)	67.12 (45.78 to 94.22)	26.89 (18.57 to 37.46)
**1998**	65.91 (44.83 to 88.73)	95.79 (66.12 to 130.56)	104.59 (71.10 to 147.30)	66.93 (45.82 to 93.42)	26.80 (18.32 to 37.42)
**1999**	66.36 (45.15 to 89.20)	96.10 (66.64 to 131.06)	104.45 (71.22 to 147.09)	67.02 (45.64 to 94.13)	26.83 (18.37 to 37.07)
**2000**	67.00 (45.43 to 89.95)	97.20 (67.61 to 132.29)	105.87 (72.09 to 149.21)	67.81 (46.69 to 95.06)	27.13 (18.71 to 37.85)
**2001**	67.67 (46.22 to 90.48)	99.41 (69.28 to 134.10)	108.85 (74.47 to 152.39)	69.16 (47.58 to 96.86)	27.72 (19.07 to 38.48)
**2002**	68.25 (46.57 to 92.09)	101.57 (70.39 to 137.64)	112.15 (76.20 to 157.01)	70.47 (48.44 to 98.56)	28.44 (19.53 to 39.47)
**2003**	68.83 (46.87 to 92.28)	103.61 (71.91 to 140.88)	115.52 (78.49 to 162.28)	71.76 (48.59 to 100.80)	29.19 (19.99 to 40.51)
**2004**	69.61 (47.70 to 92.91)	105.79 (73.69 to 143.96)	118.95 (81.30 to 166.02)	72.98 (49.86 to 102.10)	29.98 (20.57 to 41.65)
**2005**	70.67 (48.50 to 95.15)	107.80 (75.54 to 146.70)	122.16 (83.87 to 171.22)	74.20 (50.53 to 104.33)	30.72 (21.25 to 42.63)
**2006**	71.76 (48.84 to 96.58)	110.00 (76.83 to 149.75)	125.20 (85.63 to 175.80)	75.02 (51.27 to 105.19)	31.42 (21.58 to 43.68)
**2007**	72.86 (49.56 to 98.42)	111.76 (78.17 to 151.83)	127.66 (87.71 to 178.24)	75.09 (51.72 to 105.35)	32.07 (21.99 to 45.05)
**2008**	74.01 (50.01 to 99.45)	113.17 (78.86 to 152.58)	129.79 (89.18 to 180.95)	74.89 (51.19 to 104.67)	32.62 (22.39 to 45.73)
**2009**	75.27 (50.84 to 101.44)	115.08 (79.93 to 156.20)	132.38 (90.76 to 184.57)	74.96 (51.57 to 105.24)	33.23 (22.95 to 46.27)
**2010**	76.75 (52.19 to 103.87)	117.16 (81.81 to 157.98)	135.42 (93.16 to 188.78)	75.89 (52.26 to 105.98)	33.99 (23.43 to 47.39)
**2011**	78.09 (53.16 to 104.85)	120.01 (83.73 to 163.28)	139.23 (95.69 to 193.79)	77.46 (53.62 to 108.43)	34.84 (24.04 to 48.52)
**2012**	79.45 (53.84 to 106.81)	122.66 (85.42 to 167.15)	142.91 (98.38 to 198.24)	78.79 (54.36 to 110.73)	35.58 (24.57 to 49.55)
**2013**	80.94 (55.29 to 109.72)	125.16 (87.42 to 169.51)	146.58 (100.60 to 204.74)	80.11 (55.12 to 112.02)	36.31 (24.98 to 50.52)
**2014**	82.53 (56.35 to 111.49)	127.86 (89.05 to 173.88)	150.41 (103.94 to 208.59)	81.62 (56.10 to 114.05)	37.04 (25.55 to 51.83)
**2015**	84.22 (57.44 to 113.09)	130.35 (91.14 to 177.89)	154.16 (106.43 to 214.25)	83.50 (57.30 to 117.08)	37.91 (26.07 to 52.76)
**2016**	85.85 (58.72 to 115.82)	132.22 (92.04 to 180.58)	157.83 (109.22 to 219.37)	86.49 (59.07 to 121.59)	38.97 (26.80 to 54.35)

**2017**	87.58 (60.04 to 118.18)	133.06 (93.19 to 182.22)	161.06 (111.38 to 222.96)	90.32 (61.89 to 126.28)	40.09 (27.60 to 55.82)
**2018**	89.42 (60.61 to 120.91)	133.93 (93.65 to 184.32)	164.81 (113.64 to 228.94)	94.30 (64.35 to 132.43)	41.34 (28.43 to 57.63)
**2019**	91.51 (62.92 to 123.29)	136.55 (95.80 to 188.22)	170.30 (117.58 to 234.85)	97.90 (66.86 to 137.80)	42.65 (29.31 to 59.22)
**2020**	93.84 (64.16 to 127.35)	143.09 (99.73 to 195.25)	180.62 (125.08 to 248.66)	101.09 (69.29 to 141.77)	43.79 (30.06 to 60.93)
**2021**	95.84 (65.15 to 129.96)	147.38 (102.64 to 202.09)	187.90 (129.23 to 259.07)	101.90 (70.14 to 143.94)	44.62 (30.57 to 61.77)

Abbreviations: DALYs, disability-adjusted life-years; SDI, Socio-demographic Index; UI, uncertainty interval.

Table S12. Total age-standardized rate (per 100,000 population) of prevalence for age-related macular degeneration for both sexes at the Socio-demographic Index, 1990–2021

	Prevalence				
**Year**	**High SDI (95% UI)**	**High-middle SDI (95% UI)**	**Middle SDI (95% UI)**	**Low-middle SDI (95% UI)**	**Low SDI (95% UI)**
**1990**	56.98 (47.61 to 67.51)	100.42 (84.93 to 118.77)	114.58 (94.89 to 138.84)	138.32 (114.13 to 165.93)	144.70 (120.56 to 171.58)
**1991**	56.53 (47.23 to 66.87)	101.34 (85.81 to 119.45)	116.50 (96.64 to 141.02)	137.01 (113.13 to 164.24)	146.76 (122.22 to 173.89)
**1992**	56.06 (46.86 to 66.29)	102.20 (86.77 to 120.14)	118.04 (98.04 to 142.83)	135.51 (112.06 to 162.33)	148.40 (123.51 to 175.70)
**1993**	55.59 (46.50 to 65.70)	102.99 (87.53 to 120.84)	119.14 (98.95 to 143.86)	133.91 (110.82 to 160.54)	149.52 (124.36 to 176.94)
**1994**	55.17 (46.21 to 65.19)	103.60 (88.05 to 121.62)	119.72 (99.34 to 144.37)	132.26 (109.50 to 158.82)	150.08 (124.75 to 177.81)
**1995**	54.74 (45.93 to 64.69)	104.04 (88.49 to 122.25)	119.70 (99.32 to 144.19)	130.61 (108.21 to 157.12)	149.93 (124.58 to 178.00)
**1996**	54.28 (45.52 to 64.14)	104.06 (88.45 to 122.21)	118.94 (98.68 to 143.39)	128.44 (106.61 to 154.32)	148.65 (123.65 to 176.24)
**1997**	53.75 (45.06 to 63.50)	103.83 (88.21 to 122.06)	117.65 (97.60 to 141.75)	125.56 (104.27 to 150.60)	146.44 (121.93 to 173.85)
**1998**	53.22 (44.59 to 62.90)	103.59 (87.98 to 122.09)	116.28 (96.59 to 139.77)	122.56 (101.97 to 146.75)	143.99 (120.06 to 171.16)
**1999**	52.77 (44.22 to 62.50)	103.53 (87.85 to 122.35)	115.26 (95.76 to 138.32)	120.03 (99.90 to 143.53)	141.95 (118.51 to 168.64)
**2000**	52.38 (43.99 to 62.16)	104.01 (88.15 to 122.88)	115.09 (95.87 to 138.08)	118.53 (98.58 to 141.75)	140.94 (117.61 to 167.42)
**2001**	52.01 (43.66 to 61.75)	105.05 (89.05 to 124.23)	115.68 (96.22 to 138.62)	117.69 (97.89 to 140.84)	141.00 (117.62 to 167.80)
**2002**	51.54 (43.30 to 61.25)	106.34 (90.22 to 125.69)	116.52 (96.84 to 139.47)	116.79 (97.13 to 139.98)	141.55 (117.95 to 168.80)
**2003**	51.05 (42.94 to 60.72)	107.74 (91.46 to 127.24)	117.34 (97.40 to 140.30)	115.86 (96.31 to 138.99)	142.32 (118.41 to 170.12)
**2004**	50.65 (42.65 to 60.27)	108.88 (92.53 to 128.51)	117.88 (97.87 to 140.79)	114.94 (95.50 to 138.10)	142.99 (119.00 to 171.37)
**2005**	50.31 (42.40 to 59.88)	109.45 (93.09 to 129.07)	117.94 (98.11 to 140.69)	114.06 (94.64 to 137.24)	143.30 (119.22 to 172.16)
**2006**	49.99 (42.15 to 59.57)	109.29 (92.99 to 128.75)	117.14 (97.27 to 139.95)	112.73 (93.57 to 135.60)	143.09 (119.13 to 172.11)
**2007**	49.60 (41.78 to 59.15)	108.27 (92.23 to 127.61)	115.67 (96.07 to 138.51)	110.78 (91.97 to 133.55)	142.81 (118.87 to 171.98)
**2008**	49.22 (41.42 to 58.70)	107.05 (91.21 to 126.40)	113.92 (94.68 to 136.76)	108.66 (90.17 to 131.33)	142.49 (118.45 to 171.78)
**2009**	48.93 (41.14 to 58.35)	106.04 (90.23 to 125.46)	112.37 (93.45 to 135.22)	106.80 (88.50 to 129.35)	142.14 (118.04 to 171.38)
**2010**	48.70 (40.90 to 58.07)	105.60 (89.69 to 125.18)	111.50 (92.75 to 134.46)	105.63 (87.39 to 128.05)	142.07 (117.70 to 171.61)
**2011**	48.54 (40.79 to 57.89)	105.77 (89.65 to 125.30)	111.03 (92.28 to 133.93)	104.63 (86.55 to 127.06)	141.48 (117.21 to 170.60)
**2012**	48.40 (40.69 to 57.76)	105.74 (89.30 to 125.17)	110.49 (91.81 to 133.31)	103.30 (85.42 to 125.56)	140.41 (116.28 to 169.28)
**2013**	48.30 (40.63 to 57.72)	105.74 (89.12 to 125.10)	109.91 (91.34 to 132.65)	101.95 (84.15 to 124.02)	139.26 (115.21 to 167.90)
**2014**	48.28 (40.63 to 57.77)	105.83 (89.06 to 125.16)	109.37 (90.90 to 132.00)	100.89 (83.07 to 122.81)	138.32 (114.23 to 166.80)
**2015**	48.25 (40.61 to 57.78)	105.81 (89.06 to 125.32)	108.87 (90.39 to 131.26)	100.40 (82.50 to 122.29)	137.92 (113.81 to 166.47)
**2016**	48.23 (40.62 to 57.84)	105.63 (88.83 to 125.54)	108.49 (89.81 to 131.54)	101.07 (82.98 to 123.00)	137.95 (114.14 to 166.65)

**2017**	48.20 (40.61 to 57.68)	105.07 (87.86 to 125.39)	108.31 (89.66 to 132.10)	102.77 (84.24 to 125.27)	138.39 (114.62 to 167.61)
**2018**	48.22 (40.62 to 57.60)	104.68 (87.10 to 125.28)	108.33 (89.62 to 132.94)	104.56 (85.55 to 127.74)	138.91 (114.72 to 168.67)
**2019**	48.33 (40.70 to 57.66)	104.91 (87.11 to 126.17)	108.59 (89.52 to 133.95)	105.51 (86.14 to 129.09)	139.15 (114.93 to 169.10)
**2020**	48.79 (40.78 to 58.35)	108.13 (89.89 to 130.29)	111.27 (91.73 to 135.87)	105.97 (86.94 to 130.05)	139.58 (115.51 to 169.92)
**2021**	48.43 (40.55 to 57.77)	104.55 (87.88 to 126.60)	107.56 (88.68 to 131.24)	104.67 (85.46 to 127.94)	139.92 (114.54 to 171.01)

Abbreviations: SDI, Socio-demographic Index; UI, uncertainty interval.

Table S13. Total age-standardized rate (per 100,000 population) of DALYs for age-related macular degeneration for both sexes at the Socio-demographic Index, 1990–2021

	DALYs				
**Year**	**High SDI (95% UI)**	**High-middle SDI (95% UI)**	**Middle SDI (95% UI)**	**Low-middle SDI (95% UI)**	**Low SDI (95% UI)**
**1990**	5.48 (3.68 to 7.35)	8.65 (5.88 to 11.82)	8.65 (5.85 to 12.13)	11.43 (7.79 to 16.04)	11.90 (8.19 to 16.58)
**1991**	5.43 (3.67 to 7.29)	8.88 (6.03 to 12.11)	9.08 (6.12 to 12.74)	11.30 (7.74 to 15.84)	12.03 (8.29 to 16.61)
**1992**	5.37 (3.62 to 7.26)	9.07 (6.17 to 12.39)	9.42 (6.38 to 13.23)	11.14 (7.65 to 15.68)	12.11 (8.39 to 16.81)
**1993**	5.32 (3.57 to 7.18)	9.21 (6.25 to 12.59)	9.67 (6.56 to 13.55)	10.99 (7.52 to 15.32)	12.15 (8.39 to 16.80)
**1994**	5.26 (3.56 to 7.10)	9.29 (6.34 to 12.70)	9.81 (6.67 to 13.73)	10.83 (7.43 to 15.09)	12.15 (8.42 to 16.76)
**1995**	5.20 (3.54 to 7.04)	9.33 (6.39 to 12.73)	9.83 (6.68 to 13.82)	10.70 (7.35 to 14.97)	12.10 (8.39 to 16.80)
**1996**	5.14 (3.47 to 6.94)	9.26 (6.34 to 12.65)	9.69 (6.59 to 13.57)	10.51 (7.24 to 14.71)	11.95 (8.26 to 16.62)
**1997**	5.06 (3.44 to 6.83)	9.09 (6.25 to 12.37)	9.40 (6.39 to 13.14)	10.25 (7.06 to 14.19)	11.70 (8.13 to 16.18)
**1998**	4.97 (3.38 to 6.69)	8.90 (6.12 to 12.13)	9.05 (6.16 to 12.68)	9.97 (6.88 to 13.80)	11.41 (7.84 to 15.77)
**1999**	4.90 (3.33 to 6.59)	8.72 (6.02 to 11.81)	8.77 (5.96 to 12.22)	9.73 (6.70 to 13.57)	11.18 (7.69 to 15.45)
**2000**	4.84 (3.28 to 6.49)	8.64 (5.99 to 11.77)	8.62 (5.91 to 12.05)	9.58 (6.64 to 13.29)	11.06 (7.57 to 15.35)
**2001**	4.78 (3.27 to 6.42)	8.64 (5.99 to 11.63)	8.60 (5.91 to 11.93)	9.50 (6.61 to 13.21)	11.04 (7.55 to 15.30)
**2002**	4.71 (3.22 to 6.31)	8.63 (5.95 to 11.70)	8.60 (5.94 to 11.97)	9.40 (6.50 to 13.08)	11.05 (7.64 to 15.27)
**2003**	4.63 (3.16 to 6.21)	8.62 (5.95 to 11.65)	8.59 (5.90 to 11.94)	9.31 (6.39 to 12.97)	11.07 (7.62 to 15.25)
**2004**	4.57 (3.13 to 6.12)	8.60 (5.96 to 11.69)	8.56 (5.92 to 11.90)	9.20 (6.35 to 12.79)	11.09 (7.68 to 15.32)
**2005**	4.52 (3.11 to 6.07)	8.56 (5.98 to 11.58)	8.52 (5.87 to 11.86)	9.09 (6.28 to 12.68)	11.08 (7.70 to 15.34)
**2006**	4.48 (3.05 to 6.03)	8.52 (5.92 to 11.52)	8.44 (5.82 to 11.72)	8.91 (6.13 to 12.47)	11.01 (7.59 to 15.23)
**2007**	4.42 (3.02 to 5.95)	8.41 (5.87 to 11.39)	8.30 (5.74 to 11.50)	8.65 (6.00 to 12.12)	10.92 (7.53 to 15.21)
**2008**	4.37 (2.97 to 5.87)	8.29 (5.75 to 11.18)	8.15 (5.64 to 11.25)	8.38 (5.77 to 11.67)	10.79 (7.47 to 15.03)
**2009**	4.33 (2.94 to 5.82)	8.19 (5.67 to 11.07)	8.01 (5.56 to 11.10)	8.13 (5.64 to 11.35)	10.67 (7.34 to 14.76)
**2010**	4.30 (2.93 to 5.79)	8.11 (5.65 to 10.96)	7.91 (5.48 to 10.98)	7.99 (5.54 to 11.10)	10.62 (7.35 to 14.68)
**2011**	4.26 (2.90 to 5.73)	8.06 (5.60 to 10.93)	7.83 (5.45 to 10.85)	7.89 (5.49 to 11.00)	10.55 (7.29 to 14.60)
**2012**	4.22 (2.86 to 5.64)	7.99 (5.55 to 10.83)	7.74 (5.39 to 10.70)	7.77 (5.39 to 10.89)	10.46 (7.24 to 14.51)
**2013**	4.19 (2.85 to 5.65)	7.92 (5.52 to 10.69)	7.65 (5.30 to 10.59)	7.66 (5.29 to 10.67)	10.37 (7.13 to 14.31)
**2014**	4.16 (2.83 to 5.60)	7.84 (5.46 to 10.64)	7.54 (5.26 to 10.42)	7.57 (5.22 to 10.50)	10.29 (7.08 to 14.29)
**2015**	4.13 (2.82 to 5.56)	7.77 (5.43 to 10.60)	7.45 (5.19 to 10.29)	7.51 (5.14 to 10.50)	10.25 (7.03 to 14.24)
**2016**	4.11 (2.80 to 5.54)	7.65 (5.32 to 10.37)	7.33 (5.10 to 10.12)	7.53 (5.19 to 10.55)	10.22 (7.04 to 14.19)

**2017**	4.08 (2.78 to 5.52)	7.47 (5.23 to 10.23)	7.20 (5.02 to 9.93)	7.61 (5.24 to 10.58)	10.19 (7.00 to 14.10)
**2018**	4.07 (2.74 to 5.51)	7.30 (5.11 to 10.05)	7.09 (4.96 to 9.73)	7.69 (5.26 to 10.72)	10.17 (7.00 to 14.08)
**2019**	4.06 (2.77 to 5.46)	7.22 (5.07 to 9.96)	7.04 (4.90 to 9.64)	7.73 (5.29 to 10.82)	10.15 (7.00 to 14.00)
**2020**	4.07 (2.77 to 5.50)	7.36 (5.14 to 10.04)	7.20 (5.05 to 9.90)	7.76 (5.34 to 10.78)	10.12 (6.96 to 14.01)
**2021**	4.08 (2.76 to 5.49)	7.40 (5.15 to 10.14)	7.26 (5.00 to 9.96)	7.64 (5.28 to 10.75)	10.08 (6.91 to 13.86)

Abbreviations: DALYs, disability-adjusted life-years; SDI, Socio-demographic Index; UI, uncertainty interval.

Table S14. Total count of DALYs attributed to risk factor (tobacco) of age-related macular degeneration by region and sex, 1990 and 2021

	Counts, thousands					
	1990 (95% UI)			2021 (95% UI)		
	Both	Male	Female	Both	Male	Female
**Global**	40.01 (21.92 to 65.05)	28.75 (16.02 to 46.06)	11.26 (5.87 to 19.05)	58.86 (31.94 to 96.95)	45.44 (24.71 to 74.18)	13.42 (6.91 to 22.73)
**SDI regions**						
High SDI	8.33 (4.52 to 13.73)	4.23 (2.34 to 6.90)	4.10 (2.11 to 6.90)	8.77 (4.69 to 14.79)	5.04 (2.67 to 8.54)	3.73 (1.93 to 6.21)
High-middle SDI	11.01 (6.07 to 17.78)	7.67 (4.30 to 12.22)	3.35 (1.75 to 5.58)	17.83 (9.70 to 28.88)	13.62 (7.64 to 22.03)	4.21 (2.05 to 7.10)
Middle SDI	10.86 (5.97 to 17.68)	8.77 (4.90 to 14.24)	2.08 (1.06 to 3.56)	19.64 (10.63 to 32.02)	16.50 (9.07 to 26.93)	3.14 (1.60 to 5.35)
Low-middle SDI	7.82 (4.31 to 12.56)	6.56 (3.66 to 10.54)	1.26 (0.65 to 2.14)	9.77 (5.36 to 16.20)	8.21 (4.49 to 13.49)	1.56 (0.83 to 2.71)
Low SDI	1.95 (1.06 to 3.17)	1.49 (0.81 to 2.41)	0.45 (0.24 to 0.75)	2.82 (1.51 to 4.72)	2.04 (1.11 to 3.41)	0.77 (0.40 to 1.33)
**Central Europe, Eastern Europe, and Central Asia**						
Central Asia	0.14 (0.08 to 0.23)	0.12 (0.06 to 0.19)	0.02 (0.01 to 0.04)	0.23 (0.13 to 0.40)	0.20 (0.11 to 0.33)	0.04 (0.02 to 0.07)
Central Europe	1.03 (0.55 to 1.70)	0.66 (0.36 to 1.07)	0.38 (0.19 to 0.63)	1.04 (0.56 to 1.76)	0.66 (0.36 to 1.10)	0.38 (0.20 to 0.65)
Eastern Europe	0.55 (0.27 to 0.89)	0.47 (0.23 to 0.75)	0.08 (0.04 to 0.15)	0.60 (0.30 to 1.01)	0.49 (0.25 to 0.81)	0.11 (0.06 to 0.20)
**High-income regions**						
Australasia	0.12 (0.06 to 0.21)	0.05 (0.02 to 0.08)	0.07 (0.04 to 0.13)	0.14 (0.07 to 0.25)	0.05 (0.03 to 0.10)	0.09 (0.04 to 0.15)
High-income Asian Pacific	0.73 (0.38 to 1.18)	0.55 (0.29 to 0.89)	0.18 (0.09 to 0.31)	0.88 (0.44 to 1.47)	0.67 (0.35 to 1.12)	0.20 (0.10 to 0.36)
High-income North America	1.51 (0.77 to 2.59)	0.57 (0.30 to 0.99)	0.93 (0.48 to 1.62)	1.71 (0.87 to 3.04)	0.75 (0.37 to 1.33)	0.96 (0.48 to 1.71)
Southern Latin America	0.17 (0.09 to 0.29)	0.10 (0.05 to 0.16)	0.07 (0.04 to 0.13)	0.19 (0.09 to 0.32)	0.10 (0.05 to 0.18)	0.08 (0.04 to 0.14)
Western Europe	9.06 (4.81 to 14.78)	4.77 (2.60 to 7.75)	4.29 (2.20 to 7.19)	7.41 (3.85 to 12.45)	4.07 (2.10 to 7.03)	3.34 (1.69 to 5.54)
**Latin America and Caribbean**						
Andean Latin America	0.08 (0.04 to 0.14)	0.06 (0.03 to 0.11)	0.02 (0.01 to 0.03)	0.20 (0.10 to 0.35)	0.15 (0.08 to 0.26)	0.05 (0.02 to 0.09)
Caribbean	0.07 (0.03 to 0.11)	0.04 (0.02 to 0.08)	0.02 (0.01 to 0.04)	0.09 (0.05 to 0.15)	0.06 (0.03 to 0.10)	0.03 (0.02 to 0.05)
Central Latin America	0.36 (0.20 to 0.60)	0.26 (0.14 to 0.44)	0.10 (0.05 to 0.17)	0.49 (0.26 to 0.83)	0.35 (0.18 to 0.61)	0.13 (0.07 to 0.23)
Tropical Latin America	0.67 (0.36 to 1.11)	0.33 (0.18 to 0.55)	0.34 (0.18 to 0.58)	1.07 (0.55 to 1.88)	0.47 (0.23 to 0.81)	0.60 (0.31 to 1.08)
**North Africa and Middle East**	2.93 (1.61 to 4.85)	2.48 (1.38 to 4.08)	0.45 (0.23 to 0.78)	5.61 (3.06 to 9.46)	4.76 (2.63 to 7.99)	0.85 (0.42 to 1.50)

**South Asia**	8.93 (4.96 to 14.52)	2.13 (1.19 to 3.44)	0.47 (0.24 to 0.80)	3.84 (2.04 to 6.31)	3.30 (1.77 to 5.36)	0.54 (0.27 to 0.92)
**Southeast Asia, East Asia, and Oceania**						
East Asia	9.97 (5.48 to 16.34)	7.90 (4.42 to 12.80)	2.07 (1.08 to 3.64)	23.25 (12.94 to 37.74)	19.60 (11.09 to 31.75)	3.65 (1.80 to 6.45)
Oceania	0.01 (0.01 to 0.02)					
Southeast Asia	2.60 (1.44 to 4.21)	2.13 (1.19 to 3.44)	0.47 (0.24 to 0.80)	3.84 (2.04 to 6.31)	3.30 (1.77 to 5.36)	0.54 (0.27 to 0.92)
**Sub-Saharan Africa**						
Central Sub-Saharan Africa	0.02 (0.01 to 0.03)	0.01 (0.01 to 0.02)	0.002 (0.001 to 0.004)	0.03 (0.02 to 0.05)	0.03 (0.01 to 0.05)	0.005 (0.002 to 0.009)
Eastern Sub-Saharan Africa	0.58 (0.31 to 0.95)	0.45 (0.24 to 0.73)	0.13 (0.07 to 0.23)	0.69 (0.37 to 1.15)	0.53 (0.28 to 0.89)	0.16 (0.08 to 0.26)
Southern Sub-Saharan Africa	0.10 (0.05 to 0.16)	0.06 (0.03 to 0.09)	0.04 (0.02 to 0.07)	0.11 (0.06 to 0.18)	0.06 (0.04 to 0.11)	0.04 (0.02 to 0.08)
Western Sub-Saharan Africa	0.39 (0.21 to 0.67)	0.31 (0.16 to 0.53)	0.08 (0.04 to 0.14)	0.72 (0.37 to 1.27)	0.59 (0.30 to 1.03)	0.13 (0.07 to 0.23)

Abbreviations: DALYs, disability-adjusted life-years; SDI, Socio-demographic Index; UI, uncertainty interval.

Table S15. Age-standardized percentage and percentage change of DALYs attributed to risk factor (tobacco) of age-related macular degeneration by region and sex, 1990–2021

	Percentage (%)								
	1990 (95% UI)			2021 (95% UI)			Change between 1990 to 2021 (95% UI)		
	Both	Male	Female	Both	Male	Female	Both	Male	Female
**Global**	12.45 (7.73 to 17.37)	22.61 (14.29 to 30.61)	6.02 (3.47 to 8.73)	9.96 (6.12 to 14.06)	18.72 (11.69 to 26.02)	3.89 (2.20 to 5.83)	-20.03 (-23.43 to -17.19)	-17.21 (-20.36 to -14.35)	-35.35 (-40.34 to -29.79)
**SDI regions**									
High SDI	13.62 (8.10 to 19.55)	21.72 (13.16 to 30.58)	10.14 (5.92 to 14.71)	10.00 (5.80 to 14.63)	14.74 (8.68 to 21.40)	6.99 (3.96 to 10.42)	-26.58 (-31.26 to -21.91)	-32.15 (-36.89 to -27.47)	-31.07 (-36.48 to -24.94)
High-middle SDI	13.07 (8.14 to 17.97)	26.08 (16.59 to 35.50)	6.42 (3.71 to 9.38)	11.81 (7.38 to 16.38)	23.06 (14.70 to 31.80)	4.67 (2.67 to 6.82)	-9.67 (-15.60 to -4.25)	-11.57 (-15.80 to -7.24)	-27.19 (-37.79 to -15.09)
Middle SDI	12.85 (8.14 to 17.57)	24.53 (15.77 to 32.78)	4.43 (2.53 to 6.53)	10.03 (6.24 to 14.02)	20.55 (12.95 to 28.28)	2.76 (1.53 to 4.15)	-21.97 (-26.52 to -17.75)	-16.22 (-20.79 to -11.73)	-37.84 (-45.77 to -28.27)
Low-middle SDI	11.92 (7.40 to 16.44)	20.89 (13.11 to 28.40)	3.68 (2.05 to 5.47)	9.24 (5.67 to 13.17)	17.15 (10.70 to 23.94)	2.71 (1.55 to 4.26)	-22.42 (-27.61 to -17.47)	-17.89 (-23.24 to -13.04)	-26.22 (-38.71 to -10.93)
Low SDI	7.84 (4.74 to 10.96)	12.98 (7.92 to 18.11)	3.50 (2.06 to 5.07)	6.01 (3.56 to 8.60)	9.92 (5.94 to 14.12)	2.98 (1.69 to 4.48)	-23.42 (-29.48 to -17.76)	-23.55 (-29.02 to -18.57)	-14.70 (-28.72 to 1.58)
**Central Europe, Eastern Europe, and Central Asia**									
Central Asia	5.67 (3.43 to 8.13)	20.69 (12.79 to 29.06)	1.23 (0.68 to 1.90)	5.94 (3.58 to 8.40)	20.72 (12.72 to 28.75)	1.22 (0.68 to 1.91)	4.84 (-4.10 to 14.55)	0.15 (-5.53 to 6.78)	-0.79 (-19.72 to 20.43)
Central Europe	14.13 (8.71 to 20.08)	23.23 (14.55 to 32.65)	8.63 (5.04 to 12.70)	11.67 (7.03 to 16.51)	17.10 (10.27 to 24.21)	7.79 (4.65 to 11.35)	-17.43 (-22.45 to -12.23)	-26.40 (-30.87 to -21.89)	-9.80 (-18.27 to -0.38)
Eastern Europe	7.99 (4.89 to 11.38)	23.78 (15.09 to 32.80)	1.78 (0.98 to 2.69)	8.62 (5.23 to 12.23)	21.82 (13.40 to 30.51)	2.54 (1.51 to 3.88)	7.88 (-1.02 to 17.22)	-8.23 (-14.99 to -2.19)	43.21 (16.76 to 79.92)
**High-income regions**									
Australasia	10.55 (5.90 to 15.86)	13.51 (7.79 to 19.80)	9.56 (5.50 to 14.60)	6.91 (3.83 to 10.63)	7.47 (3.87 to 11.84)	6.67 (3.75 to 10.15)	-34.52 (-44.14 to -23.48)	-44.73 (-53.94 to -35.01)	-30.24 (-42.80 to -15.57)
High-income Asian Pacific	14.67 (8.88 to 20.53)	28.28 (18.01 to 38.58)	6.05 (3.29 to 9.02)	9.26 (5.33 to 13.57)	16.47 (9.77 to 24.05)	3.70 (1.96 to 5.84)	-36.86 (-43.57 to -29.55)	-41.78 (-48.77 to -34.86)	-38.83 (-51.01 to -24.03)
High-income North America	13.84 (8.10 to 20.23)	17.77 (10.26 to 25.79)	12.54 (7.37 to 18.39)	9.58 (5.26 to 15.10)	11.68 (6.49 to 18.03)	8.49 (4.55 to 13.37)	-30.74 (-39.70 to -21.45)	-34.27 (-42.08 to -26.81)	-32.28 (-43.70 to -20.52)
Southern Latin America	9.61 (5.72 to 14.26)	12.00 (6.99 to 17.61)	7.66 (4.40 to 11.82)	7.68 (4.37 to 11.35)	9.07 (4.85 to 13.82)	6.45 (3.74 to 9.51)	-20.10 (-30.33 to -7.63)	-24.42 (-36.48 to -10.37)	-15.80 (-33.26 to 8.81)
Western Europe	13.15 (7.83 to 18.71)	23.03 (14.00 to 32.27)	9.31 (5.40 to 13.42)	9.27 (5.39 to 13.68)	14.03 (8.06 to 20.65)	6.73 (3.94 to 9.92)	-29.56 (-34.39 to -24.25)	-39.09 (-44.76 to -33.36)	-27.65 (-33.75 to -21.43)
**Latin America and Caribbean**									

Andean Latin America	4.52 (2.54 to 6.90)	9.26 (5.22 to 14.01)	1.75 (0.94 to 2.82)	4.38 (2.44 to 6.88)	8.81 (4.83 to 13.65)	1.68 (0.88 to 2.72)	-3.07 (-18.50 to 14.58)	-4.80 (-18.88 to 11.27)	-4.47 (-28.26 to 28.14)
Caribbean	11.78 (7.02 to 17.27)	17.02 (10.18 to 24.36)	7.46 (4.26 to 11.33)	9.05 (5.25 to 13.14)	13.01 (7.44 to 18.93)	5.81 (3.28 to 8.90)	-23.24 (-33.17 to -12.71)	-23.57 (-32.86 to -13.00)	-22.20 (-37.93 to -2.96)
Central Latin America	9.44 (5.69 to 13.56)	14.84 (9.03 to 20.98)	4.83 (2.72 to 7.30)	5.08 (2.98 to 7.51)	8.23 (4.79 to 12.19)	2.52 (1.44 to 3.86)	-46.23 (-52.03 to -40.10)	-44.54 (-50.34 to -38.62)	-47.87 (-57.50 to -37.05)
Tropical Latin America	15.88 (9.45 to 22.96)	22.04 (13.89 to 30.82)	12.62 (6.90 to 18.80)	9.30 (5.10 to 14.10)	12.43 (7.01 to 18.89)	7.89 (4.37 to 11.88)	-41.41 (-50.81 to -30.73)	-43.59 (-52.88 to -33.56)	-37.51 (-49.65 to -21.23)
**North Africa and Middle East**	10.82 (6.65 to 15.37)	20.76 (12.76 to 29.22)	2.92 (1.64 to 4.38)	9.17 (5.61 to 13.21)	18.08 (11.04 to 25.71)	2.40 (1.32 to 3.61)	-15.24 (-20.32 to -10.17)	-12.91 (-17.77 to -8.29)	-17.79 (-29.86 to -3.98)
**South Asia**	12.73 (7.82 to 17.61)	21.04 (13.06 to 28.83)	4.27 (2.38 to 6.35)	9.02 (5.45 to 13.10)	15.57 (9.51 to 22.01)	3.27 (1.86 to 5.11)	-29.17 (-35.85 to -22.86)	-26.02 (-33.01 to -19.86)	-23.44 (-39.48 to -3.85)
**Southeast Asia, East Asia, and Oceania**									
East Asia	16.78 (10.77 to 22.39)	32.45 (21.21 to 42.91)	6.35 (3.56 to 9.26)	14.49 (9.10 to 19.54)	29.82 (19.16 to 39.91)	3.95 (2.13 to 6.05)	-13.63 (-21.45 to -5.73)	-8.12 (-14.70 to -0.99)	-37.79 (-53.45 to -17.21)
Oceania	9.86 (5.87 to 14.32)	14.72 (8.83 to 21.01)	6.42 (3.60 to 9.66)	8.68 (4.95 to 12.87)	12.58 (7.35 to 18.24)	6.04 (3.35 to 9.28)	-11.96 (-26.84 to 3.75)	-14.51 (-26.92 to 0.21)	-5.86 (-26.52 to 20.62)
Southeast Asia	13.03 (8.33 to 17.89)	25.49 (16.27 to 34.29)	4.19 (2.46 to 6.19)	10.23 (6.32 to 14.29)	21.16 (13.11 to 29.09)	2.56 (1.50 to 3.85)	-21.49 (-26.82 to -16.18)	-16.98 (-21.77 to -12.36)	-38.97 (-47.91 to -28.71)
**Sub-Saharan Africa**									
Central Sub-Saharan Africa	3.70 (2.15 to 5.53)	7.95 (4.76 to 11.68)	0.78 (0.44 to 1.23)	3.08 (1.76 to 4.70)	7.43 (4.29 to 11.06)	0.72 (0.37 to 1.15)	-16.79 (-29.39 to -1.67)	-6.61 (-19.45 to 8.96)	-7.98 (-30.38 to 21.09)
Eastern Sub-Saharan Africa	5.88 (3.52 to 8.53)	11.23 (6.73 to 16.07)	2.36 (1.43 to 3.55)	3.95 (2.34 to 5.81)	7.73 (4.56 to 11.33)	1.53 (0.88 to 2.29)	-32.76 (-38.79 to -26.49)	-31.18 (-37.35 to -25.25)	-35.15 (-46.21 to -23.28)
Southern Sub-Saharan Africa	11.95 (7.19 to 17.23)	20.65 (12.83 to 28.98)	7.62 (4.48 to 11.43)	5.82 (3.39 to 8.65)	10.64 (6.31 to 15.23)	3.55 (1.94 to 5.65)	-51.34 (-57.64 to -43.79)	-48.46 (-55.35 to -41.24)	-53.42 (-64.25 to -40.27)
Western Sub-Saharan Africa	3.01 (1.70 to 4.53)	5.35 (3.01 to 8.04)	1.20 (0.67 to 1.89)	2.56 (1.46 to 3.89)	5.12 (2.89 to 7.72)	0.80 (0.43 to 1.26)	-14.89 (-26.11 to -2.75)	-4.27 (-16.58 to 9.78)	-33.90 (-50.64 to -10.55)

Abbreviations: DALYs, disability-adjusted life-years; SDI, Socio-demographic Index; UI, uncertainty interval.

Table S16. Forecasted prevalent cases and rates (per 100,000 population) of age-related macular degeneration by sex, comparing estimates with risk factor, all ages, 1990–2050

	Male (95% UI)		Female (95% UI)	
**Year**	**Number, million**	**Rate (per 100,000)**	**Number, million**	**Rate (per 100,000)**
**2021**	**3.40 (2.81 to 4.17)**	**85.86 (58.68 to 125.43)**	**4.66 (3.88 to 5.65)**	**118.46 (91.15 to 153.87)**
2022	4.67 (2.96 to 7.36)	97.28 (61.63 to 153.22)	5.90 (4.25 to 8.19)	114.52 (82.47 to 158.87)
2023	4.80 (3.04 to 7.57)	98.05 (62.12 to 154.43)	6.09 (4.39 to 8.45)	115.69 (83.32 to 160.49)
2024	4.94 (3.13 to 7.78)	98.82 (62.61 to 155.64)	6.28 (4.52 to 8.71)	116.87 (84.17 to 162.12)
2025	5.07 (3.21 to 7.99)	99.60 (63.11 to 156.86)	6.47 (4.66 to 8.98)	118.06 (85.03 to 163.77)
2026	5.21 (3.30 to 8.21)	100.38 (63.61 to 158.09)	6.67 (4.81 to 9.26)	119.26 (85.90 to 165.43)
2027	5.36 (3.39 to 8.44)	101.17 (64.11 to 159.33)	6.88 (4.95 to 9.54)	120.48 (86.78 to 167.11)
2028	5.51 (3.49 to 8.67)	101.97 (64.62 to 160.58)	7.09 (5.11 to 9.84)	121.70 (87.67 to 168.81)
2029	5.66 (3.59 to 8.91)	102.77 (65.13 to 161.84)	7.32 (5.27 to 10.15)	122.94 (88.56 to 170.53)
**2030**	**5.82 (3.69 to 9.16)**	**103.58 (65.65 to 163.11)**	**7.54 (5.44 to 10.46)**	**124.20 (89.47 to 172.26)**
2031	5.98 (3.79 to 9.42)	104.40 (66.17 to 164.39)	7.78 (5.60 to 10.79)	125.46 (90.38 to 174.01)
2032	6.14 (3.89 to 9.67)	105.22 (66.69 to 165.68)	8.01 (5.77 to 11.11)	126.74 (91.30 to 175.78)
2033	6.30 (3.99 to 9.92)	106.05 (67.22 to 166.98)	8.25 (5.95 to 11.45)	128.03 (92.24 to 177.57)
2034	6.47 (4.10 to 10.18)	106.88 (67.75 to 168.29)	8.50 (6.12 to 11.79)	129.33 (93.18 to 179.37)
2035	6.63 (4.21 to 10.45)	107.72 (68.29 to 169.61)	8.75 (6.30 to 12.13)	130.65 (94.13 to 181.19)
2036	6.80 (4.31 to 10.70)	108.57 (68.83 to 170.94)	9.00 (6.48 to 12.48)	131.98 (95.09 to 183.03)
2037	6.96 (4.41 to 10.96)	109.43 (69.37 to 172.28)	9.24 (6.66 to 12.81)	133.33 (96.06 to 184.89)
2038	7.12 (4.51 to 11.20)	110.29 (69.92 to 173.63)	9.48 (6.83 to 13.14)	134.68 (97.04 to 186.77)
2039	7.27 (4.61 to 11.45)	111.15 (70.47 to 174.99)	9.71 (7.00 to 13.47)	136.05 (98.03 to 188.67)
**2040**	**7.43 (4.71 to 11.69)**	**112.03 (71.03 to 176.36)**	**9.95 (7.17 to 13.80)**	**137.44 (99.04 to 190.59)**
2041	7.58 (4.81 to 11.94)	112.91 (71.59 to 177.74)	10.19 (7.34 to 14.12)	138.84 (100.05 to 192.52)
2042	7.74 (4.91 to 12.18)	113.80 (72.16 to 179.14)	10.42 (7.51 to 14.45)	140.25 (101.07 to 194.48)
2043	7.90 (5.01 to 12.43)	114.69 (72.73 to 180.54)	10.66 (7.68 to 14.77)	141.68 (102.10 to 196.45)
2044	8.05 (5.11 to 12.68)	115.59 (73.30 to 181.96)	10.89 (7.85 to 15.10)	143.12 (103.14 to 198.45)
2045	8.21 (5.21 to 12.93)	116.50 (73.88 to 183.38)	11.13 (8.02 to 15.43)	144.58 (104.19 to 200.46)
2046	8.37 (5.31 to 13.18)	117.42 (74.47 to 184.82)	11.36 (8.19 to 15.75)	146.05 (105.26 to 202.50)

2047	8.53 (5.41 to 13.43)	118.34 (75.05 to 186.27)	11.60 (8.36 to 16.08)	147.53 (106.33 to 204.56)
2048	8.69 (5.51 to 13.68)	119.27 (75.65 to 187.73)	11.84 (8.53 to 16.41)	149.04 (107.41 to 206.63)
2049	8.86 (5.62 to 13.94)	120.21 (76.24 to 189.20)	12.08 (8.70 to 16.74)	150.55 (108.51 to 208.73)
**2050**	**9.02 (5.72 to 14.20)**	**121.16 (76.85 to 190.69)**	**12.32 (8.88 to 17.08)**	**152.08 (109.62 to 210.85)**

Abbreviations: UI, uncertainty interval.

The relative risk for this disease in this study is smoking.

Table S17. Forecasted prevalent cases and rates (per 100,000 population) of age-related macular degeneration by sex, comparing estimates without risk factor, all ages, 1990–2050

	Male (95% UI)		Female (95% UI)	
**Year**	**Number, million**	**Rate (per 100,000)**	**Number, million**	**Rate (per 100,000)**
**2021**	**3.40 (2.81 to 4.17)**	**85.86 (58.68 to 125.43)**	**4.66 (3.88 to 5.65)**	**118.46 (91.15 to 153.87)**
2022	4.24 (2.89 to 6.19)	88.19 (60.27 to 128.82)	5.35 (4.12 to 6.96)	103.83 (79.86 to 134.90)
2023	4.35 (2.98 to 6.36)	88.88 (60.75 to 129.82)	5.52 (4.25 to 7.17)	104.88 (80.68 to 136.27)
2024	4.48 (3.06 to 6.54)	89.57 (61.23 to 130.83)	5.69 (4.38 to 7.40)	105.95 (81.50 to 137.65)
2025	4.60 (3.14 to 6.72)	90.27 (61.71 to 131.85)	5.87 (4.51 to 7.62)	107.02 (82.33 to 139.04)
2026	4.72 (3.23 to 6.90)	90.98 (62.19 to 132.88)	6.05 (4.65 to 7.86)	108.11 (83.16 to 140.45)
2027	4.85 (3.32 to 7.09)	91.69 (62.68 to 133.92)	6.23 (4.80 to 8.10)	109.20 (84.01 to 141.87)
2028	4.99 (3.41 to 7.29)	92.41 (63.17 to 134.96)	6.43 (4.95 to 8.35)	110.31 (84.86 to 143.30)
2029	5.13 (3.51 to 7.49)	93.13 (63.67 to 136.01)	6.63 (5.10 to 8.61)	111.43 (85.72 to 144.75)
**2030**	**5.27 (3.61 to 7.70)**	**93.86 (64.17 to 137.07)**	**6.84 (5.26 to 8.88)**	**112.56 (86.60 to 146.22)**
2031	5.42 (3.70 to 7.91)	94.59 (64.68 to 138.14)	7.05 (5.42 to 9.16)	113.70 (87.48 to 147.70)
2032	5.56 (3.80 to 8.12)	95.33 (65.18 to 139.22)	7.26 (5.59 to 9.43)	114.85 (88.36 to 149.19)
2033	5.71 (3.90 to 8.34)	96.08 (65.70 to 140.30)	7.48 (5.75 to 9.71)	116.02 (89.26 to 150.70)
2034	5.86 (4.01 to 8.56)	96.83 (66.21 to 141.40)	7.70 (5.93 to 10.00)	117.19 (90.17 to 152.22)
2035	6.01 (4.11 to 8.78)	97.59 (66.73 to 142.50)	7.93 (6.10 to 10.30)	118.38 (91.09 to 153.76)
2036	6.16 (4.21 to 8.99)	98.35 (67.26 to 143.61)	8.15 (6.27 to 10.59)	119.58 (92.01 to 155.32)
2037	6.30 (4.31 to 9.21)	99.12 (67.78 to 144.73)	8.37 (6.44 to 10.87)	120.79 (92.95 to 156.89)
2038	6.45 (4.41 to 9.41)	99.89 (68.32 to 145.86)	8.59 (6.61 to 11.15)	122.01 (93.89 to 158.47)
2039	6.59 (4.51 to 9.62)	100.67 (68.85 to 146.99)	8.80 (6.77 to 11.43)	123.25 (94.84 to 160.08)
**2040**	**6.73 (4.60 to 9.82)**	**101.46 (69.39 to 148.14)**	**9.01 (6.94 to 11.71)**	**124.50 (95.81 to 161.69)**
2041	6.87 (4.70 to 10.03)	102.25 (69.94 to 149.29)	9.23 (7.10 to 11.98)	125.76 (96.78 to 163.33)
2042	7.01 (4.79 to 10.23)	103.05 (70.49 to 150.45)	9.44 (7.26 to 12.26)	127.03 (97.76 to 164.98)
2043	7.15 (4.89 to 10.44)	103.86 (71.04 to 151.63)	9.65 (7.43 to 12.53)	128.32 (98.75 to 166.65)
2044	7.29 (4.99 to 10.65)	104.67 (71.60 to 152.81)	9.86 (7.59 to 12.81)	129.62 (99.75 to 168.33)
2045	7.44 (5.09 to 10.86)	105.49 (72.16 to 154.00)	10.08 (7.75 to 13.08)	130.93 (100.77 to 170.03)
2046	7.58 (5.19 to 11.07)	106.31 (72.73 to 155.20)	10.29 (7.92 to 13.36)	132.26 (101.79 to 171.75)

2047	7.72 (5.28 to 11.28)	107.14 (73.30 to 156.41)	10.50 (8.08 to 13.64)	133.59 (102.82 to 173.49)
2048	7.87 (5.38 to 11.49)	107.98 (73.87 to 157.62)	10.72 (8.25 to 13.92)	134.95 (103.87 to 175.24)
2049	8.02 (5.49 to 11.70)	108.82 (74.45 to 158.85)	10.93 (8.42 to 14.20)	136.31 (104.92 to 177.01)
**2050**	**8.17 (5.59 to 11.92)**	**109.67 (75.04 to 160.09)**	**11.15 (8.58 to 14.48)**	**137.69 (105.98 to 178.80)**

Abbreviations: UI, uncertainty interval.

The relative risk for this disease in this study is smoking.
