# Supplementary material for: Global burden of vision impairment due to age-related macular degeneration, 1990–2021, with forecasts to 2050: a systematic analysis for the Global Burden of Disease Study 2021
Source: Lancet Glob Health. 2025 Jun 25;13(7):e1175–90. doi: 10.1016/S2214-109X(25)00143-3 (PMC12208786; doi:10.1016/S2214-109X(25)00143-3)
Supplement: Supplementary appendix 3 [file mmc3.pdf]

THE LANCET
Global Health

Supplementary appendix 3

This appendix formed part of the original submission and has been peer reviewed. We post it as supplied by the authors.

Supplement to: GBD 2021 Global AMD Collaborators. Global burden of vision impairment due to age-related macular degeneration, 1990–2021, with forecasts to 2050: a systematic analysis for the Global Burden of Disease Study 2021. *Lancet Glob Health* 2025; **13:** e1175–90.

Appendix 3: Authorship appendix to "Global burden of vision impairment due to age-related macular degeneration, 1990–2021, with forecasts to 2050: a systematic analysis for the Global Burden of Disease Study 2021"

This appendix provides further authorship detail for "Global burden of vision impairment due to age-related macular degeneration, 1990–2021, with forecasts to 2050: a systematic analysis for the Global Burden of Disease Study 2021"

Table of Contents

GBD 2021 Global AMD Collaborators……………………………………………………………… 2

Affiliations…………………………………………………………………………………………………… 3

Authors' Contributions……………………………………………………………………………………. 13

- Providing data or critical feedback on data sources…………………………………… 13
- Developing methods or computational machinery……………………………………. 14
- Providing critical feedback on methods or results……………………………………… 14
- Drafting the work or revising it critically for important intellectual content…. 15
- Managing the estimation or publications process……………………………………… 16

GBD 2021 Global AMD Collaborators

Yi Deun Jeong*, Seoyoung Park*, Min Seo Kim*, Sung Hwi Hong, Hasan Aalruz, Yohannes Habtegiorgis Abate, Rouzbeh Abbasgholizadeh, Samar Abd ElHafeez, Auwal Abdullahi, Richard Gyan Aboagye, Lucas Guimarães Abreu, Ahmed Abu-Zaid, Isaac Yeboah Addo, Habeeb Omoponle Adewuyi, Saira Afzal, Williams Agyemang-Duah, Aqeel Ahmad, Danish Ahmad, Sajjad Ahmad, Ali Ahmadi, Hooman Ahmadzadeh, Ali Ahmed, Ayman Ahmed, Haroon Ahmed, Syed Anees Ahmed, Amjad S Al Mosa, Rasmieh Mustafa Al-amer, Mohammed Albashtawy, Ahmad Samir Alfaar, Abdelazeem M Algammal, Fadwa Naji Alhalaiqa, Abid Ali, Syed Shujait Ali, Waad Ali, Ahmad Alrawashdeh, Awais Altaf, Vera L Alves Carneiro, Yaser Mohammed Al-Worafi, Hany Aly, Sofia Androudi, Boluwatife Stephen Anuoluwa, Saeid Anvari, Anayochukwu Edward Anyasodor, Jalal Arabloo, Mosab Arafat, Aleksandr Y Aravkin, Demelash Areda, Anton A Artamonov, Akram M Asbeutah, Seyyed Shamsadin Athari, Maha Moh'd Wahbi Atout, Alok Atreya, Lemessa Assefa A Ayana, Shahkaar Aziz, Ahmed Y. Azzam, Saeed Bahramian, Ruhai Bai, Atif Amin Baig, Soham Bandyopadhyay, Zarrin Basharat, Mohammad-Mahdi Bastan, Maryam Bemanalizadeh, Akshaya Srikanth Bhagavathula, Nikha Bhardwaj, Pankaj Bhardwaj, Sonu Bhaskar, Ajay Nagesh Bhat, Jasvinder Singh Bhatti, Fassikaw Kebede Bizuneh, Paul Svitil Briant, Gabrielle Britton, Yasser Bustanji, Zahid A Butt, Florentino Luciano Caetano dos Santos, Mehtap Çakmak Barsbay, Fan Cao, Vijay Kumar Chattu, Anis Ahmad Chaudhary, Patrick R Ching, Hitesh Chopra, Sonali Gajanan Choudhari, Dinh-Toi Chu, João M Coelho, Natalia Cruz-Martins, Omid Dadras, Xiaochen Dai, Emanuele D'Amico, Samuel Demissie Darcho, Ana Maria Dascalu, Nihar Ranjan Dash, Mohsen Dashti, Anna Dastiridou, Nikolaos Dervenis, Aragaw Tesfaw Desale, Vinoth Gnana Chellaiyan Devanbu, Amol S Dhane, Daniel Diaz, Michael J Diaz, Thanh Chi Do, Ojas Prakashbhai Doshi, Ashel Chelsea Dsouza, Hisham Atan Edinur, Ferry Efendi, Michael Ekholuenetale, Rabie Adel El Arab, Ibrahim Farahat El Bayoumy, Muhammed Elhadi, Chadi Eltaha, Mohammad Hassan Emamian, Adeniyi Francis Fagbamigbe, Ayesha Fahim, Hossein Farrokhpour, Ali Fatehizadeh, Timur Fazylov, Alireza Feizkhah, Ginenus Fekadu, Nuno Ferreira, Florian Fischer, Ida Fitriana, Ali Forouhari, Matteo Foschi, João M M Furtado, Blima Fux, Sridevi G, Muktar A Gadanya, Abhay Motiramji Gaidhane, Aravind P Gandhi, Balasankar Ganesan, Ravindra Kumar Garg, Rupesh K Gautam, Miglas Welay Gebregergis, Mesfin Gebrehiwot, Delaram J Ghadimi, Sadegh Ghafarian, Mahaveer Golechha, Pouya Goleij, Ayman Grada, Shi-Yang Guan, Snigdha Gulati, Sapna Gupta, Veer Bala Gupta, Vivek Kumar Gupta, Roberth Steven Gutiérrez-Murillo, Awoke Derbie Habteyohannes, Arvin Haj-Mirzaian, Sobia Ahsan Halim, Ahmed I Hasaballah, Md. Kamrul Hasan, Hamidreza Hasani, Jiawei He, Golnaz Heidari, Mojtaba Heydari, Nguyen Quoc Hoan, Ramesh Holla, Mehdi Hosseinzadeh, Chengxi Hu, Junjie Huang, Salman Hussain, Segun Emmanuel Ibitoye, Nayu Ikeda, Irena M Ilic, Milena D Ilic, Mustapha Immurana, Arit Inok, Lalu Muhammad Irham, Md. Rabiul Islam, Sheikh Mohammed Shariful Islam, Chidozie Declan Iwu, Louis Jacob, Ammar Abdulrahman Jairoun, Mihajlo Jakovljevic, Talha Jawaid, Shubha Jayaram, Zixiang Ji, Jost B Jonas, Nitin Joseph, Charity Ehimwenma Joshua, Vidya Kadashetti, Ankita Kankaria, Kehinde Kazeem Kanmodi, Neeti Kapoor, Ibraheem M Karaye, Soujanya Kaup, Gbenga A Kayode, Yousef Saleh Khader, Himanshu Khajuria, Ajmal Khan, Atulya Aman Khosla, Yun Jin Kim, Adnan Kisa, Shivakumar KM, Kewal Krishan, Mohammed Kuddus, Mukhtar Kulimbet, Nithin Kumar, Satyajit Kundu, Chandrakant Lahariya, Dharmesh Kumar Lal, Iván Landires, Van Charles Lansingh, Ariane Laplante-Lévesque, Caterina Ledda, Munjae Lee, Seung Won Lee, Wei-Chen Lee, Stephen S Lim, Xuefeng Liu, José Francisco López-Gil, Zheng Feei Ma, Kashish Malhotra, Vahid Mansouri, Roy Rillera Marzo, Alireza Mashaghi, Yasith Mathangasinghe, Andrea Maugeri, Asim Mehmood, Tesfahun Mekene Meto,

Hadush Negash Meles, Endalkachew Belayneh Melese, Tomislav Mestrovic, Sachith Mettananda, Irmina Maria Michalek, Andreea Mirica, Abdalla Z Mohamed, Nouh Saad Mohamed, Abdollah Mohammadian-Hafshejani, Ali H Mokdad, Fateme Montazeri, Maryam Moradi, Rohith Motappa, Sumaira Mubarik, Kavita Munjal, Yanjinlkham Munkhsaikhan, Amin Nabavi, Ganesh R Naik, Vinay Nangia, Shumaila Nargus, Zuhair S Natto, Muhammad Naveed, Biswa Prakash Nayak, Athare Nazri-Panjaki, Van Thanh Nguyen, Robina Khan Niazi, Syed Toukir Ahmed Noor, Mamoona Noreen, Fred Nugen, Bogdan Oancea, Osamudiamen Cyril Obasuyi, Andrew T Olagunju, Sok King Ong, Michal Ordak, Verner N Orish, Mayowa O Owolabi, Jagadish Rao Padubidri, Georgios D Panos, Leonidas D Panos, Shahina Pardhan, Romil R Parikh, Sungchul Park, Tae Hwan Park, Maja Pasovic, Roberto Passera, Jay Patel, Shrikant Pawar, Prince Peprah, Arokiasamy Perianayagam, Mohsen Pourazizi, Jalandhar Pradhan, Jagadeesh Puvvula, Nameer Hashim Qasim, Venkatraman Radhakrishnan, Pankaja Raghav, Fakher Rahim, Vafa Rahimi-Movaghar, Mohammad Hifz Ur Rahman, Mosiur Rahman, Muhammad Aziz Rahman, Shayan Rahmani, Mohammad Rahmanian, Pushp Lata Rajpoot, Sowmya J Rao, Mohammad-Mahdi Rashidi, Salman Rawaf, Elrashdy M. Moustafa Mohamed Redwan, Mohsen Rezaeian, Sara Riaz, Moustaq Karim Khan Rony, Himanshu Sekhar Rout, Priyanka Roy, Aly M A Saad, Zahra Saadatian, Cameron John Sabet, Basema Ahmad Saddik, Umar Saeed, Sare Safi, Sher Zaman Safi, Fatemeh Saheb Sharif-Askari, Narjes Saheb Sharif-Askari, Amirhossein Sahebkar, Pragyan Monalisa Sahoo, S. Mohammad Sajadi, Mohamed A Saleh, Yoseph Leonardo Samodra, Abdallah M Samy, Tanmay Sarkar, Brijesh Sathian, Maheswar Satpathy, Jennifer Saulam, Monika Sawhney, Ganesh Kumar Saya, Siddharthan Selvaraj, Yashendra Sethi, Allen Seylani, Jaffer Shah, Amira A Shaheen, Samiah Shahid, Moyad Jamal Shahwan, Masood Ali Shaikh, Muhammad Aaqib Shamim, Javad Sharifi Rad, Anupam Sharma, Vishal Sharma, Maryam Shayan, Mahabalesh Shetty, Pavanchand H Shetty, Premalatha K Shetty, Mika Shigematsu, Aminu Shittu, Negussie Boti Sidamo, Emmanuel Edwar Siddig, Mithun Sikdar, Jasvinder A Singh, Paramdeep Singh, Puneetpal Singh, Surjit Singh, Raul A R C Sousa, Chandrashekhar T Sreeramareddy, Chandan Kumar Swain, Lukasz Szarpak, Seyyed Mohammad Tabatabaei, Ker-Kan Tan, Hugh R Taylor, Mohamad-Hani Temsah, Ramna Thakur, Jansje Henny Vera Ticoalu, Krishna Tiwari, Marcos Roberto Tovani-Palone, Ngoc Ha Tran, Thang Huu Tran, Munkhtuya Tumurkhuu, Saeed Ullah, Muhammad Umair, Sanaz Vahdati, Shaopan Wang, Muhammad Waqas, Nuwan Darshana Wickramasinghe, Kazumasa Yamagishi, Amir Yarahmadi, Pengpeng Ye, Arzu Yiğit, Yazachew Engida Engida Yismaw, Naohiro Yonemoto, Aurora Zanghì, Mohammed G M Zeariya, Zhi-Jiang Zhang, Claire Chenwen Zhong, Abzal Zhumagaliuly, Makan Ziafati, Magdalena Zielińska, Sa'ed H Zyoud, Jae Il Shin**, Dong Keon Yon**

*Lead authors
**Senior authors
Dong Keon Yon is the corresponding author.

Affiliations

Department of Medicine (Y Jeong MD), Center for Digital Health (S Park PhD), Kyung Hee University College of Medicine, Seoul, South Korea; Broad Institute of MIT and Harvard, Cambridge, MA, USA (M Kim MD); Department of Radiology (A Haj-Mirzaian MD), Massachusetts General Hospital, Boston, MA, USA (M Kim MD); Department of Pediatrics (S Hong MD), Yonsei University College of Medicine, Seoul, South Korea; Research Department (S Hong MD), Electronic Medical Records for the Developing World, York, UK; Department of Nursing (H Aalruz PhD), Al Zaytoonah University of Jordan, Amman, Jordan;

Department of Clinical Governance and Quality Improvement (Y H Abate MSc), Aleta Wondo General Hospital, Aleta Wondo, Ethiopia; Doheny Eye Institute (R Abbasgholizadeh MD), University of California Los Angeles, Los Angeles, CA, USA; Department of Epidemiology (S Abd ElHafeez DrPH), Alexandria University, Alexandria, Egypt; Department of Physiotherapy (A Abdullahi PhD), Department of Community Medicine (Prof M A Gadanya MD), Bayero University Kano, Kano, Nigeria; Department of Physiotherapy (A Abdullahi PhD), Federal University Wukari, Wukari, Nigeria; Department of Family and Community Health (R G Aboagye MPH), Institute of Health Research (M Immurana PhD), Department of Microbiology and Immunology (Prof V N Orish PhD), University of Health and Allied Sciences, Ho, Ghana; School of Population Health (R G Aboagye MPH, Prof B A Saddik PhD), Centre for Social Research in Health (I Y Addo PhD), The George Institute for Global Health (P Ye PhD), University of New South Wales, Sydney, NSW, Australia; Department of Pediatric Dentistry (Prof L Abreu PhD), Federal University of Minas Gerais, Belo Horizonte, Brazil; Department of Biochemistry and Molecular Medicine (A Abu-Zaid PhD), Alfaisal University, Riyadh, Saudi Arabia; College of Graduate Health Sciences (A Abu-Zaid PhD), Department of Ophthalmology (A Nabavi MD), University of Tennessee, Memphis, TN, USA; School of Medicine (I Y Addo PhD), University of Sydney, Sydney, NSW, Australia; Department of Educational Counselling and Developmental Psychology (H O Adewuyi PhD), Department of Epidemiology and Medical Statistics (A F Fagbamigbe PhD), Department of Health Promotion and Education (S Ibitoye PhD), Department of Medicine (Prof M O Owolabi DrM), University of Ibadan, Ibadan, Nigeria; Department of Educational Psychology (H O Adewuyi PhD), University of Johannesburg, Johannesburg, South Africa; Department of Community Medicine (Prof S Afzal PhD), King Edward Memorial Hospital, Lahore, Pakistan; Department of Public Health (Prof S Afzal PhD), Public Health Institute, Lahore, Pakistan; Department of Public Health Sciences (W Agyemang-Duah PhD), Queen's University, Kingston, ON, Canada; College of Medicine (A Ahmad PhD), Shaqra University, Shaqra, Saudi Arabia; School of Medicine and Psychology (D Ahmad PhD), Australian National University, Canberra, ACT, Australia; Public Health Foundation of India, Gandhinagar, India (D Ahmad PhD); Department of Health and Biological Sciences (S Ahmad PhD), Abasyn University, Peshawar, Pakistan; Department of Natural Sciences (S Ahmad PhD), Lebanese American University, Beirut, Lebanon; Department of Epidemiology and Biostatistics (A Ahmadi PhD), Modeling in Health Research Center (A Mohammadian-Hafshejani PhD), Shahrekord University of Medical Sciences, Shahrekord, Iran; Department of Epidemiology (A Ahmadi PhD), School of Medicine (D J Ghadimi MD, S Rahmani MD), Obesity Research Center (A Haj-Mirzaian MD), Student Research Committee (M Rahmanian MD), Social Determinants of Health Research Center (M Rashidi MD), Ophthalmic Research Center (S Safi PhD), Ophthalmic Research Center (ORC) (M Shayan MD), Shahid Beheshti University of Medical Sciences, Tehran, Iran; Bascom Palmer Eye Institute (H Ahmadzadeh MD), University of Miami, Miami, FL, USA; Department of Pharmacy Practice (A Ahmed PhD), Riphah Institute of Pharmaceutical Sciences, Islamabad, Pakistan; Division of Infectious Diseases and Global Public Health (IDGPH) (A Ahmed PhD), University of California San Diego, San Diego, CA, USA; Institute of Endemic Diseases (A Ahmed MSc), Unit of Basic Medical Sciences (E E Siddig MD), University of Khartoum, Khartoum, Sudan; Swiss Tropical and Public Health Institute (A Ahmed MSc), University of Basel, Basel, Switzerland; Department of Biosciences (H Ahmed PhD), COMSATS Institute of Information Technology, Islamabad, Pakistan; Brody School of Medicine (S Ahmed PhD), East Carolina University, Greenville, NC, USA; Department of Medicine (A S Al Mosa MD), Imam Abdulrahman Bin Faisal University, Dammam, Saudi Arabia; School of Nursing (R M Al-amer PhD), Yarmouk University, Irbid, Jordan; School of Nursing and Midwifery (R M Al-amer PhD), Western Sydney University, Sydney, NSW, Australia; Department of Community and Mental Health (Prof M Albashtawy PhD), Al al-Bayt University, Mafraq, Jordan; Department of Ophthalmology (A Alfaar PhD), Lasermed MVZ GmbH, Berlin, Germany; St Paul Eye Unit (A Alfaar PhD), Royal Liverpool University Hospital, Liverpool, United Kingdom; Department of Bacteriology, Immunology, and Mycology (Prof A M Algammal PhD), Suez Canal University, Ismailia, Egypt; College of Nursing (Prof F N Alhalaiqa PhD), Social and Economic Survey

Research Institute (Prof A Perianayagam PhD), Qatar University, Doha, Qatar; Department of Zoology (A Ali PhD), Abdul Wali Khan University Mardan, Mardan, Pakistan; Center for Biotechnology and Microbiology (S S Ali PhD), University of Swat, Swat, Pakistan; Department of Geography (W Ali PhD), Sultan Qaboos University, Muscat, Oman; Department of Allied Medical Sciences (A Alrawashdeh PhD), Department of Public Health (Prof Y S Khader PhD), Jordan University of Science and Technology, Irbid, Jordan; Institute of Molecular Biology and Biotechnology (A Altaf PhD, S Shahid PhD), University Institute of Public Health (S Nargus PhD), Research Centre for Health Sciences (RCHS) (S Shahid PhD), The University of Lahore, Lahore, Pakistan; School of Sciences (V Alves Carneiro PhD), University of Minho, Braga, Portugal; Department of Medical Sciences (Prof Y M Al-Worafi PhD), Azal University for Human Development, Sana'a, Yemen; Department of Clinical Sciences (Prof Y M Al-Worafi PhD), University of Science and Technology of Fujairah, Fujairah, United Arab Emirates; Department of Pediatrics (Prof H Aly MD), Department of Thoracic Surgery (S Gulati MD), Lerner Research Institute (X Liu PhD), Cleveland Clinic, Cleveland, OH, USA; Department of Medicine (S Androudi PhD), University of Thessaly, Volos, Greece; Department of Environmental and Occupational Health (B S Anuoluwa MPH), University of Medical Sciences, Ondo, Ondo, Nigeria; Regenerative Medicine, Organ Procurement and Transplantation Multi-disciplinary Center (S Anvari MD), Department of Social Medicine and Epidemiology (A Feizkhah MD), Guilan University of Medical Sciences, Rasht, Iran; Rural Health Research Institute (A E Anyasodor PhD), Charles Sturt University, Orange, NSW, Australia; Health Management and Economics Research Center (J Arabloo PhD), School of Medicine (M Bastan MD), Department of Ophthalmology (H Hasani MD), Iran University of Medical Sciences, Tehran, Iran (M Moradi MD); College of Pharmacy (M Arafat PhD), Al Ain University, Abu Dhabi, United Arab Emirates; Department of Applied Mathematics (A Y Aravkin PhD), Institute for Health Metrics and Evaluation (A Y Aravkin PhD, P S Briant BS, X Dai PhD, J He MSc, Prof S S Lim PhD, T Mestrovic PhD, Prof A H Mokdad PhD, M Pasovic M.Ed.), Department of Health Metrics Sciences, School of Medicine (X Dai PhD, Prof S S Lim PhD, Prof A H Mokdad PhD), School of Health Systems and Public Health (C Iwu MPH), University of Washington, Seattle, WA, USA; Department of Health Metrics Sciences, School of Medicine (A Y Aravkin PhD), University of Washington, Seattle, WA, United States of America; College of Art and Science (D Areda PhD), Ottawa University, Surprise, AZ, USA; School of Life Sciences (D Areda PhD), Arizona State University, Tempe, AZ, USA; Institute for Biomedical Problems (A A Artamonov PhD), Russian Academy of Sciences, Moscow, Russia; Department of Radiologic Sciences (Prof A M Asbeutah PhD), Kuwait University, Sulaibikhat, Kuwait; Department of Medical Radiation Sciences (Prof A M Asbeutah PhD), Monash University, Melbourne, VIC, Australia; Department of Immunology (S Athari PhD), Zanjan University of Medical Sciences, Zanjan, Iran; Faculty of Nursing (M M W Atout PhD), Philadelphia University, Amman, Jordan; Department of Forensic Medicine (A Atreya MD), Lumbini Medical College, Palpa, Nepal; Department of Public Health (L A A Ayana MPH), Department of Pharmacy (G Fekadu PhD), Wollega University, Nekemte, Ethiopia; Department of Health Behavior and Society (L A A Ayana MPH), Jimma University, Jimma, Ethiopia; Institute of Biotechnology and Genetic Engineering (S Aziz MS), The University of Agriculture, Peshawar, Pakistan; ASIDE Healthcare, Lewes, DE, USA (A Azzam MD); Faculty of Medicine (A Azzam MD), October 6 University, 6th of October City, Egypt; School of Medicine (S Bahramian MD), Department of Pediatrics (M Bemanalizadeh MD), Department of Ophthalmology (A Forouhari MD), Ophthalmology Department (M Pourazizi MD), Isfahan University of Medical Sciences, Isfahan, Iran; Clinical Research Center (R Bai MD), Children's Hospital of Nanjing Medical University, Nanjing, China; International Medical School (A A Baig PhD), Management and Science University, Alam, Malaysia; Nuffield Department of Surgical Sciences (S Bandyopadhyay MPH), University of Oxford, Oxford, UK; Department of Neurosurgery (S Bandyopadhyay MPH), University of Southampton, Southampton, United Kingdom; Alpha Genomics Private Limited, Islamabad, Pakistan (Z Basharat PhD); Non-communicable Diseases Research Center (M Bastan MD, S Rahmani MD, M Rashidi MD), Department of Pediatric Neurology (M Bemanalizadeh MD), Iranian Research Center for HIV/AIDS (IRCHA) (O Dadras PhD), School of Medicine (H Farrokhpour MD),

Department of Ophthalmology (S Ghafarian MD), Digestive Diseases Research Institute (V Mansouri MD), Sina Trauma and Surgery Research Center (Prof V Rahimi-Movaghar MD), Tehran University of Medical Sciences, Tehran, Iran; Department of Public Health (A S Bhagavathula PhD), North Dakota State University, Fargo, ND, USA; Division of Gastroenterology and Hepatology (A S Bhagavathula PhD), Mayo Clinic, Jacksonville, FL, USA; Department of Anatomy (N Bhardwaj MD), Department of Community Medicine and Family Medicine (Prof P Bhardwaj MD, Prof P Raghav MD), School of Public Health (Prof P Bhardwaj MD), Department of Pharmacology (M Shamim MBBS, S Singh MD, K Tiwari MBBS), All India Institute of Medical Sciences, Jodhpur, India; Global Health Neurology Lab (S Bhaskar MD), NSW Brain Clot Bank, Sydney, NSW, Australia; Division of Cerebrovascular Medicine and Neurology (S Bhaskar MD), National Cerebral and Cardiovascular Center, Suita, Japan; Department of General Medicine (A N Bhat MD), Department of Community Medicine (N Joseph MD, N Kumar MD, R Motappa MD), Department of Forensic Medicine and Toxicology (Prof J Padubidri MD, P H Shetty MD), Manipal College of Dental Sciences, Mangalore (Prof P K Shetty MDS), Manipal Academy of Higher Education, Mangalore, India; Department of Human Genetics and Molecular Medicine (Prof J Bhatti PhD), Central University of Punjab, Bathinda, India; Department of Public Health (F Bizuneh MPH), Woldia University, Woldia, Ethiopia; Center for Neuroscience (G Britton PhD), Institute for Scientific Research and High Technology Services, Panama City, Panama; Gorgas Memorial Institute for Health Studies, Panama City, Panama (G Britton PhD); School of Pharmacy (Prof Y Bustanji PhD), The University of Jordan, Amman, Jordan; Department of Basic Biomedical Sciences (Prof Y Bustanji PhD), Clinical Sciences Department (N R Dash MD, N Saheb Sharif-Askari PhD), College of Medicine (Prof B A Saddik PhD, Prof M A Saleh PhD), Sharjah Institute of Medical Sciences (F Saheb Sharif-Askari PhD), University of Sharjah, Sharjah, United Arab Emirates; School of Public Health Sciences (Z A Butt PhD), University of Waterloo, Waterloo, ON, Canada; Al Shifa School of Public Health (Z A Butt PhD), Al Shifa Trust Eye Hospital, Rawalpindi, Pakistan; Harvard Business School (F Caetano dos Santos PhD), Department of Health Policy and Oral Epidemiology (Z S Natto DrPH), Department of Ophthalmology (M Shayan MD), Harvard University, Boston, MA, USA; Faculty of Health Sciences Healthcare Management Department (M Çakmak Barsbay PhD), Ankara University, Ankara, Turkiye; Department of Ophthalmology (F Cao MD), Beijing Institute of Ophthalmology, Beijing, China; Temerty Faculty of Medicine (V Chattu MD), University of Toronto, Toronto, ON, Canada; Department of Community Medicine (V Chattu MD), Datta Meghe Institute of Medical Sciences, Sawangi, India; Department of Biology (A A Chaudhary PhD), Al-Imam Mohammad Ibn Saud Islamic University, Riyadh, Saudi Arabia; Division of Infectious Diseases (P R Ching MD), Virginia Commonwealth University, Richmond, VA, USA; Centre for Research Impact & Outcome (H Chopra PhD), Chitkara University, Rajpura, India; Department of Community Medicine (Prof S G Choudhari MD), Jawaharlal Nehru Medical College, Wardha, India; The Interdisciplinary Research Group on Biomedicine and Health (D Chu PhD), Faculty of Applied Sciences (D Chu PhD), VNU International School (VNUIS), Hanoi, Viet Nam; University Hospital Center of Porto (J M Coelho MD), Institute for Research and Innovation in Health (i3S) (Prof N Cruz-Martins PhD), University of Porto, Porto, Portugal; Life and Health Sciences Research Institute (ICVS), School of Medicine, University of Minho, Braga, Portugal, Braga, Portugal (Prof N Cruz-Martins PhD); Research Center for Child Psychiatry (O Dadras PhD), University of Turku, Turku, Finland; Department of Medical and Surgical Sciences and Advanced Technologies "GF Ingrassia" (Prof E D'Amico MD, A Maugeri PhD), Department of Clinical and Experimental Medicine (Prof C Ledda PhD), University of Catania, Catania, Italy; Department of Public Health (S D Darcho MPH), Haramaya University, Harar, Ethiopia; Ophthalmology Department (A Dascalu PhD), Carol Davila University of Medicine and Pharmacy, Bucharest, Romania; Ophthalmology Department (A Dascalu PhD), Emergency University Hospital Bucharest, Bucuresti, Romania; Immunology Research Center (M Dashti MD), Tabriz University of Medical Sciences, Tabriz, Iran; 2nd University Ophthalmology Department (A Dastiridou MD), Department of Ophthalmology (N Dervenis MD), School of Medicine - First Department of Ophthalmology (Prof G D Panos MD(Res)), Aristotle University of Thessaloniki,

Thessaloniki, Greece; Ophthalmology Department (A Dastiridou MD), University of Thessaly, Greece; St Paul's Eye Unit (N Dervenis MD), Royal Liverpool University Hospital, Liverpool, UK; Department of Public Health (A T Desale MPH), Debre Tabor University, Debre Tabor, Ethiopia; Chettinad Hospital & Research Institute (Prof V Devanbu MD), Chettinad Academy of Research and Education, Chennai, India; Research and Development Cell (A S Dhane MBA), Dr. D. Y. Patil Vidyapeeth, Pune (Deemed to be University), Pune, India; Faculty of Science (Prof D Diaz PhD), National Autonomous University of Mexico, Mexico City, Mexico; College of Medicine (M J Diaz BS), University of Florida, Gainesville, FL, USA; Department of Medicine (T C Do MD), Pham Ngoc Thach University of Medicine, Ho Chi Minh City, Viet Nam; Independent Consultant, South Plainfield, NJ, USA (O P Doshi MS); Department of Medicine (A C Dsouza MBBS), Bangalore Medical College and Research Institute, Bangalore, India; School of Health Sciences (H A Edinur PhD), Universiti Sains Malaysia (University of Science Malaysia), Kubang Kerian, Malaysia; Advanced Nursing Department (F Efendi PhD), Universitas Airlangga (Airlangga University), Surabaya, Indonesia; Faculty of Science and Health (M Ekholuenetale PhD), University of Portsmouth, Hampshire, UK; Almoosa College of Health Sciences, Al Ahsa, Saudi Arabia (R A El Arab PhD); Department of Public Health and Community Medicine (Prof I F El Bayoumy DrPH), Tanta University, Tanta city, Egypt; School of Public Health (Prof I F El Bayoumy DrPH), Texila American University, Guyana, Guyana; Faculty of Medicine (M Elhadi MD), University of Tripoli, Tripoli, Libya; Houston Methodist Hospital, Houston, TX, USA (M Elhadi MD); Department of Pediatrics (C Eltaha MD), University of Texas, Dallas, TX, USA; Ophthalmic Epidemiology Research Center (Prof M Emamian PhD), Shahroud University of Medical Sciences, Shahroud, Iran; Research Centre for Healthcare and Community (A F Fagbamigbe PhD), Coventry University, Coventry, United Kingdom; Department of Oral Biology (A Fahim PhD), Riphah International University, Islamabad, Pakistan; Endocrinology and Metabolism Research Institute (H Farrokhpour MD), Non-Communicable Diseases Research Center (NCDRC), Tehran, Iran (F Montazeri MD); School of Engineering (A Fatehizadeh PhD), Edith Cowan University, Joondalup, WA, Australia; Laboratory of Experimental Medicine (T Fazylov MD), Research and Publication Activity Division (M Kulimbet MSc), Atchabarov Scientific-Research Institute of Fundamental and Applied Medicine (A Zhumagaliuly MD), Kazakh National Medical University, Almaty, Kazakhstan; Department of Infectious Diseases and Public Health (G Fekadu PhD), City University of Hong Kong, Hong Kong, China; Department of Social Sciences (Prof N Ferreira PhD), University of Nicosia, Nicosia, Cyprus; Institute of Public Health (F Fischer PhD), Charité Universitätsmedizin Berlin (Charité Medical University Berlin), Berlin, Germany; Department of Pharmacology (I Fitriana PhD), Gadjah Mada University, Yogyakarta, Indonesia; Department of Neuroscience (M Foschi MD), Multiple Sclerosis Research Center, Ravenna, Italy; Department of Biotechnological and Applied Clinical Sciences (M Foschi MD), University of L’Aquila, L’Aquila, Italy; Division of Ophthalmology (J M M Furtado MD), University of São Paulo, Ribeirão Preto, Brazil; Department of Pathology (Prof B Fux PhD), Federal University of Espirito Santo, Vitória, Brazil; Department of Community Medicine and Family Medicine (S G MD), All India Institute of Medical Sciences, Gorakhpur, India; Department of Community Medicine (Prof M A Gadanya MD), Aminu Kano Teaching Hospital, Kano, Nigeria; Department of Community Medicine (Prof A M Gaidhane MD), Datta Meghe Institute of Medical Sciences, Wardha, India; Department of Community Medicine and Family Medicine (A P Gandhi MD), All India Institute of Medical Sciences, Nagpur, India; School of Public Health (B Ganesan PhD), Institute of Health & Management, Australia, Melbourne, VIC, Australia; Department of Neurology (Prof R Garg MD), Era's Lucknow Medical College and Hospital, Era University, Lucknow, India, Lucknow, India; Department of Pharmacology (Prof R K Gautam PhD), Indore Institute of Pharmacy, Indore, India; Department of Midwifery (M W Gebregergis MSc), Department of Medical Laboratory Sciences (H N Meles MSc), Adigrat University, Adigrat, Ethiopia; Department of Environmental Health (M Gebrehiwot DSc), Wollo University, Dessie, Ethiopia; Department of Health Systems and Policy Research (Prof M Golechha PhD), Indian Institute of Public Health, Gandhinagar, India; Department of Genetics (P Goleij MSc), Sana Institute of Higher

Education, Sari, Iran; Universal Scientific Education and Research Network (USERN) (P Goleij MSc), Kermanshah University of Medical Sciences, Kermanshah, Iran; Department of Dermatology (A Grada MD), Department of Quantitative Health Science (X Liu PhD), Case Western Reserve University, Cleveland, OH, USA; Department of Epidemiology and Biostatistics (S Guan MD), Anhui Medical University, Hefei, China; Department of Toxicology (S Gupta MSc), Shriram Institute for Industrial Research, Delhi, India; School of Medicine (V Gupta PhD), Deakin University, Geelong, VIC, Australia; Faculty of Medicine Health and Human Sciences (Prof V K Gupta PhD), Australian Institute of Health Innovation (P Peprah MSc), Macquarie University, Sydney, NSW, Australia; Department of Biomedical Gerontology (R S Gutiérrez-Murillo PhD), Pontifical Catholic University of Rio Grande do Sul, Porto Alegre, Brazil; Department of Medical Microbiology (A D Habteyohannes PhD), Department of Pharmacology (Y E E Yismaw MSc), Bahir Dar University, Bahir Dar, Ethiopia; Natural and Medical Sciences Research Center (S A Halim PhD, A Khan PhD), University of Nizwa, Nizwa, Oman; Department of Zoology and Entomology (A I Hasaballah PhD, M G M Zeariya PhD), Al-Azhar University, Cairo, Egypt; Department of Health Research Methods, Evidence, and Impact (M Hasan MPH), Department of Psychiatry and Behavioural Neurosciences (Prof A T Olagunju PhD), McMaster University, Hamilton, ON, Canada; Department of Biochemistry and Molecular Biology (M Hasan MPH), Tejgaon College, Dhaka, Bangladesh; Independent Consultant, Santa Clara, CA, USA (G Heidari MD); Poostchi Ophthalmology Research Center (M Heydari PhD), Shiraz University of Medical Sciences, Shiraz, Iran; School of Dentistry (N Hoan DDS), Hanoi Medical University, Hanoi, Viet Nam; Kasturba Medical College, Mangalore (R Holla MD), Manipal Academy of Higher Education, Manipal, India; School of Computer Science (Prof M Hosseinzadeh PhD), Duy Tan University, Da Nang, Viet Nam; Jadara Research Center (Prof M Hosseinzadeh PhD), Jadara University, Irbid, Jordan; Department of Psychology (C Hu PhD), Tsinghua University, Beijing, China; Faculty of Medicine (J Huang MD), Jockey Club School of Public Health and Primary Care (C Zhong PhD), The Chinese University of Hong Kong, Hong Kong, China; Czech National Centre for Evidence-Based Healthcare and Knowledge Translation (S Hussain PhD), Institute of Biostatistics and Analyses (S Hussain PhD), Masaryk University, Brno, Czech Republic; Center for Nutritional Epidemiology and Policy Research (N Ikeda PhD), National Institutes of Biomedical Innovation, Health and Nutrition, Settsu, Osaka, Japan; Faculty of Medicine (I M Ilic PhD), University of Belgrade, Belgrade, Serbia; Faculty of Medical Sciences (Prof M D Ilic PhD), University of Kragujevac, Kragujevac, Serbia; Faculty of Health and Life Sciences (A Inok PhD), University of Exeter, Exeter, UK; Faculty of Pharmacy (L M Irham PhD), Universitas Ahmad Dahlan, Yogyakarta, Indonesia; School of Pharmacy (M Islam PhD), BRAC University, Dhaka, Bangladesh; Institute for Physical Activity and Nutrition (Prof S Islam PhD), Deakin University, Burwood, VIC, Australia; Department of Physical and Medicine (L Jacob MD), Université Paris Cité, Paris, France; Research and Development Unit (L Jacob MD), Biomedical Research Networking Center for Mental Health Network (CiberSAM), Barcelona, Spain; Department of Health and Safety (A A Jairoun PhD), Dubai Municipality, Dubai, United Arab Emirates; The World Academy of Sciences UNESCO, Trieste, Italy (Prof M Jakovljevic PhD); Shaanxi University of Technology, Hanzhong, China (Prof M Jakovljevic PhD); Department of Pharmacology (T Jawaid PhD), Imam Mohammad Ibn Saud Islamic University, Riyadh, Saudi Arabia; Department of Biochemistry (Prof S Jayaram MD), Government Medical College, Mysuru, India; Department of Public Health (Z Ji MMed), Tongji University, Shanghai, China; Rothschild Foundation Hospital (Prof J B Jonas MD), Institut Français de Myopie, Paris, France; Singapore Eye Research Institute (Prof J B Jonas MD), Singapore Eye Research Institute, Singapore, Singapore; Department of Economics (C E Joshua BSc), National Open University, Benin City, Nigeria; Department of Oral and Maxillofacial Pathology (V Kadashetti MDS), Department of Public Health Dentistry (Prof S KM MD), Krishna Vishwa Vidyapeeth (Deemed to be University), Karad, India; Department of Community Medicine and Family Medicine (A Kankaria MD), Department of Radiodiagnosis (P Singh MD), All India Institute of Medical Sciences, Bathinda, India; Faculty of Dentistry (K K Kanmodi MPH), University of Puthisastra, Phnom Penh, Cambodia; Office of the Executive Director

(K K Kanmodi MPH), Cephas Health Research Initiative Inc, Ibadan, Nigeria; Department of Forensic Science (N Kapoor PhD), Government Institute of Forensic Science Nagpur, Nagpur, India; School of Health Professions and Human Services (I M Karaye MD), Hofstra University, Hempstead, NY, USA; Department of Anesthesiology (I M Karaye MD), Montefiore Medical Center, Bronx, NY, USA; Department of Ophthalmology (S Kaup MS), Yenepoya University, Mangalore, India; International Research Center of Excellence (G A Kayode PhD), Institute of Human Virology Nigeria, Abuja, Nigeria; Julius Centre for Health Sciences and Primary Care (G A Kayode PhD), Utrecht University, Utrecht, Netherlands; Amity Institute of Forensic Sciences (H Khajuria PhD, B P Nayak PhD), Amity Institute of Pharmacy (K Munjal PhD), Amity University, Noida, India; Department of Internal Medicine (A A Khosla MD), Corewell Health East William Beaumont University Hospital, Royal Oak, MI, USA; Department of Medical Oncology (A A Khosla MD), Miami Cancer Institute, Miami, FL, USA; School of Traditional Chinese Medicine (Y Kim PhD), Xiamen University Malaysia, Sepang, Malaysia; School of Health Sciences (Prof A Kisa PhD), Kristiania University College, Oslo, Norway; Department of International Health and Sustainable Development (Prof A Kisa PhD), Tulane University, New Orleans, LA, USA; Department of Anthropology (Prof K Krishan PhD), Institute of Forensic Science & Criminology (V Sharma PhD), Panjab University, Chandigarh, India; Department of Biochemistry (Prof M Kuddus PhD), Department of Public Health (M G M Zeariya PhD), University of Hail, Hail, Saudi Arabia; Center of Medicine and Public Health (M Kulimbet MSc), Director of Central Asia Research Collaboration Group (Prof F Rahim PhD), Asfendiyarov Kazakh National Medical University, Almaty, Kazakhstan; Department of Public Health (S Kundu MPH), Griffith University, Gold Coast, QLD, Australia; Integrated Department of Epidemiology, Health Policy, Preventive Medicine and Pediatrics (Prof C Lahariya MD), Foundation for People-centric Health Systems, New Delhi, India; Centre for Health: The Specialty Practice, New Delhi, India (Prof C Lahariya MD); Indian Council of Medical Research, New Delhi, India (D K Lal MD); Unidad de Genética y Salud Pública (Prof I Landires MD), Instituto de Ciencias Médicas, Las Tablas, Panama; Ministry of Health (Prof I Landires MD), Hospital Joaquín Pablo Franco Sayas, Las Tablas, Panama; Chief Medical Office (Prof V C Lansingh PhD), HelpMeSee, New York, NY, USA; Mexican Institute of Ophthalmology, Queretaro, Mexico (Prof V C Lansingh PhD); Department of Behavioural Sciences and Learning (Prof A Laplante-Lévesque PhD), Linköping University, Linköping, Sweden; Department of Medical Science (M Lee PhD), Ajou University School of Medicine, Suwon, South Korea; Department of Precision Medicine (Prof S Lee MD), Sungkyunkwan University, Suwon-si, South Korea; Department of Family Medicine (W Lee PhD), University of Texas Medical Branch, Galveston, TX, USA; One Health Research Group (J López-Gil PhD), Universidad de Las Américas (University of the Americas), Quito, Ecuador; Centre for Public Health and Wellbeing (Z Ma PhD), University of the West of England, Bristol, UK; Rama Medical College Hospital and Research Centre, Uttar Pradesh, India (K Malhotra MBBS); Institute of Applied Health Research (K Malhotra MBBS), University of Birmingham, Birmingham, United Kingdom; Faculty of Humanities and Health Sciences (Prof R R Marzo MD), Curtin University, Sarawak, Malaysia; Jeffrey Cheah School of Medicine and Health Sciences (Prof R R Marzo MD), Monash University, Subang Jaya, Malaysia; Leiden Academic Centre for Drug Research (Prof A Mashaghi PhD), Leiden University, Leiden, Netherlands; Department of Anatomy and Developmental Biology (Y Mathangasinghe PhD), Monash University, Clayton, VIC, Australia; Department of Anatomy, Genetics and Biomedical Informatics (Y Mathangasinghe PhD), University of Colombo, Colombo, Sri Lanka; Department of Public Health (A Mehmood PhD, P Rajpoot PhD), Jazan University, Jazan, Saudi Arabia; Department of Public Health (T Mekene Meto MPH), School of Public Health (N Sidamo PhD), Arba Minch University, Arba Minch, Ethiopia; Department of Internal Medicine (E Melese MD), University of Gondar, Gondar, Ethiopia; Johns Hopkins University, Baltimore, MD, USA (E Melese MD); University Centre Varazdin (T Mestrovic PhD), University North, Varazdin, Croatia; Department of Paediatrics (Prof S Mettananda DPhil), University of Kelaniya, Ragama, Sri Lanka; University Paediatrics Unit (Prof S Mettananda DPhil), Colombo North Teaching Hospital, Ragama, Sri Lanka; National Cancer Registry (I Michalek PhD), Department of

Pathology (I Michalek PhD), Maria Sklodowska-Curie National Research Institute of Oncology, Warsaw, Poland; Department of Statistics and Econometrics (A Mirica PhD), Bucharest University of Economic Studies, Bucharest, Romania; Center for Brain and Health (A Z Mohamed PhD), New York University Abu Dhabi, Abu Dhabi, United Arab Emirates; Molecular Biology Unit (N S Mohamed MSc), Bio-Statistical and Molecular Biology Department (N S Mohamed MSc), Sirius Training and Research Centre, Khartoum, Sudan; Department of Ophthalmology & Vision Science (F Montazeri MD), University of California Davis, Sacramento, CA, USA; Unit of Pharmacotherapy, Epidemiology and Economics (Prof S Mubarik PhD), University of Groningen (Rijksuniversiteit Groningen), Groningen, Netherlands; Department of Epidemiology and Biostatistics (Prof S Mubarik PhD), School of Public Health (Prof Z Zhang PhD), Wuhan University, Wuhan, China; Department of Community and Global Health (Y Munkhsaikhan MD), The University of Tokyo, Tokyo, Japan; College of Medicine and Public Health (G R Naik PhD), Flinders University, Adelaide, SA, Australia; Department of Computer Science and IT (G R Naik PhD), Torrens University, Adelaide, SA, Australia; Suraj Eye Institute, Nagpur, India (V Nangia MD); Department of Dental Public Health (Z S Natto DrPH), King Abdulaziz University, Jeddah, Saudi Arabia; Department of Biotechnology (M Naveed PhD), University of Central Punjab, Lahore, Pakistan; Department of Health Promotion (A Nazri-Panjaki MSc), Zahedan University of Medical Sciences, Zahedan, Iran; Tuberculosis Group (V T Nguyen MD), Oxford University Clinical Research Unit, Vietnam, Ho Chi Minh City, Viet Nam; Department of General Medicine (V T Nguyen MD), Department of Internal Medicine (T H Tran MD), University of Medicine and Pharmacy at Ho Chi Minh City, Ho Chi Minh City, Viet Nam; International Islamic University Islamabad, Islamabad, Pakistan (R K Niazi PhD); Maternal and Child Health Division (S Noor MS), International Centre for Diarrhoeal Disease Research, Bangladesh, Dhaka, Bangladesh; Department of Statistics (S Noor MS), Shahjalal University of Science and Technology, Sylhet, Bangladesh; Department of Microbiology and Molecular Genetics (M Noreen PhD), The Women University Multan, Multan, Pakistan; Department of Radiology (F Nugen PhD), Department of Informatics and Radiology (S Vahdati MD), Mayo Clinic, Rochester, MN, USA; School of Information (F Nugen PhD), University of California Berkeley, Berkeley, CA, USA; Department of Applied Economics and Quantitative Analysis (Prof B Oancea PhD), University of Bucharest, Bucharest, Romania; Bioinformatics Department (Prof B Oancea PhD), National Institute of Research and Development for Biological Sciences, Bucharest, Romania; Department of Ophthalmology (O C Obasuyi MD), Irrua Specialist Teaching Hospital, Irrua, Nigeria; Department of Psychiatry (Prof A T Olagunju PhD), University of Lagos, Lagos, Nigeria; Department of Public Health (S Ong FAMS), Ministry of Health, Bandar Seri Begawan, Brunei; Institute of Health Sciences (S Ong FAMS), Universiti Brunei Darussalam, Bandar Seri Begawan, Brunei; Department of Pharmacotherapy and Pharmaceutical Care (M Ordak PhD), Department of Biochemistry and Pharmacogenomics (M Zielińska MPharm), Medical University of Warsaw, Warsaw, Poland; Sickle Cell Unit (Prof V N Orish PhD), Ho Teaching Hospital, Ho, Ghana; Department of Medicine (Prof M O Owolabi DrM), University College Hospital, Ibadan, Ibadan, Nigeria; School of Medicine - Division of Ophthalmology & Visual Sciences (Prof G D Panos MD(Res)), University of Nottingham, Nottingham, UK; Department of Neurology (L D Panos MD), University of Bern, Bern, Switzerland; Department of Neurology (L D Panos MD), University of Cyprus, Nicosia, Cyprus; Vision and Eye Research Institute (Prof S Pardhan PhD), Anglia Ruskin University, Cambridge, UK; Division of Health Policy and Management (R R Parikh MD), University of Minnesota, Minneapolis, MN, USA; Department of Health Policy and Management (S Park PhD), Department of Medicine (J Sharifi Rad PhD), Korea University, Seoul, South Korea; Department of Plastic and Reconstructive Surgery (T Park PhD), Dongtan Sacred Heart Hospital, Hwaseong, South Korea; Department of Medical Sciences (R Passera PhD), University of Torino, Torino, Italy; Department of Imaging (R Passera PhD), AOU Città della Salute e della Scienza di Torino, Torino, Italy; Global Health Governance Programme (J Patel BSc), University of Edinburgh, Edinburgh, UK; School of Dentistry (J Patel BSc), University of Leeds, Leeds, United Kingdom; Department of Genetics (S Pawar PhD), Yale University, New Haven, CT, USA; Department of Humanities

and Social Sciences (Prof J Pradhan PhD), National Institute of Technology Rourkela, Rourkela, India; Department of Biostatistics, Epidemiology, and Informatics (J Puvvula PhD), Department of Medical Ethics and Health Policy (S Riaz MSc), University of Pennsylvania, Philadelphia, PA, USA; Cihan University-Sulaimaniya Research Center (N H Qasim DSc), Cihan University-Sulaimaniya, Sulaymaniyah, Iraq; Department of Medical Oncology (Prof V Radhakrishnan MD), Cancer Institute (W.I.A), Chennai, India; Osh State University, Osh, Kyrgyzstan (Prof F Rahim PhD); College of Medicine and Health Sciences (M Rahman PhD), National University of Science and Technology, Sohar, Oman; Department of Population Science and Human Resource Development (Prof M Rahman DrPH), University of Rajshahi, Rajshahi, Bangladesh; Institute of Health and Wellbeing (Prof M Rahman PhD), Federation University Australia, Berwick, VIC, Australia; School of Nursing and Midwifery (Prof M Rahman PhD), La Trobe University, Melbourne, VIC, Australia; Department of Oral Pathology, Microbiology and Forensic Odontology (S Rao MDS), Sharavathi Dental College and Hospital, Shimogga, India; Department of Primary Care and Public Health (Prof S Rawaf MD), Imperial College London, London, UK; Academic Public Health England (Prof S Rawaf MD), Public Health England, London, United Kingdom; Department of Biological Sciences (Prof E M M Redwan PhD), King Abdulaziz University, Jeddah, Egypt; Department of Protein Research (Prof E M M Redwan PhD), Research and Academic Institution, Alexandria, Egypt; Department of Epidemiology and Biostatistics (Prof M Rezaeian PhD), Rafsanjan University of Medical Sciences, Rafsanjan, Iran; Department of Family Medicine (S Riaz MSc), McGill University, Montreal, QC, Canada; Miyan Research Institute (M Rony MPH), Miyan Research Institute, International University of Business Agriculture and Technology, Dhaka, Bangladesh; Department of Analytical and Applied Economics (Prof H Rout PhD, C Swain MPhil), RUSA Centre of Excellence in Public Policy and Governance (Prof H Rout PhD), Department of Analytical & Applied Economics (P Sahoo MA), UGC Centre of Advanced Study in Psychology (Prof M Satpathy PhD), Utkal University, Bhubaneswar, India; Department of Labour (P Roy PhD), Directorate of Factories, Government of West Bengal, Kolkata, India; Cardiovascular Department (Prof A M A Saad MD), Zagazig University, Zagazig, Egypt; Faculty of Medicine (Z Saadatian PhD), Infectious Diseases Research Center (Z Saadatian PhD), Gonabad University of Medical Sciences, Gonabad, Iran; Department of Medicine (C J Sabet MA), Georgetown University, Washington, DC, USA; Operational Research Center in Healthcare (Prof U Saeed PhD), Near East University (NEU), Nicosia Cyprus, Turkiye; International Center of Medical Sciences Research (ICMSR), Islamabad, Pakistan (Prof U Saeed PhD); Faculty of Medicine, Bioscience and Nursing (S Safi PhD), MAHSA University, Selangor, Malaysia; Interdisciplinary Research Centre in Biomedical Materials (IRCBM) (S Safi PhD), COMSATS Institute of Information Technology, Lahore, Pakistan; Center for Global Health Research (Prof A Sahebkar PhD), Saveetha Dental College and Hospitals (S Selvaraj PhD, M Tovani-Palone PhD), Saveetha University, Chennai, India; Biotechnology Research Center (Prof A Sahebkar PhD), Department of Medical Informatics (S Tabatabaei PhD), Clinical Research Development Unit (S Tabatabaei PhD), Department of Medicine (A Yarahmadi PhD), Mashhad University of Medical Sciences, Mashhad, Iran; Department of Nutrition and Dietetics (Prof S Sajadi PhD), Cihan University, Erbil, Erbil, Iraq; Faculty of Pharmacy (Prof M A Saleh PhD), Mansoura University, Mansoura, Egypt; Institute of Epidemiology and Preventive Medicine (Y L Samodra PhD), National Taiwan University, Taipei, Taiwan; Benang Merah Research Center (BMRC), Minahasa Utara, Indonesia (Y L Samodra PhD); Department of Entomology (A M Samy PhD), Medical Ain Shams Research Institute (MASRI) (A M Samy PhD), Ain Shams University, Cairo, Egypt; Department of Food Processing Technology (T Sarkar PhD), West Bengal State Council of Technical Education, Malda, India; Department of Geriatric and Long Term Care (B Sathian PhD), Hamad Medical Corporation, Doha, Qatar; Faculty of Health & Social Sciences (B Sathian PhD), Bournemouth University, Bournemouth, United Kingdom; Udyam-Global Association for Sustainable Development, Bhubaneswar, India (Prof M Satpathy PhD); Department of Medical Informatics (J Saulam MSc), Kagawa University, Miki-cho, Japan; Food Processing and Nutrition (J Saulam MSc), Karnataka State Akkamahadevi Women's University, Vijayapura, India; Department of Public

Health Sciences (M Sawhney PhD), University of North Carolina at Charlotte, Charlotte, NC, USA; Department of Preventive and Social Medicine (G Saya MD), Jawaharlal Institute of Postgraduate Medical Education and Research, Puducherry, India; Dr. D. Y. Patil Dental College and Hospital (S Selvaraj PhD), Dr. D. Y. Patil Vidyapeeth, Pune, India; Department of Medicine (Y Sethi MD), Swami Vivekanand Subharti University, Meerut, India; National Heart, Lung, and Blood Institute (A Seylani MD), National Institutes of Health, Rockville, MD, USA; Department of Ophthalmology (J Shah BS), Weill Cornell Medicine, New York City, NY, USA; Public Health Division (A A Shaheen PhD), Department of Clinical and Community Pharmacy (Prof S H Zyoud PhD), An-Najah National University, Nablus, Palestine; Center for Medical and Bio-Allied Health Sciences Research (Prof M J Shahwan PhD), Ajman University, Ajman, United Arab Emirates; Independent Consultant, Karachi, Pakistan (M A Shaikh MD); Department of Hemato-oncology (A Sharma MD), Fortis Hospital, Noida, India; K S Hegde Medical Academy (Prof M Shetty MD), Nitte University, Mangalore, India; National Institute of Infectious Diseases, Tokyo, Japan (M Shigematsu PhD); Department of Veterinary Public Health and Preventive Medicine (A Shittu MSc), Usmanu Danfodiyo University, Sokoto, Sokoto, Nigeria; Department of Medical Microbiology and Infectious Diseases (E E Siddig MD), Erasmus University, Rotterdam, Netherlands; Anthropological Survey of India (M Sikdar PhD), Anthropological Survey of India, Mysore, Karnataka, India; School of Medicine (Prof J A Singh MD), Baylor College of Medicine, Houston, TX, USA; Department of Medicine Service (Prof J A Singh MD), US Department of Veterans Affairs (VA), Houston, TX, USA; Department of Human Genetics (P Singh PhD), Punjabi University, Patiala, India; Associação de Profissionais Licenciados de Optometria (Advancing Eye Health Initiative), Braga, Portugal (R A Sousa MSc); Department of Public Health and Community Medicine (Prof C T Sreeramareddy MD), International Medical University, Kuala Lumpur, Malaysia; Medical and Diagnostic Research centre (Prof C T Sreeramareddy MD), University of Hail, hail, Saudi Arabia; Department of Clinical Research and Development (Prof L Szarpak PhD), LUXMED Group, Warsaw, Poland; Collegium Medicum (Prof L Szarpak PhD), John Paul II Catholic University of Lublin, Lublin, Poland; Department of Surgery (K Tan PhD), National University of Singapore, Singapore, Singapore; School of Population and Global Health (Prof H R Taylor MD), University of Melbourne, Carlton, VIC, Australia; Pediatric Intensive Care Unit (Prof M Temsah MD), King Saud University, Riyadh, Saudi Arabia; School of Humanities and Social Sciences (R Thakur PhD), Indian Institute of Technology Mandi, Mandi, India; Faculty of Public Health (J H V Ticoalu MPH), Universitas Sam Ratulangi (Sam Ratulangi University), Manado, Indonesia; School of Biomedical Engineering (N Tran MD), University of Technology Sydney, Sydney, NSW, Australia; Department of Business Analytics (T H Tran MD), University of Massachusetts Dartmouth, Dartmouth, MA, USA; Department of Internal Medicine (M Tumurkhuu PhD), Wake Forest University, Winston-Salem, NC, USA; International Center for Chemical and Biological Sciences (S Ullah MSc), University of Karachi, Karachi, Pakistan; Medical Genomics Research Department (Prof M Umair PhD), King Abdullah International Medical Research Center, Riyadh, Saudi Arabia; Department of Life Sciences (Prof M Umair PhD), University of Management and Technology, Lahore, Pakistan; Department of Artificial Intelligence (S Wang PhD), Xiamen University, Xiamen, China; Key Laboratory of Computer-Aided Drug Design (M Waqas PhD), Guangdong Medical University, Dongguan, China; Department of Biotechnology and Genetic Engineering (M Waqas PhD), Hazara University Mansehra, Mansehra, Pakistan; Department of Community Medicine (N D Wickramasinghe MD), Rajarata University of Sri Lanka, Anuradhapura, Sri Lanka; Department of Public Health (Prof K Yamagishi MD, Prof N Yonemoto PhD), Juntendo University, Tokyo, Japan; Department of Public Health Medicine (Prof K Yamagishi MD), University of Tsukuba, Tsukuba, Japan; Department of Medicine, Sidney Kimmel Medical College (A Yarahmadi PhD), Thomas Jefferson University, Philadelphia, PA, USA; National Center for Chronic and Noncommunicable Disease Control and Prevention (P Ye PhD), Chinese Center for Disease Control and Prevention, Beijing, China; Department of Health Management (A Yiğit PhD), Süleyman Demirel Üniversitesi (Süleyman Demirel University), Isparta, Turkiye; Pharmacy Department (Y E E Yismaw MSc), Alkan Health Science, Business and Technology College, Bahir Dar, Ethiopia;

Department of Biostatistics (Prof N Yonemoto PhD), University of Toyama, Toyama, Japan; Sant'Elia Hospital (A Zanghì MD), University of Catania, Caltanissetta, Italy; Noor Ophthalmology Research Center (M Ziafati MD), Noor Eye Hospital, Tehran, Iran; Clinical Research Centre (Prof S H Zyoud PhD), An-Najah National University Hospital, Nablus, Palestine; Department of Pediatrics (Prof J Shin MD), Yonsei University, Seoul, South Korea; Department of Pediatrics (Prof D Yon MD), Kyung Hee University, Seoul, South Korea

Authors' Contributions

Providing data or critical feedback on data sources

Yohannes Habtegiorgis Abate, Samar Abd ElHafeez, Auwal Abdullahi, Richard Gyan Aboagye, Lucas Guimarães Abreu, Ahmed Abu-Zaid, Habeeb Omoponle Adewuyi, Saira Afzal, Sajjad Ahmad, Ali Ahmadi, Ayman Ahmed, Haroon Ahmed, Amjad S Al Mosa, Rasmieh Mustafa Al-amer, Mohammed Albashtawy, Abdelazeem M Algammal, Abid Ali, Syed Shujait Ali, Awais Altaf, Sofia Androudi, Saeid Anvari, Jalal Arabloo, Anton A Artamonov, Akram M Asbeutah, Seyyed Shamsadin Athari, Maha Moh'd Wahbi Atout, Alok Atreya, Ahmed Y. Azzam, Zarrin Basharat, Mohammad-Mahdi Bastan, Akshaya Srikanth Bhagavathula, Sonu Bhaskar, Ajay Nagesh Bhat, Jasvinder Singh Bhatti, Fassikaw Kebede Bizuneh, Paul Svitil Briant, Fan Cao, Vijay Kumar Chattu, Hitesh Chopra, Dinh-Toi Chu, João M Coelho, Natalia Cruz-Martins, Xiaochen Dai, Samuel Demissie Darcho, Anna Dastiridou, Nikolaos Dervenis, Vinoth Gnana Chellaiyan Devanbu, Michael J Diaz, Thanh Chi Do, Ojas Prakashbhai Doshi, Michael Ekholuenetale, Rabie Adel El Arab, Ibrahim Farahat El Bayoumy, Chadi Eltaha, Mohammad Hassan Emamian, Adeniyi Francis Fagbamigbe, Ayesha Fahim, Hossein Farrokhpour, Ali Fatehizadeh, Timur Fazylov, Alireza Feizkhah, Ginenus Fekadu, João M M Furtado, Sridevi G, Muktar A Gadanya, Balasankar Ganesan, Ravindra Kumar Garg, Rupesh K Gautam, Mahaveer Golechha, Pouya Goleij, Ayman Grada, Shi-Yang Guan, Sapna Gupta, Veer Bala Gupta, Vivek Kumar Gupta, Arvin Haj-Mirzaian, Golnaz Heidari, Nguyen Quoc Hoan, Mehdi Hosseinzadeh, Salman Hussain, Segun Emmanuel Ibitoye, Nayu Ikeda, Lalu Muhammad Irham, Sheikh Mohammed Shariful Islam, Mihajlo Jakovljevic, Talha Jawaid, Shubha Jayaram, Jost B Jonas, Charity Ehimwenma Joshua, Vidya Kadashetti, Neeti Kapoor, Soujanya Kaup, Gbenga A Kayode, Yousef Saleh Khader, Himanshu Khajuria, Ajmal Khan, Atulya Aman Khosla, Min Seo Kim, Yun Jin Kim, Adnan Kisa, Shivakumar KM, Kewal Krishan, Nithin Kumar, Satyajit Kundu, Chandrakant Lahariya, Dharmesh Kumar Lal, Caterina Ledda, Munjae Lee, Seung Won Lee, Wei-Chen Lee, Stephen S Lim, Xuefeng Liu, Zheng Feei Ma, Kashish Malhotra, Roy Rillera Marzo, Andrea Maugeri, Tesfahun Mekene Meto, Endalkachew Belayneh Melese, Nouh Saad Mohamed, Abdollah Mohammadian-Hafshejani, Ali H Mokdad, Rohith Motappa, Sumaira Mubarik, Yanjinlkham Munkhsaikhan, Ganesh R Naik, Shumaila Nargus, Zuhair S Natto, Biswa Prakash Nayak, Van Thanh Nguyen, Robina Khan Niazi, Syed Toukir Ahmed Noor, Mamoona Noreen, Fred Nugen, Bogdan Oancea, Andrew T Olagunju, Sok King Ong, Mayowa O Owolabi, Jagadish Rao Padubidri, Georgios D Panos, Shahina Pardhan, Romil R Parikh, Sungchul Park, Tae Hwan Park, Maja Pasovic, Shrikant Pawar, Arokiasamy Perianayagam, Jalandhar Pradhan, Jagadeesh Puvvula, Pankaja Raghav, Fakher Rahim, Vafa Rahimi-Movaghar, Pushp Lata Rajpoot, Sowmya J Rao, Salman Rawaf, Priyanka Roy, Aly M A Saad, Zahra Saadatian, Cameron John Sabet, Basema Ahmad Saddik, Umar Saeed, Sher Zaman Safi, Pragyan Monalisa Sahoo, S. Mohammad Sajadi, Abdallah M Samy, Brijesh Sathian, Maheswar Satpathy, Monika Sawhney, Siddharthan Selvaraj, Yashendra Sethi, Jaffer Shah, Amira A Shaheen, Samiah Shahid, Masood Ali Shaikh, Muhammad Aaqib Shamim, Javad Sharifi Rad, Vishal Sharma, Maryam Shayan, Mahabalesh Shetty, Jae Il Shin, Aminu Shittu, Negussie Boti Sidamo, Mithun Sikdar, Jasvinder A Singh, Paramdeep Singh, Chandrashekhar T Sreeramareddy, Chandan Kumar

Swain, Lukasz Szarpak, Seyyed Mohammad Tabatabaei, Ker-Kan Tan, Krishna Tiwari, Marcos Roberto Tovani-Palone, Munkhtuya Tumurkhuu, Muhammad Umair, Sanaz Vahdati, Pengpeng Ye, Naohiro Yonemoto, Abzal Zhumagaliuly, Magdalena Zielińska, Sa'ed H Zyoud

Developing methods or computational machinery

Aleksandr Y Aravkin, Xiaochen Dai, Jiawei He, Ali H Mokdad

Providing critical feedback on methods or results

Yohannes Habtegiorgis Abate, Samar Abd ElHafeez, Auwal Abdullahi, Richard Gyan Aboagye, Lucas Guimarães Abreu, Ahmed Abu-Zaid, Isaac Yeboah Addo, Habeeb Omoponle Adewuyi, Saira Afzal, Williams Agyemang-Duah, Aqeel Ahmad, Danish Ahmad, Sajjad Ahmad, Ali Ahmadi, Hooman Ahmadzadeh, Ali Ahmed, Ayman Ahmed, Haroon Ahmed, Syed Anees Ahmed, Amjad S Al Mosa, Rasmieh Mustafa Al-amer, Mohammed Albashtawy, Ahmad Samir Alfaar, Abdelazeem M Algammal, Abid Ali, Syed Shujait Ali, Waad Ali, Awais Altaf, Vera L Alves Carneiro, Yaser Mohammed Al-Worafi, Hany Aly, Sofia Androudi, Boluwatife Stephen Anuoluwa, Saeid Anvari, Anayochukwu Edward Anyasodor, Jalal Arabloo, Mosab Arafat, Demelash Areda, Anton A Artamonov, Akram M Asbeutah, Seyyed Shamsadin Athari, Maha Moh'd Wahbi Atout, Alok Atreya, Shahkaar Aziz, Ahmed Y. Azzam, Saeed Bahramian, Ruhai Bai, Atif Amin Baig, Soham Bandyopadhyay, Zarrin Basharat, Mohammad-Mahdi Bastan, Maryam Bemanalizadeh, Akshaya Srikanth Bhagavathula, Nikha Bhardwaj, Pankaj Bhardwaj, Sonu Bhaskar, Ajay Nagesh Bhat, Jasvinder Singh Bhatti, Fassikaw Kebede Bizuneh, Paul Svitil Briant, Gabrielle Britton, Yasser Bustanji, Zahid A Butt, Florentino Luciano Caetano dos Santos, Mehtap Çakmak Barsbay, Fan Cao, Vijay Kumar Chattu, Hitesh Chopra, Sonali Gajanan Choudhari, Dinh-Toi Chu, João M Coelho, Natalia Cruz-Martins, Omid Dadras, Xiaochen Dai, Samuel Demissie Darcho, Ana Maria Dascalu, Nihar Ranjan Dash, Mohsen Dashti, Anna Dastiridou, Nikolaos Dervenis, Aragaw Tesfaw Desale, Vinoth Gnana Chellaiyan Devanbu, Amol S Dhane, Daniel Diaz, Michael J Diaz, Thanh Chi Do, Ojas Prakashbhai Doshi, Hisham Atan Edinur, Ferry Efendi, Michael Ekholuenetale, Rabie Adel El Arab, Ibrahim Farahat El Bayoumy, Muhammed Elhadi, Chadi Eltaha, Mohammad Hassan Emamian, Adeniyi Francis Fagbamigbe, Ayesha Fahim, Hossein Farrokhpour, Ali Fatehizadeh, Alireza Feizkhah, Ginenus Fekadu, Florian Fischer, Ida Fitriana, Matteo Foschi, João M M Furtado, Blima Fux, Sridevi G, Muktar A Gadanya, Abhay Motiramji Gaidhane, Aravind P Gandhi, Balasankar Ganesan, Ravindra Kumar Garg, Miglas Welay Gebregergis, Mesfin Gebrehiwot, Delaram J Ghadimi, Mahaveer Golechha, Ayman Grada, Shi-Yang Guan, Sapna Gupta, Veer Bala Gupta, Vivek Kumar Gupta, Roberth Steven Gutiérrez-Murillo, Awoke Derbie Habteyohannes, Arvin Haj-Mirzaian, Sobia Ahsan Halim, Ahmed I Hasaballah, Hamidreza Hasani, Golnaz Heidari, Mojtaba Heydari, Nguyen Quoc Hoan, Ramesh Holla, Sung Hwi Hong, Mehdi Hosseinzadeh, Chengxi Hu, Salman Hussain, Segun Emmanuel Ibitoye, Irena M Ilic, Milena D Ilic, Mustapha Immurana, Arit Inok, Lalu Muhammad Irham, Md. Rabiul Islam, Sheikh Mohammed Shariful Islam, Chidozie Declan Iwu, Louis Jacob, Ammar Abdulrahman Jairoun, Mihajlo Jakovljevic, Shubha Jayaram, Zixiang Ji, Jost B Jonas, Charity Ehimwenma Joshua, Vidya Kadashetti, Ankita Kankaria, Kehinde Kazeem Kanmodi, Neeti Kapoor, Ibraheem M Karaye, Soujanya Kaup, Gbenga A Kayode, Yousef Saleh Khader, Himanshu Khajuria, Atulya Aman Khosla, Min Seo Kim, Yun Jin Kim, Adnan Kisa, Shivakumar KM, Kewal Krishan, Mohammed Kuddus, Satyajit Kundu, Chandrakant Lahariya, Dharmesh Kumar Lal, Iván Landires, Van Charles Lansingh, Ariane Laplante-Lévesque, Caterina Ledda, Munjae Lee, Seung Won Lee, Wei-Chen Lee, Stephen S Lim, Xuefeng Liu, José Francisco López-Gil, Zheng Feei Ma, Kashish Malhotra, Vahid Mansouri, Roy Rillera Marzo, Alireza Mashaghi, Yasith Mathangasinghe, Andrea Maugeri, Asim Mehmood, Tesfahun Mekene Meto, Hadush Negash Meles, Endalkachew Belayneh Melese, Tomislav Mestrovic, Sachith Mettananda, Irmina Maria Michalek, Andreea Mirica, Abdalla Z Mohamed, Nouh Saad Mohamed, Abdollah Mohammadian-Hafshejani, Ali H Mokdad, Fateme Montazeri, Maryam

Moradi, Rohith Motappa, Sumaira Mubarik, Kavita Munjal, Yanjinlkham Munkhsaikhan, Amin Nabavi, Ganesh R Naik, Vinay Nangia, Shumaila Nargus, Zuhair S Natto, Muhammad Naveed, Biswa Prakash Nayak, Athare Nazri-Panjaki, Van Thanh Nguyen, Robina Khan Niazi, Syed Toukir Ahmed Noor, Mamoona Noreen, Fred Nugen, Bogdan Oancea, Osamudiamen Cyril Obasuyi, Andrew T Olagunju, Michal Ordak, Mayowa O Owolabi, Jagadish Rao Padubidri, Georgios D Panos, Shahina Pardhan, Romil R Parikh, Sungchul Park, Tae Hwan Park, Maja Pasovic, Roberto Passera, Jay Patel, Shrikant Pawar, Prince Peprah, Arokiasamy Perianayagam, Jalandhar Pradhan, Jagadeesh Puvvula, Nameer Hashim Qasim, Venkatraman Radhakrishnan, Pankaja Raghav, Fakher Rahim, Vafa Rahimi-Movaghar, Mosiur Rahman, Muhammad Aziz Rahman, Mohammad Rahmanian, Pushp Lata Rajpoot, Sowmya J Rao, Mohammad-Mahdi Rashidi, Salman Rawaf, Elrashdy M. Moustafa Mohamed Redwan, Mohsen Rezaeian, Sara Riaz, Moustaq Karim Khan Rony, Himanshu Sekhar Rout, Priyanka Roy, Aly M A Saad, Zahra Saadatian, Cameron John Sabet, Basema Ahmad Saddik, Umar Saeed, Sare Safi, Sher Zaman Safi, Fatemeh Saheb Sharif-Askari, Narjes Saheb Sharif-Askari, Pragyan Monalisa Sahoo, S. Mohammad Sajadi, Mohamed A Saleh, Yoseph Leonardo Samodra, Abdallah M Samy, Tanmay Sarkar, Brijesh Sathian, Maheswar Satpathy, Jennifer Saulam, Monika Sawhney, Ganesh Kumar Saya, Siddharthan Selvaraj, Yashendra Sethi, Jaffer Shah, Amira A Shaheen, Samiah Shahid, Masood Ali Shaikh, Muhammad Aaqib Shamim, Javad Sharifi Rad, Anupam Sharma, Vishal Sharma, Maryam Shayan, Mika Shigematsu, Jae Il Shin, Aminu Shittu, Negussie Boti Sidamo, Emmanuel Edwar Siddig, Mithun Sikdar, Jasvinder A Singh, Paramdeep Singh, Puneetpal Singh, Raul A R C Sousa, Chandrashekhar T Sreeramareddy, Chandan Kumar Swain, Lukasz Szarpak, Seyyed Mohammad Tabatabaei, Ker-Kan Tan, Hugh R Taylor, Mohamad-Hani Temsah, Ramna Thakur, Jansje Henny Vera Ticoalu, Krishna Tiwari, Marcos Roberto Tovani-Palone, Ngoc Ha Tran, Munkhtuya Tumurkhuu, Saeed Ullah, Muhammad Umair, Sanaz Vahdati, Shaopan Wang, Muhammad Waqas, Nuwan Darshana Wickramasinghe, Amir Yarahmadi, Pengpeng Ye, Arzu Yiğit, Yazachew Engida Engida Yismaw, Dong Keon Yon, Naohiro Yonemoto, Mohammed G M Zeariya, Claire Chenwen Zhong, Magdalena Zielińska, Sa'ed H Zyoud

Drafting the work or revising it critically for important intellectual content

Hasan Aalruz, Yohannes Habtegiorgis Abate, Rouzbeh Abbasgholizadeh, Samar Abd ElHafeez, Auwal Abdullahi, Lucas Guimarães Abreu, Ahmed Abu-Zaid, Isaac Yeboah Addo, Habeeb Omoponle Adewuyi, Saira Afzal, Danish Ahmad, Ali Ahmadi, Hooman Ahmadzadeh, Ali Ahmed, Ayman Ahmed, Haroon Ahmed, Syed Anees Ahmed, Amjad S Al Mosa, Rasmieh Mustafa Al-amer, Mohammed Albashtawy, Ahmad Samir Alfaar, Abdelazeem M Algammal, Fadwa Naji Alhalaiqa, Abid Ali, Syed Shujait Ali, Waad Ali, Ahmad Alrawashdeh, Awais Altaf, Yaser Mohammed Al-Worafi, Hany Aly, Sofia Androudi, Boluwatife Stephen Anuoluwa, Saeid Anvari, Anayochukwu Edward Anyasodor, Jalal Arabloo, Akram M Asbeutah, Seyyed Shamsadin Athari, Maha Moh'd Wahbi Atout, Alok Atreya, Lemessa Assefa A Ayana, Shahkaar Aziz, Ahmed Y. Azzam, Ruhai Bai, Atif Amin Baig, Soham Bandyopadhyay, Mohammad-Mahdi Bastan, Maryam Bemanalizadeh, Akshaya Srikanth Bhagavathula, Sonu Bhaskar, Ajay Nagesh Bhat, Jasvinder Singh Bhatti, Fassikaw Kebede Bizuneh, Gabrielle Britton, Yasser Bustanji, Florentino Luciano Caetano dos Santos, Mehtap Çakmak Barsbay, Vijay Kumar Chattu, Anis Ahmad Chaudhary, Patrick R Ching, Hitesh Chopra, João M Coelho, Natalia Cruz-Martins, Emanuele D'Amico, Samuel Demissie Darcho, Ana Maria Dascalu, Nihar Ranjan Dash, Nikolaos Dervenis, Amol S Dhane, Daniel Diaz, Michael J Diaz, Thanh Chi Do, Ashel Chelsea Dsouza, Michael Ekholuenetale, Rabie Adel El Arab, Ibrahim Farahat El Bayoumy, Muhammed Elhadi, Chadi Eltaha, Mohammad Hassan Emamian, Adeniyi Francis Fagbamigbe, Ayesha Fahim, Hossein Farrokhpour, Ali Fatehizadeh, Nuno Ferreira, Florian Fischer, Ida Fitriana, Ali Forouhari, Matteo Foschi, João M M Furtado, Blima Fux, Sridevi G, Muktar A Gadanya, Balasankar Ganesan, Ravindra Kumar Garg, Rupesh K Gautam, Miglas Welay Gebregergis, Delaram J Ghadimi, Sadegh Ghafarian, Ayman Grada, Shi-Yang Guan, Snigdha Gulati, Sapna Gupta, Roberth Steven Gutiérrez-Murillo, Awoke Derbie Habteyohannes, Arvin Haj-Mirzaian, Sobia Ahsan Halim, Ahmed I Hasaballah, Md. Kamrul Hasan, Hamidreza Hasani, Golnaz Heidari, Mojtaba Heydari, Nguyen Quoc Hoan, Ramesh Holla, Sung Hwi Hong, Chengxi Hu, Junjie Huang, Salman

Hussain, Segun Emmanuel Ibitoye, Irena M Ilic, Milena D Ilic, Mustapha Immurana, Arit Inok, Lalu Muhammad Irham, Md. Rabiul Islam, Sheikh Mohammed Shariful Islam, Chidozie Declan Iwu, Louis Jacob, Mihajlo Jakovljevic, Shubha Jayaram, Yi Deun Jeong, Jost B Jonas, Nitin Joseph, Charity Ehimwenma Joshua, Vidya Kadashetti, Kehinde Kazeem Kanmodi, Neeti Kapoor, Soujanya Kaup, Gbenga A Kayode, Yousef Saleh Khader, Himanshu Khajuria, Ajmal Khan, Atulya Aman Khosla, Min Seo Kim, Yun Jin Kim, Adnan Kisa, Shivakumar KM, Kewal Krishan, Mohammed Kuddus, Mukhtar Kulimbet, Chandrakant Lahariya, Iván Landires, Caterina Ledda, Wei-Chen Lee, José Francisco López-Gil, Zheng Feei Ma, Kashish Malhotra, Vahid Mansouri, Roy Rillera Marzo, Yasith Mathangasinghe, Andrea Maugeri, Asim Mehmood, Hadush Negash Meles, Endalkachew Belayneh Melese, Tomislav Mestrovic, Sachith Mettananda, Irmina Maria Michalek, Abdalla Z Mohamed, Nouh Saad Mohamed, Abdollah Mohammadian-Hafshejani, Ali H Mokdad, Fateme Montazeri, Maryam Moradi, Yanjinlkham Munkhsaikhan, Amin Nabavi, Zuhair S Natto, Biswa Prakash Nayak, Van Thanh Nguyen, Robina Khan Niazi, Mamoona Noreen, Fred Nugen, Bogdan Oancea, Andrew T Olagunju, Michal Ordak, Verner N Orish, Mayowa O Owolabi, Jagadish Rao Padubidri, Leonidas D Panos, Shahina Pardhan, Romil R Parikh, Seoyoung Park, Tae Hwan Park, Roberto Passera, Jay Patel, Shrikant Pawar, Arokiasamy Perianayagam, Mohsen Pourazizi, Jalandhar Pradhan, Jagadeesh Puvvula, Venkatraman Radhakrishnan, Pankaja Raghav, Fakher Rahim, Vafa Rahimi-Movaghar, Mohammad Hifz Ur Rahman, Shayan Rahmani, Mohammad Rahmanian, Sowmya J Rao, Salman Rawaf, Elrashdy M. Moustafa Mohamed Redwan, Sara Riaz, Priyanka Roy, Aly M A Saad, Cameron John Sabet, Basema Ahmad Saddik, Umar Saeed, Fatemeh Saheb Sharif-Askari, Amirhossein Sahebkar, Abdallah M Samy, Tanmay Sarkar, Maheswar Satpathy, Ganesh Kumar Saya, Siddharthan Selvaraj, Yashendra Sethi, Allen Seylani, Jaffer Shah, Samiah Shahid, Moyad Jamal Shahwan, Muhammad Aaqib Shamim, Javad Sharifi Rad, Anupam Sharma, Vishal Sharma, Mahabalesh Shetty, Pavanchand H Shetty, Premalatha K Shetty, Mika Shigematsu, Jae Il Shin, Aminu Shittu, Negussie Boti Sidamo, Emmanuel Edwar Siddig, Mithun Sikdar, Jasvinder A Singh, Paramdeep Singh, Surjit Singh, Raul A R C Sousa, Chandrashekhar T Sreeramareddy, Chandan Kumar Swain, Lukasz Szarpak, Ker-Kan Tan, Mohamad-Hani Temsah, Ramna Thakur, Krishna Tiwari, Marcos Roberto Tovani-Palone, Thang Huu Tran, Muhammad Umair, Sanaz Vahdati, Shaopan Wang, Nuwan Darshana Wickramasinghe, Kazumasa Yamagishi, Amir Yarahmadi, Arzu Yiğit, Dong Keon Yon, Naohiro Yonemoto, Aurora Zanghì, Mohammed G M Zeariya, Zhi-Jiang Zhang, Claire Chenwen Zhong, Makan Ziafati, Magdalena Zielińska, Sa'ed H Zyoud

Managing the estimation or publications process

Sung Hwi Hong, Yi Deun Jeong, Min Seo Kim, Ali H Mokdad, Seoyoung Park, Maja Pasovic, and Dong Keon Yon
